# Understanding the conservation-genetics gap in Latin America: challenges and opportunities to integrate genetics into conservation practices

**DOI:** 10.3389/fgene.2024.1425531

**Published:** 2024-07-08

**Authors:** Constanza Napolitano, Cristhian Clavijo, Viviana Rojas-Bonzi, Carolina I. Miño, José F. González-Maya, Nadia Bou, Alan Giraldo, Angela Martino, Cristina Yumi Miyaki, Luis F. Aguirre, Andrea Cosacov, Yoamel Milián-García, Laura Prosdocimi, O. Eric Ramírez-Bravo, Luis Antonio Tovar, Ximena Velez-Zuazo, Mercedes Barrios, Bernal Herrera-Fernández, María G. Montiel-Villalobos, María A. Oliveira-Miranda, Monique Pool, Alonso Santos-Murgas, Maria Claudia Segovia-Salcedo, Felipe Cecchi, Armando J. Dans, Nelanie Dilchand, Sergio M. Q. Lima, María Caridad Novas, Karla Pelz-Serrano, Nina Pougy, Iris Rodríguez, Liesbeth van der Meer, Galo Zapata-Ríos

**Affiliations:** ^1^ Departamento de Ciencias Biológicas y Biodiversidad, Universidad de Los Lagos, Osorno, Chile; ^2^ Institute of Ecology and Biodiversity, Concepción, Chile; ^3^ Cape Horn International Center, Puerto Williams, Chile; ^4^ Vida Silvestre Uruguay, Montevideo, Uruguay; ^5^ Instituto de Investigación Biológica del Paraguay, Asuncion, Paraguay; ^6^ Wildlife Ecology and Conservation Department, University of Florida, Gainesville, FL, United States; ^7^ Laboratorio de Genética Evolutiva - LGE, Instituto de Biología Subtropical - IBS, Consejo Nacional de Investigaciones Científicas y Técnicas - CONICET, Universidad Nacional de Misiones (UNaM), Posadas, Argentina; ^8^ Área de Biología de la Conservación, Departamento de Ciencias Ambientales, División de Ciencias Biológicas y de la Salud, Universidad Autónoma Metropolitana Unidad Lerma, Bogotá, Colombia; ^9^ Departamento de Biodiversidad y Genética, Instituto de Investigaciones Biológicas Clemente Estable, Montevideo, Uruguay; ^10^ Grupo de Investigación en Ecología Animal, Departamento de Biología, Facultad de Ciencias Naturales y Exactas, Universidad del Valle, Cali, Colombia; ^11^ Centro de Investigaciones en Ecología y Zonas Aridas, Universidad Nacional Experimental Francisco de Miranda, Coro, Venezuela; ^12^ Departamento de Genética e Biologia Evolutiva, Instituto de Biociências, Universidade de São Paulo, São Paulo, Brazil; ^13^ Centro de Biodiversidad y Genética, Universidad Mayor de San Simón, Cochabamba, Bolivia; ^14^ Laboratorio de Ecología Evolutiva y Biología Floral, Instituto Multidisciplinario de Biología Vegetal, CONICET-Universidad Nacional de Córdoba, Córdoba, Argentina; ^15^ Department of Integrative Biology, University of Guelph, Guelph, ON, Canada; ^16^ Laboratorio de Ecología, Comportamiento y Mamíferos Marinos (LECyMM), Museo Argentino de Ciencias Naturales (MACN-CONICET), Buenos Aires, Argentina; ^17^ Centro de Agroecología, Instituto de Ciencias, Benemérita Universidad Autónoma de Puebla, Eco campus Valsequillo, San Pedro Zacachimalpa, Mexico; ^18^ Facultad de Ciencias Forestales, Universidad Nacional Agraria La Molina, Lima, Peru; ^19^ Smithsonian National Zoological Park and Conservation Biology Institute, Washington, DC, United States; ^20^ Centro de Datos para la Conservación, Centro de Estudios Conservacionistas, Universidad de San Carlos de Guatemala, Guatemala City, Guatemala; ^21^ Instituto Internacional de Conservación y Manejo de Vida Silvestre (ICOMVIS), Universidad Nacional, Heredia, Costa Rica; ^22^ Red Latinoamericana de Genética para la Conservación (ReGeneC), Baja California Sur, La Paz, Mexico; ^23^ Red Latinoamericana de Genética para la Conservación (ReGeneC), Caracas, Venezuela; ^24^ Green Heritage Fund Suriname, Paramaribo, Suriname; ^25^ Departamento de Zoología, Facultad de Ciencias Naturales Exactas y Tecnología, Universidad de Panamá, Ciudad de Panamá. Estación Científica Coiba AIP, Ciudad del Saber, Panama; ^26^ Departamento de Ciencias de la Vida y la Agricultura, Universidad de las Fuerzas Armadas -ESPE, Sangolquí, Ecuador; ^27^ Grupo Antropología de la Conservación, Universidad de Los Lagos, Osorno, Chile; ^28^ Departamento de Ciencias Ambientales y Producción Sostenible, Universidad de las Regiones Autónomas de la Costa Caribe Nicaragüense, Bluefields, Nicaragua; ^29^ Aquatic and Terrestrial Pioneers Consulting Services, Georgetown, Guyana; ^30^ Departamento de Botânica e Zoologia, Universidade Federal do Rio Grande do Norte, Natal, Brazil; ^31^ División de Conservación, Departamento de Botánica, Jardín Botánico Nacional Dr. Rafael María Moscoso, Santo Domingo, Dominican Republic; ^32^ Departamento de Ciencias Ambientales, División de Ciencias Biológicas y de la Salud, Universidad Autónoma Metropolitana Unidad Lerma, Lerma, Mexico; ^33^ Departamento de Desenvolvimento Científico, Museu do Amanhã, Instituto de Desenvolvimento e Gestão - IDG, Rio de Janeiro, Brazil; ^34^ Escuela de Biología, Universidad Nacional Autónoma de Honduras, Tegucigalpa, Honduras; ^35^ Oceana Chile, Santiago, Chile; ^36^ Wildlife Conservation Society - Ecuador Program, Quito, Ecuador

**Keywords:** conservation management practices, conservation managers, genetic researchers, partnerships, local knowledge, endangered species, important conservation areas

## Abstract

**Introduction:** Integrating genetic data into conservation management decisions is a challenging task that requires strong partnerships between researchers and managers. Conservation in Latin America is of crucial relevance worldwide given the high biodiversity levels and the presence of hotspots in this region.

**Methods:** We conducted a survey across Latin America to identify gaps and opportunities between genetic researchers and conservation managers. We aimed to better understand conservation managers’ points of view and how genetic research could help conservation practitioners to achieve their goals, by implementing genetic assessments that could effectively inform conservation practices. We distributed an online survey via four regional collaborating organizations and 32 focal points based in 20 Latin American countries. The target respondents were conservation managers of species or areas in Latin America.

**Results:** We collected a total of 468 answered questionnaires from 21 Latin American countries. Most respondents (44%) were from an academic or research institution while non-academics were mainly from non-governmental institutions (30%) and government agencies (25%). Most respondents (65%) have performed or used genetic assessments in their managed area or species, either alone, in partnership, contracting someone else or using published results. For the majority of this group, the genetic results were relevant to their conservation management goals, helping to inform management decisions. Respondents that had not performed genetic assessments (35%) were mainly from the non-academic group, and their main barriers were limited access to funds, genetic lab facilities, and trained personnel to design studies and conduct lab work.

**Discussion:** From the findings, we describe the current situation and provide a general diagnosis of the conservation-genetics gap in Latin America. We describe the gender gap, academic-practitioner co-development of conservation questions and projects, and the nationality and residency of Latin American conservation managers in relation to the countries where they work. We discuss opportunities to co-create research questions and co-develop studies based on conservation practitioners’ needs. We offer recommendations for overcoming barriers to integrate genetic information into conservation actions, and advance agendas that fit the needs and realities of the highly heterogeneous, biodiverse and challenging Latin American region.

## Introduction

To respond to the current biodiversity crisis prompted by human-induced global change (Ceballos et al., 2020), it is essential to integrate and translate scientific evidence into socio-political frameworks, key decision-making processes and public policies (Díaz et al., 2019). This urgent mission demands a broad and interdisciplinary view from researchers, conservation managers, decision makers, politicians, lawmakers and all stakeholders in general, who should collaborate to seek common research goals and indicators, and to apply measures to preserve biodiversity (Ferreira and Klütsch, 2021; Kershaw et al., 2022; Willi et al., 2022).

Among the components of biodiversity, genetic diversity plays a crucial role in ecosystem resilience, species survival, and adaptation (Taylor et al., 2017; Díaz et al., 2019; Laikre et al., 2020; Taft et al., 2020; Hoban, et al., 2021a). Genetic data provide unique insights into the conservation status and trends for species and populations (Willoughby et al., 2015; Pierson et al., 2016; Torres-Florez et al., 2018; Garner et al., 2020). However, despite its recognized importance, incorporating genetic information into conservation management strategies and policies continues to be a global challenge (Laikre, 2010; Laikre et al., 2010; 2016; Shafer et al., 2015; Cook and Sgrò, 2017; Cook and Sgrò, 2018; Sandström et al., 2019; Hoban et al., 2020; Hoban et al., 2021b; Taft et al., 2020; Galetti, 2023), contributing to what is known as the ‘conservation-genetics gap’ (Taylor et al., 2017).

Studies that have analyzed the conservation-genetics gap have identified four major barriers to include genetic data into conservation management: i) the lack of an efficient mechanism for knowledge transfer among and between academics and stakeholders (Hoban et al., 2013; Taylor et al., 2017; Britt et al., 2018; Taft et al., 2020; Klütsch and Laikre, 2021), ii) the lack of formal training to interpret and assess genetic data among managers (Haig et al., 2016; Cook and Sgrò, 2017; Cook and Sgrò, 2018; Taylor et al., 2017; Taft et al., 2020), iii) the perceived cost by managers and the funding needed to conduct genetic studies (Taylor et al., 2017; Klütsch and Laikre, 2021) and iv) bureaucratic structures or restrictions of the relevant governmental institutions (Rojas Bonzi et al., 2018; Klütsch and Laikre, 2021). These barriers may be exacerbated in extensive, highly diverse and culturally heterogeneous regions such as Latin America. This geographic region harbors an important proportion of the Earth’s total biodiversity (≈40%; UNEP-WCMC, 2016), includes various biodiversity hotspots (Myers et al., 2000; Mittermeier et al., 2011), and faces severe and increasing habitat degradation and a rapid decline of wild populations (Myers et al., 2000; Rodríguez-Clark et al., 2015; UNEP-WCMC, 2016; Josse and Fernandez, 2021).

Genetic information has been increasingly used for conservation in Latin America (Torres-Florez et al., 2018; Moraes et al., 2023) with Brazil, Mexico and Chile leading the field in terms of the number of published studies (Rodríguez-Clark et al., 2015). These studies mainly focus on terrestrial species (Oliveira-Miranda et al., 2013; Torres-Florez et al., 2018), population genetics and genetic diversity issues, and on assessing the genetic consequences of overexploitation and habitat loss (Torres-Florez et al., 2018). While several of these articles have included specific recommendations for conservation and management actions, the extent to which these suggestions or other genetic findings have been effectively integrated into conservation strategies, management actions and/or formal policies across Latin America remains unclear (Torres-Florez et al., 2018), particularly in countries with limited scientific production. This understanding is pivotal for unraveling the drivers and factors that perpetuate the conservation-genetics gap throughout Latin America.

In the present study, we specifically aimed to characterize the conservation-genetics gap in Latin America, by comprehensively investigating the relationship between genetic research and management for conservation in the region. We aimed to identify and understand the gaps that need to be bridged, and the challenges and opportunities faced by the Latin American conservation community in order to integrate genetics into conservation practice. We based our study on that performed by Taft et al. (2020) who explored the barriers preventing the use of genetic data for conservation practice and policy by surveying conservation practitioners from diverse academic and government institutions (n = 50). However, their findings predominantly come from a geographically-biased sample, primarily from the United States of America. Hence, the insights derived from Taft et al. (2020) are not directly transferable and cannot be generalized to assess the effective use of genetic information in management practices for conservation in the highly heterogeneous region of Latin America.

Therefore, our goals are, i) to identify gaps and opportunities for collaboration between genetic researchers and practitioners aimed at implementing genetic assessments (at the population, species and ecosystem levels) that can inform conservation management in Latin America; and ii) to understand how researchers and conservation practitioners can collaborate effectively and engage in fruitful partnerships to achieve common conservation goals in the region. We assess the main barriers and discuss recommendations and perspectives for the co-creation and co-development of studies specifically tailored to suit the needs of conservation practitioners, stakeholders and/or local communities, to thus encourage the effective integration of genetic information into conservation actions in the biodiversity-rich Latin American region.

## Methods

### Study area

We intended to assess 20 countries in the Austral and Neotropical Americas (ANA) which includes Mexico, Central America, the Caribbean (including Guyana, Suriname, French Guiana and the Caribbean islands) and South America. This area is politically known as Latin America, whose countries possess similar or common socioeconomic histories and scenarios (Ceballos et al., 2009; Torres-Florez et al., 2018).

### Survey distribution and application

To select our target respondents we used purposeful sampling, a technique implemented to identify and select individuals or groups of individuals that are especially knowledgeable about or experienced in a phenomenon of interest (Cresswell and Plano Clark, 2011). To achieve a representative sample size and a homogeneous distribution of sampling effort across Latin America, we worked with 32 focal points (63% female; 38% male) from each of the 20 targeted countries. We defined a focal point as a person who works in conservation in a Latin American country, and whose role was to identify and contact other target respondents, distribute the surveys, and perform follow-ups. The number of focal points per country (1–3) was established according to the total population of the country and standardized by the number of researchers per country (expressed as per million) based on UNESCO Institute for Statistics (UIS) data in the World Bank DataBank (UNESCO, 2021). In addition to direct sampling through focal points, we also distributed the survey using group mailing lists through the following collaborating organizations: The Southern Cone of South America Chapter of the Society for Conservation Biology (SCB), the Latin American Conservation Genetics Network (ReGenec), the Latin America and the Caribbean Section of the SCB (LACA-SCB) and the Mesoamerican Society for Biology and Conservation.

### Target respondents

Target respondents were conservation managers in Latin America, directly and recently involved (i.e., within the last 5 years; 2017–2021) with the conservation of a species or area, either through planning conservation strategies (i.e., species action plans), management supervision, species monitoring, or evaluating the outcome of such practices. Scientists not directly involved in conservation management, or without on-the-ground experience in conservation, were not targeted as respondents.

### Survey design

We adapted the survey implemented by Taft et al. (2020) given our focus on Latin American managers and practitioners. Thus, we included additional questions based on specific aspects that we wanted to address in a regional context, such as parachute science (Haelewaters et al., 2021; Liboiron, 2021; Asase et al., 2022; de Vos and Schwartz, 2022; Horn et al., 2022) and the gender gap. The survey was translated into the four most common official languages spoken in the targeted geographical region: Spanish, Portuguese, English and Dutch. We used Google Forms (Google, 2021) to implement the survey. We collected responses during a 3-month time-frame (15 August-16 November 2021). The 15-min survey (Supplementary Appendix SI, SII, SIII) had no mandatory questions and all responses were recorded as anonymous. The 35 questions were grouped into four sections, as follows: (i) Respondent’s demographics, (ii) Area/species managed, (iii) Type of genetic study performed and, (iv) Partnerships with other research groups and/or conservation practitioners. Different question formats were used: multiple-choice (exclusive/non-exclusive; *n* = 25), five-point Likert scale (Likert, 1932; *n* = 5) and open-ended (*n* = 5). In the first section, we asked about nationality, gender, age, place of work, type of working institution, and role within the institution/organization. The second section inquired about study location, realm of the managed areas/species (i.e., marine, terrestrial, freshwater), and specific research and/or monitoring interests regarding their managed area/species. In the third section we asked about the usefulness of different types of genetic assessments, interest in conducting such assessments, information on those performed and potential barriers to conducting/using them. In the fourth section, we examined whether the respondent had to ask for experts’ assistance and whether they relied on experts. In addition, we assessed the effectiveness of our survey distribution and sampling methods by including a question where we asked how the survey had reached the respondent. We recorded the sampling effort (i.e., number of invitations sent) and measured sampling success by calculating answered surveys/effort. We compared survey distribution success between focal points and organizations’ mailing lists to explore the effectiveness of both survey distribution strategies.

We used descriptive statistics to summarize our results. The survey included an Informed Consent disclosure and was revised and approved by the Ethics Committee of the Institute of Ecology and Biodiversity (IEB, Chile) (Approval certificate 12 August 2021).

## Results

### Survey distribution and sampling success

We sent 2,196 invitations to potential participants in the survey, representing the total sampling effort. The sampling success varied depending on the method of survey distribution: invitations sent through focal points resulted in 383 responses (out of 1,344 invitations sent, with a response rate of 28.5%, accounting for 81.8% of the replies), while those sent via mailing lists from collaborating organizations yielded 79 responses (from 852 invitations, with a response rate of 9.3%, accounting for 16.9% of the replies). Additionally, 0.85% (4 responses) did not specify how they received the survey, while 0.43% (2 responses) reported receiving the survey through other means, namely, from a colleague and a local non-government organization (NGO).

### Respondents’ demographic characteristics

**FIGURE 1:**
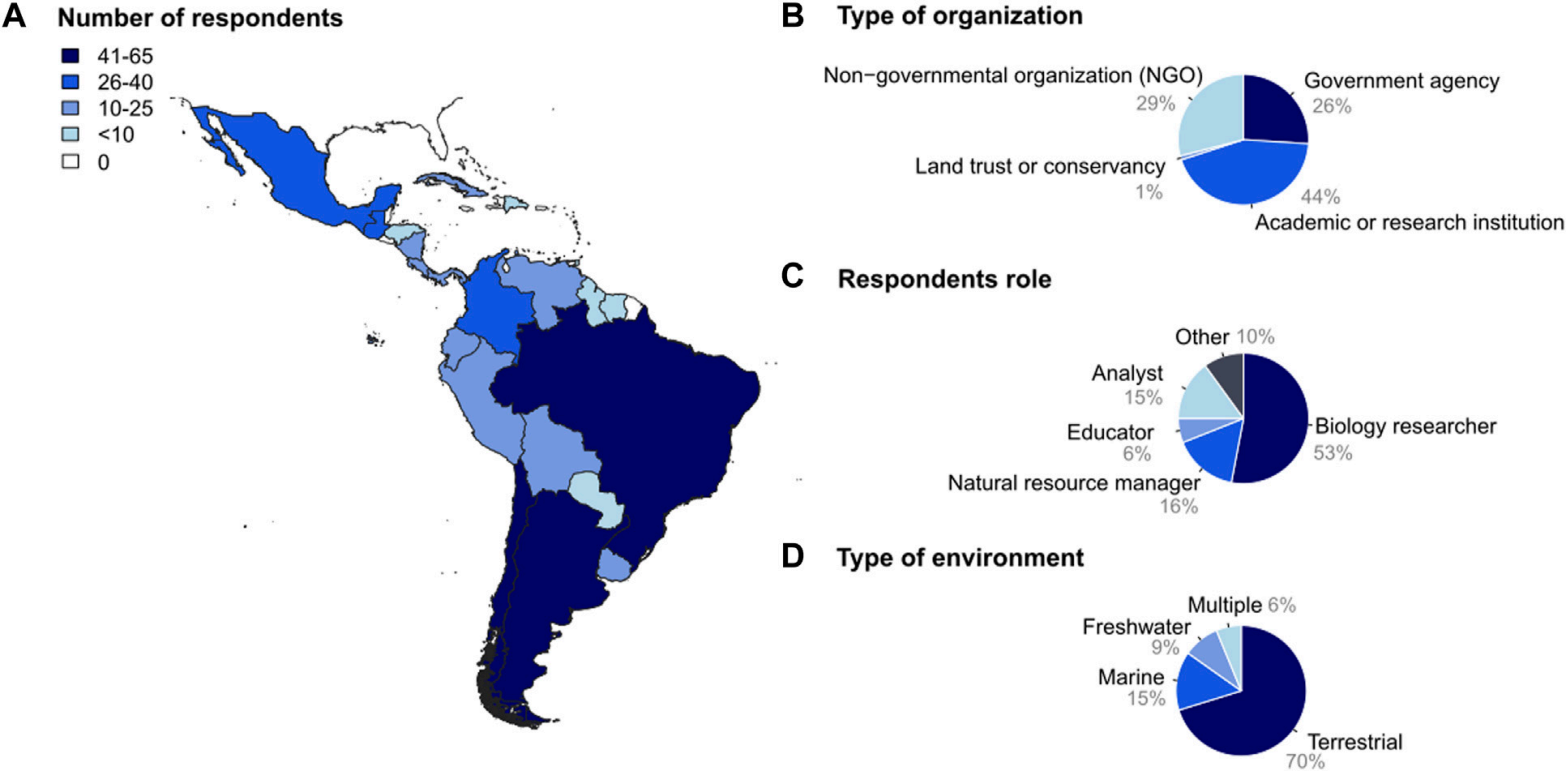
Characteristics of conservation managers in Latin America based on 468 survey respondents. **(A)** Latin America political map with countries color coded by the number of respondents. **(B)** Respondents’ organization type. **(C)** Respondents’ role in their respective organizations. Biology researcher (lab or fieldwork); Analyst (decision maker in public policy, legislation, strategic planning). **(D)** Type of environment where the respondent’s managed area/species is located.

In all, we received 468 responses from conservation managers working in 21 Latin American countries (Figure 1A). Fifty-five percent of respondents identified as males, 43% as females, 1% preferred not to declare their gender identity, and 1% did not answer (0% Other; Figure 4A). Most respondents were 40–49 years old (35%), followed by 30–39 (28%), 50-59 (21%), >60 (9%), <30 (5%) and 1% did not answer this question. Most respondents worked in an academic or research institution (44%), followed by an NGO (29%), governmental agency (26%) and land trust or conservancy (1%) (Figure 1B). Regarding their role within their respective organization, most respondents declared being biology researchers (lab or fieldwork; 53%), followed by natural resource managers (16%), analysts (15%; decision makers in public policy, legislation and/or strategic planning), other (10%), and educators (6%) (Figure 1C). Concerning the position within their organization, most respondents declared to be of mid-level executive work rank (researcher; 59%), followed by chief officer (director; 33%), and operative employee (student; 7%) (0.4% did not answer).

The vast majority of respondents (97%) were nationals from Latin America, followed by nationals from Europe (2%) and North America (1%). Regarding the country of residence and nationality of respondents, most were residents (92%) or nationals (92%) with respect to the country where the managed area/species is located, while 87% were both nationals and residents. A smaller percentage (2%, 10 respondents) were neither nationals nor residents of the country where the managed area/species is located. Of the latter group, 1.4% (7 respondents) were Latin Americans working in a different Latin American country from their country of origin, 0.4% were North Americans working in Latin America (2 respondents), and 1 respondent was a Latin American managing an area/species in Latin America but residing in North America (0.2%).

### Area/species managed and challenges identified

Respondents manage areas or species located in terrestrial (70%), marine (15%), and freshwater (9%) realms, whilst 6% manage areas or species in multiple realms (Figure 1D). Most respondents (69%) work in *in-situ* management (i.e., within the species’ natural habitat), 24% work both in *in-situ* and *ex-situ* management (i.e., outside the species’ natural habitat), while only 7% of the respondents work exclusively *ex-situ*. The main taxonomic groups studied using genetic assessment were animals (71%), followed by plants (13%), microorganisms (Bacteria, Archaea, Protist) (1%), fungi (1%) and 14% of respondents declared as having studied multiple taxa.

Respondents stated that the most significant challenges regarding their managed area/species were: (i) Assessing population size (very important 67%, important 21%); (ii) Maintaining connectivity or identifying corridors (very important 63%, important 20%); (iii) Identifying Management Units (very important 56%, important 25%); (iv) Delineating populations (very important 50%, important 25%); (v) Inventorying species (very important 45%, important 21%); (vi) Assessing life-history characteristics (very important 40%, important 26%); (vii) Assessing inbreeding or relatedness of individuals (very important 32%, important 18%), and (viii) Detecting/preventing hybridization (very important 23%, important 13%).

### Genetic studies in the managed area/species

Most respondents (79%) indicated that they had already considered using genetics to support their management practices, with 67% stating that they would know how to conduct a genetic assessment in their managed area/species if they were interested in doing so. Additionally, 25% reported having conducted a biodiversity inventory or identified species with DNA barcoding or environmental DNA techniques.

Regarding the usefulness of the genetic information, respondents considered it extremely useful or useful to: (i) Inform management actions (82%), (ii) Establish baseline information about the managed area (e.g., population census or species composition) (82%), (iii) Assess the effectiveness of management actions (75%), or (iv) Inform legislative protection or actions (71%).

**FIGURE 2:**
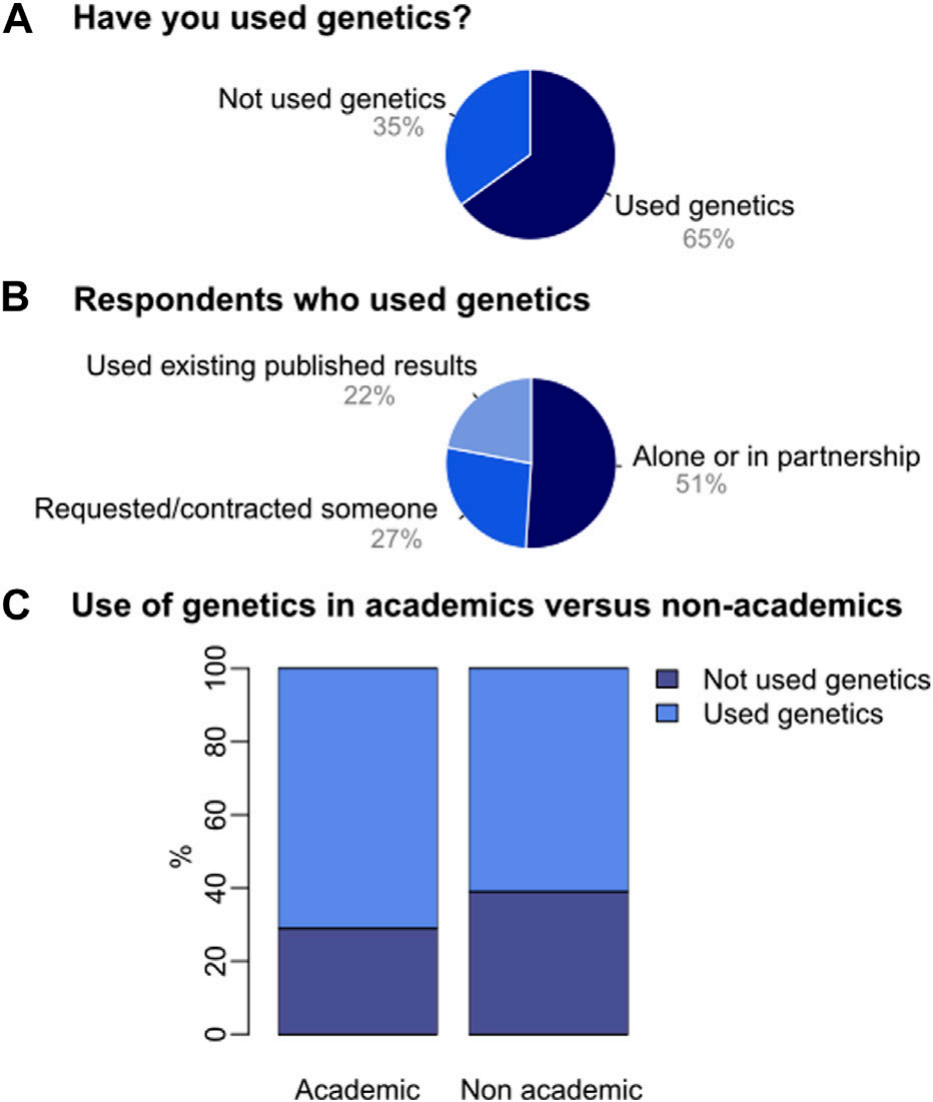
Use of genetic assessments by conservation managers in Latin America. **(A)** Respondents answer the question about whether or not they have used genetics in their managed areas/species. **(B)** Respondents answer the question about how they used genetics in their managed areas/species (conducted the study alone or in partnership; requested or contracted someone else to do the assessment, or used existing genetic published results). **(C)** Use of genetic assessments in academic *versus* non-academic respondents.

Most respondents (65%) declared having performed or used a genetic assessment in their managed area/species (Figure 2A). Of these, 51% performed the assessment alone or in collaboration, 27% requested/contracted someone else to conduct the assessment, and 22% used published genetic data (Figure 2B). The percentage of respondents who had used/performed a genetic assessment in their managed area/species was greater among academics (70%) compared to non-academics (60%). Conversely, the percentage of respondents who had not used/performed a genetic assessment was higher among non-academics (39%) than among academics (29%; Figure 2C).

**FIGURE 3:**
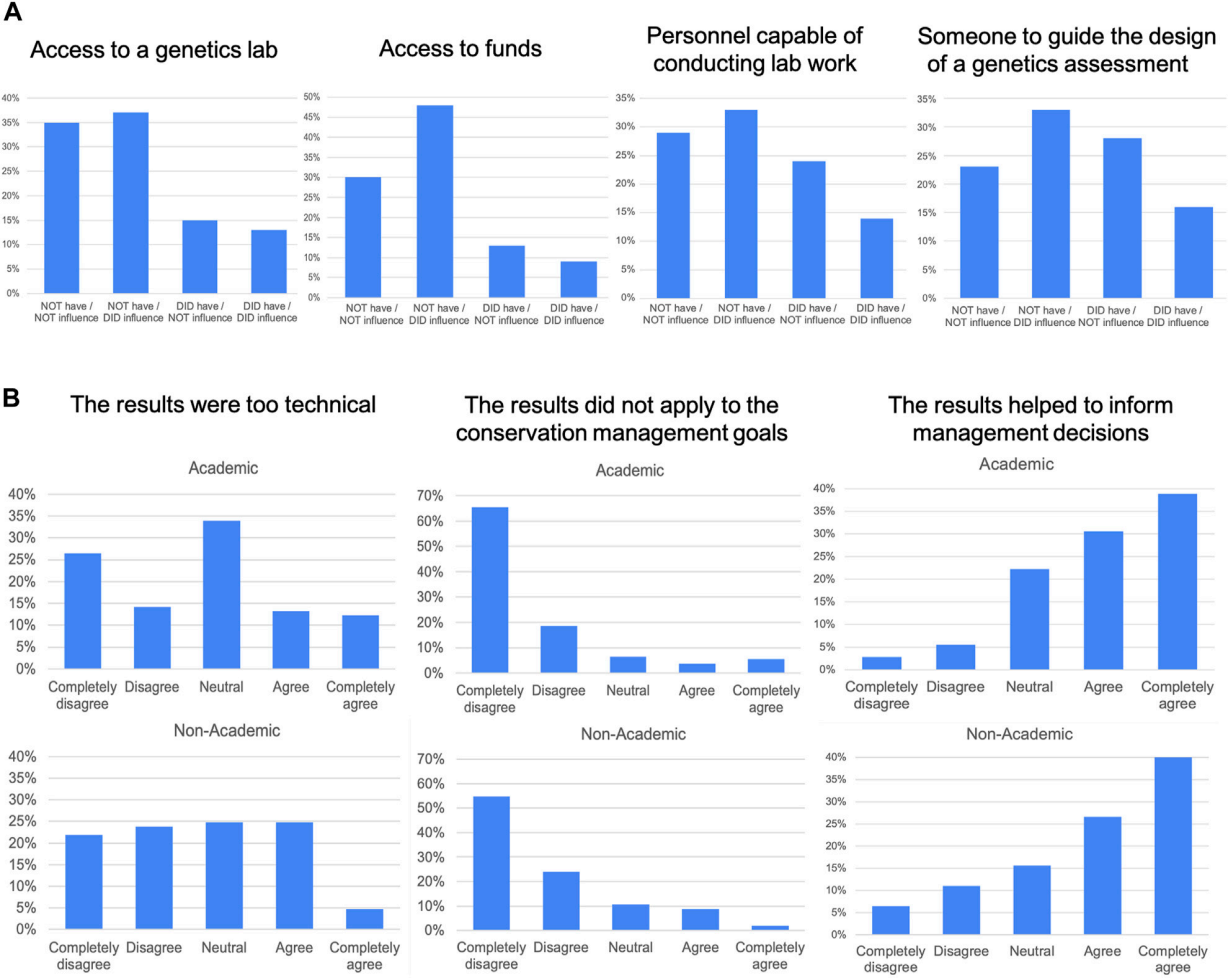
Main barriers and usefulness of genetic assessments for conservation managers in Latin America. **(A)** Main limitations of conservation managers that have not used genetics. Categories listed in the *x*-axis show whether respondents had or did not have a particular condition or situation, and whether that did or did not influence their decision to perform/use genetic assessments. We show the categories with the highest proportions for the “did NOT have/DID influence” situation. **(B)** Usefulness of genetic results for conservation managers. Categories listed in the *x*-axis correspond to respondent responses in a Likert scale from 1 (completely disagree) to 5 (completely agree).

For the total proportion of respondents from either an academic or non-academic background who had not performed or used a genetic assessment in their managed area/species (35%; Figure 2A), the main barriers or limiting factors that influenced their decision were, in order of importance: (i) Limited access to funding (48%), (ii) Limited access to a genetics lab (37%), (iii) Lack of help or guidance in the design of a genetics assessment (33%), (iv) Lack of qualified personnel for conducting lab work (33%), (v) Limited access to samples (24%), (vi) Lack of knowledge of questions that can be addressed (22%), (vii) Lack of confidence regarding the applicability of genetic results to management decisions (19%) and, (viii) Lack of qualified personnel for conducting fieldwork (18%) (Figure 3A).

Regarding the applicability of results of the genetic assessment, our findings show that: (i) 41% of academics and 46% of non-academics completely disagree and disagree with the statement “Results were too technical”, (ii) 82% of academics and 79% of non-academics completely disagree and disagree with the statement “Results did not apply to our conservation management goals”, and (iii) 71% of academics and 71% of non-academics completely agree and agree with the statement “Results helped inform management decisions” (Figure 3B).

### Co-development of conservation projects and data availability/sharing

To better describe the collaboration between academics and practitioners in Latin America, we asked about who had initially raised the questions addressed by genetic assessment. Our results show that for most academic respondents (58%), the research questions were raised by themselves or by someone from their group, compared to 25% of non-academics. In contrast, most non-academic respondents (46%) posed the questions in collaboration with partners from outside their organization, compared to 35% of academics. Only 7% of academics declared that the questions arose from a group outside their organization, compared to a much higher proportion of non-academics (29%).

Our results also show that 64% of academics and 65% of non-academics stated that the genetic assessments had been completed and the results were made available to managers by the time our survey ended (16 November 2021), while 11% of academics and 7% of non-academics stated that the assessments had finished but the results had not been made available to managers at this timepoint. Also, 25% of academics and 28% of non-academic respondents declared that the assessments were ongoing and thus the results were not available by the end of our survey.

### Partnerships between groups or organizations

To better understand barriers or challenges for initiating and/or maintaining partnerships between conservation genetic researchers and managers in Latin America, we asked managers how likely it was for them to contact different groups as potential partners to perform a genetic assessment. Our results show that respondents would contact, in order of importance: (i) an academic lab (Extremely likely and likely, 88%), (ii) another person/unit/branch in their organization (Extremely likely and likely, 68%), (iii) an NGO (Extremely likely and likely, 53%), (iv) a governmental organization (Extremely likely and likely 41%; low likelihood and not likely, 39%), (v) a private consulting company (Low likelihood and not likely, 55%), whilst (vi) 71% (Low likelihood and not likely) would not contact any group and would conduct it themselves.

Moreover, our results show that if managers were offered help to design and/or conduct a genetic assessment by an academic geneticist, 80% of them would be inclined to accept, 19% would maybe accept, and 1% would not accept. If a non-academic consulting service were available to help managers design and implement a genetic assessment, 51% of respondents declared that they or their organizations would maybe seek help from it, 32% would seek help, and 17% would not seek help.

Over half of the respondents (56%) declared that they had been contacted about performing a genetic assessment in their managed area/species and that the request came from the following groups, in order of prevalence: (i) external academic lab (44%), (ii) another person/unit in their organization (22%), (iii) external NGO (16%), (iv) external governmental organization (13%), and (v) private consulting company (5%). A considerable majority of such contacts came from within Latin America itself (70%), followed by North America (17%), Europe (8%), Other (2%), and no answer (3%).

### Gender gap

**FIGURE 4:**
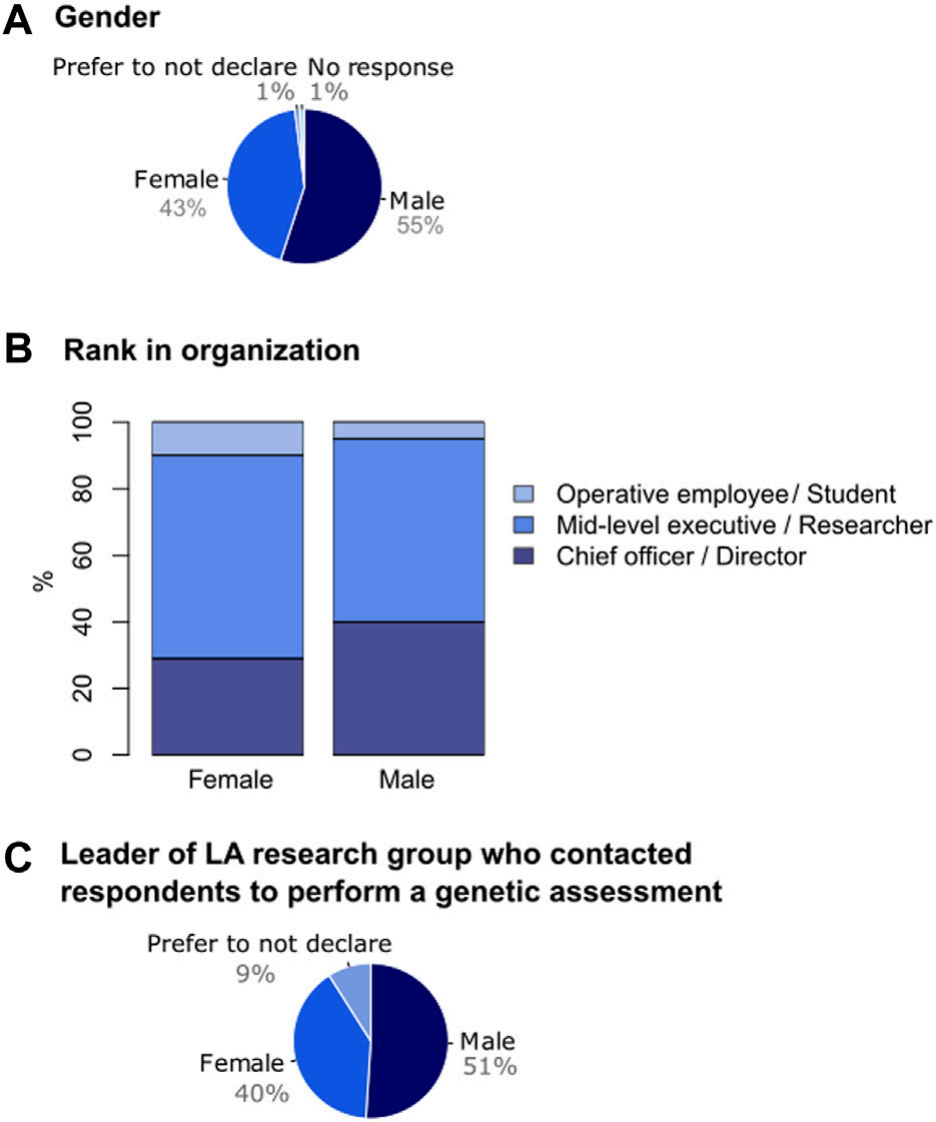
Gender gap in Latin American conservation managers. **(A)** Respondent’s gender.**(B)** Women *versus* men’s participation at different ranks in their organizations. **(C)** Women *versus* men’s participation as leaders of Latin American research groups who contacted respondents to perform a genetic assessment. LA = Latin America.

We were interested in assessing whether there was a gender gap between women and men in the area of conservation management in Latin America. Our results show that 63% of female managers occupied mid-level executive ranks (i.e., researcher) and 28% occupied chief officer or higher-ranking positions (i.e., director). In contrast, a higher proportion of male managers (37%) occupied chief officer or higher-ranking positions, with 56% occupying mid-level executive roles (i.e., researcher; Figure 4B).

Regarding respondents contacted by a Latin American research group to perform a genetic assessment, we asked about the gender of the group leader who had contacted them. The representation of women in such leadership roles (40%) was lower compared to men (51%), with 9% indicating uncertainty, preferring not to declare, or other (Figure 4B).

## Discussion

**FIGURE 5:**
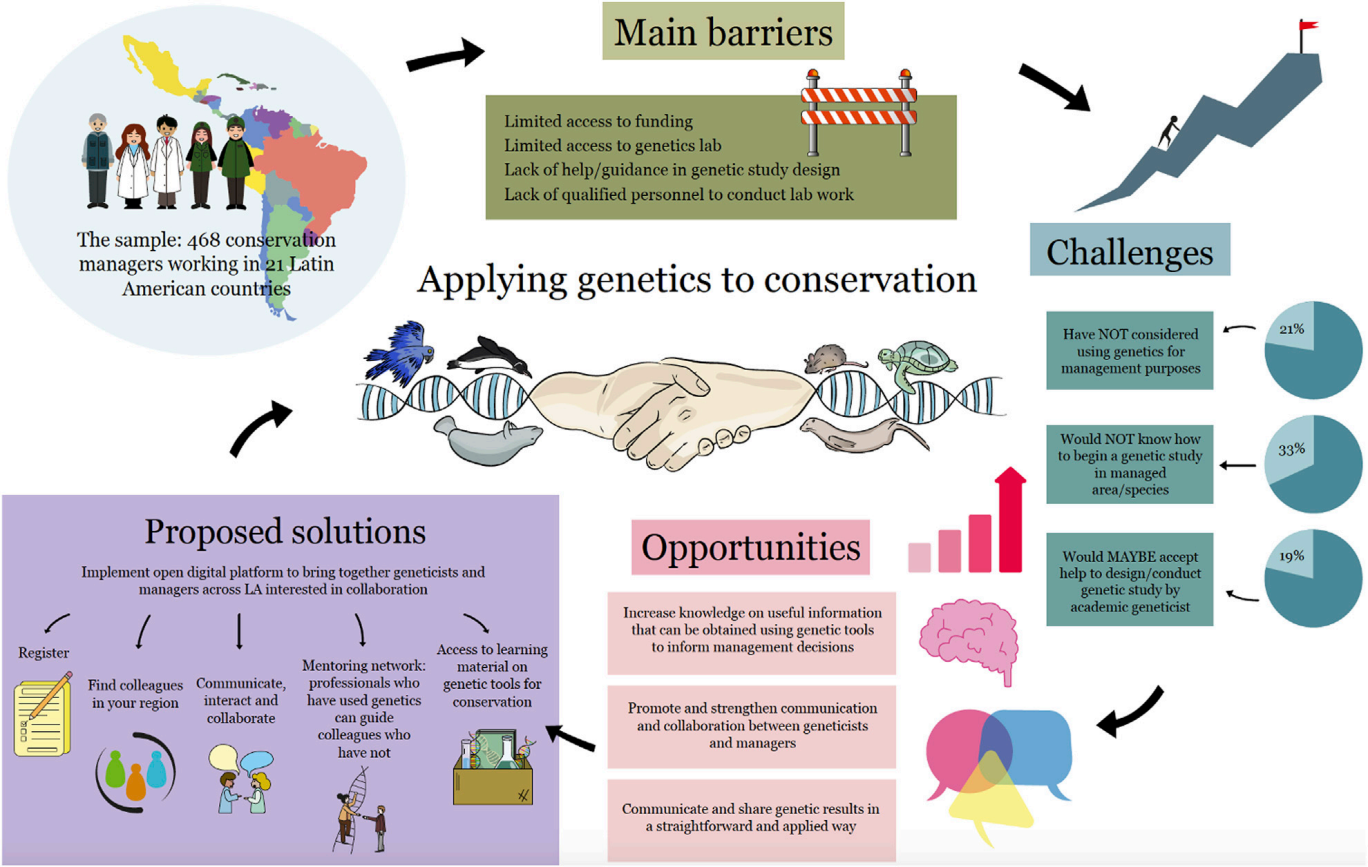
Conceptual framework for conservation practitioners in Latin America on how to bridge the conservation-genetics gap: Barriers, challenges, opportunities and proposed solutions (Illustration by Ahinoam Gonzalez Marchant).

This study provides a general diagnosis of the current situation of the conservation-genetics gap in Latin America. From the results obtained, we offer ideas and solutions for overcoming obstacles and advancing agendas that fit the needs and realities of this highly heterogeneous, biodiverse and challenging territory (Figure 5).

Overall sampling success was much higher through focal points (28.8%) compared to relying on organizations’ mailing lists (9.3%). Distribution via focal points also facilitated the reach of the survey to respondents in the majority of cases (81.8%). The personalized nature of invitations sent by focal points to target respondents likely fostered a greater sense of trust and relevance among potential participants, thereby enhancing motivation to respond. In contrast, the more impersonal approach of utilizing the entire mailing list of organizations may have resulted in lower levels of personal engagement and consequently reduced response rates. We advocate for the widespread adoption of focal points for conducting online surveys, as they may be more aware of the cultural aspects and local networks in their countries, leading to greater success in obtaining replies.

Respondents to our survey belonged to different organization types, such as academic or research institutions (44%), NGOs (29%) and governmental institutions (26%) (Figure 1B). The demographic composition in our study differed from that reported by Taft et al. (2020), in which a larger percentage of participants came from governmental institutions (50%), followed by academic institutions (24%) and NGOs (16%). Taft et al. (2020) described a trend among managers towards the use of genetics, as well as a higher incidence of respondents who had either conducted or employed genetic assessments compared to our study which described a lower proportion of genetic studies implemented by managers. Therefore, the differences observed between our study and that of Taft et al. (2020) could be due to geographical bias or differences in the types of institutions represented in each study. In general, developed nations allocate more public funds than developing nations for scientific research, including conservation science (Gonzalez-Brambila et al., 2016). Considering that the respondents to the survey in Taft et al. (2020) were primarily managers from governmental institutions based in the USA (50% of their sample), it is likely that they face fewer financial constraints to conduct conservation genetics assessments compared to Latin American managers. As a result, they may possess heightened awareness, training, and/or experience in this domain, compared to their Latin American counterparts.

In line with Torres-Florez et al. (2018), most of the respondents to our survey worked in the terrestrial realm (Figure 1D). Respondents declared that they had conducted genetic assessments mainly on animals, while plants were underrepresented in our sample. However, another meta-study reported that of all reviewed publications in conservation genetics (1980–2010), 46% were on plants (Oliveira-Miranda et al., 2013).

Replies to our survey show that most conservation managers in Latin America had already performed a genetic study or used genetic data in their managed area/species, and that half of them worked alone or in collaboration (Figures 2A, B). Encouragingly, we found that most conservation managers are not only aware of the usefulness of genetic assessments for informing and evaluating management actions (Figure 3B), but have also already used genetics and consider that they have the technical knowledge necessary to conduct or design a genetic assessment themselves, know how to apply or interpret published genetic data for achieving their conservation goals, and/or are able to actively find a collaborator. We found that a higher proportion of academic respondents had performed or used genetics to support their conservation goals compared to non-academics; this situation likely mirrors the greater access to updated research, training opportunities and collaborative networks within academic environments in contrast to non-academic ones. Notably, our findings are in contrast to other studies that have indicated a lack of awareness and knowledge regarding the usefulness of including genetic data into conservation actions (Cook et al., 2013; Klütsch and Laikre, 2021). This promising scenario highlights the potential benefits of promoting a mentoring network system within the conservation management community (see details in “Bridging the gap: recommendations for moving forward").

An important challenge in Latin America should be focused on managers who had not performed or used a genetic assessment, which comprise one-third of our sample (Figure 2A). These respondents indicated that the main barriers to implementing genetics are: limited access to funding, limited access to a genetics lab, lack of help or guidance in the design of a genetics assessment, and lack of a qualified personnel for conducting lab work (Figure 3A). This echoes other studies showing that most of the limitations for the effective implementation of genetics in conservation actions in Latin America are operational or financial, instead of being related to a lack of confidence in genetics (e.g., Ramirez et al., 2020).

In addition, and in line with surveys conducted worldwide (Haig et al., 2016; Taylor et al., 2017; Holderegger et al., 2019; Klütsch and Laikre, 2021), some respondents to our survey find genetic results too technical to interpret (25% of academics and 30% of non-academics) (Figure 3B). This is not surprising, given that conservation genetics as a research field requires the use of technical jargon and complex theoretical concepts, which necessarily need some basic background knowledge and training in order to be understood. Moreover, the concepts of conservation genetics are not commonly present in secondary education or postsecondary technical courses in Latin America (UNESCO, 2021).

We found that fewer non-academic respondents (25%) delineated the study questions addressed by genetic assessments, compared to a higher proportion of academics (58%) that did so, either individually or in collaboration. We recommend that efforts should be made to strengthen bonds between geneticists and managers so that they co-design genetic assessments from the onset, jointly defining the study questions and choosing the methodology to be applied, all in connection with the needs of the managers.

A high proportion of respondents to our survey, both academic (64%) and non-academic (65%), stated that when they worked in collaboration, the genetic results were readily available for them, in contrast to other studies that lament that the results gained from conservation genetics largely remain in the academic domain (Cook et al., 2013; Sandström et al., 2016; Taylor et al., 2017; Klütsch and Laikre, 2021). This result signals a positive scenario, given that the sharing of scientific results is the basis for transforming the information into effective management practices and to building trusting and successful long-term partnerships.

Results from our survey also indicate that managers in Latin America would more likely contact an academic laboratory rather than a private consulting company as a partner to perform a genetic assessment. Moreover, most managers would be inclined to accept help from an academic geneticist to design or implement a genetic assessment, preferring them over a private consulting service. As the private sector tends to be more expensive, these replies could highlight a lack of funding for managers in Latin America to pay for the services of private companies (consequently turning to academic collaborations which usually rely on the funding secured by the researchers) and/or mirror the high reputation of academic institutions which are deemed as being of excellence, having autonomy and being socially responsible (Alzyoud and Bani-Hani, 2015). This further emphasizes the need to promote increased funding for infrastructure and training of human resources and staff in academic and public institutions conducting theoretical and applied research in conservation genetics in Latin America.

A lower proportion of respondents were self-declared women (43%), compared to men (55%) (Figure 4A). Different scenarios, which are not mutually-exclusive but which are beyond the scope of this study, may explain this difference. Possible explanations are that they mirror a lower participation of women in conservation management in Latin America, or that females are indeed participating but did not answer the survey (presumably because they devote proportionally more time to housework and caregiving roles compared to men; Amarante et al., 2023; Mukhopadhyay, 2023). The difference may even reflect unintentional bias in the choice of target respondents contacted by our 32 focal points [even though nearly two-thirds (63%) of them were female] or under-representation in mailing lists in the collaborating organizations (SCB, ReGenec, LACA-SCB, Mesoamerican Society for Biology and Conservation). Among the self-declared women respondents, a smaller proportion (28%) occupy chief officer or higher rank positions (i.e., director) within their organizations, compared to men (37%) (Figure 4B), and a lower proportion of women were leaders of research groups performing genetic assessments (40%) compared to men (51%) (Figure 4C). Gender gaps and differences in science, technology, engineering and mathematics (STEM) have been widely described and studied worldwide (World Economic Forum, 2023), and also in Latin America (García-Holgado et al., 2020; Osorio-delvalle et al., 2020; Lappe et al., 2021; Braverman-Bronstein et al., 2023). Significant progress has been made during recent decades in Latin America to improve gender equality in STEM (García-Holgado et al., 2019; Mosquera and Rodríguez, 2023), and although some countries are doing well in advancing towards reducing the gender gap (World Economic Forum, 2023), there is still much room for improvement.

We also explored the potential occurrence and extension of parachute, or colonial, conservation science in Latin America by assessing the citizenship and residency of respondents. Our results show a promising scenario, indicating potential for local empowerment in conservation management given that the vast majority of respondents were either residents (92%) or nationals (92%), or both (87%), of the countries where their managed area/species is located. Moreover, our results show that local training and capacities for conducting conservation management for local biodiversity and genetic assessments are in place in the region (Figures 2A, B and see Results). Our findings are in line with those reported in a review on plant conservation genetics (Oliveira-Miranda et al., 2013) which described an increased participation of Latin Americans as first authors (from 46% in the 1990s to 71% in 2006–2010), and an increased number of publications only authored by Latin Americans. Oliveira-Miranda et al. (2013) also found that the number of studies in plant conservation genetics in Latin America with only foreign co-authors was higher in countries between Mexico and Panama, while those including only local co-authors was higher from Colombia to Brazil and Bolivia.

This study was designed to prioritize adequate geographic distribution of the sampling across Latin American countries, being one of the first studies to conduct a broad survey across the entire region to describe the gap in conservation genetics (Ceballos et al., 2009; Taylor et al., 2017; Fabian et al., 2019; Taft et al., 2020). The geographic representation of our results is wider and larger than that of previous studies (Rojas Bonzi et al., 2018; Taft et al., 2020). It is worth noting that the results of our survey cannot be taken as a description of the complex nature of the entire population of Latin America conservation managers, because our survey distribution was not random. We can only describe and discuss our results based on our sample, considering the respondents’ information and acknowledging potential biases. Despite our efforts to ensure a diverse and balanced sample, targeting respondents from both academic and non-academic backgrounds, as well as from various organizational affiliations, we recognize potential limitations. Focal points might have unintentionally contacted more people who share a similar enthusiasm for using genetic data in conservation efforts, even though a clear effort to avoid this bias was established from the onset. Therefore, as in Taft et al. (2020), our sample may have a higher representation of managers who were already aware, interested in or trained in genetics compared to the real proportion they may represent across Latin America. In addition, the use of mailing lists from certain organizations may have also induced sampling bias, for example, that of the Latin American Conservation Genetics Network (ReGenec), even though its specific representation in our sample was much lower (6.8%) compared to focal points (81.8%). Our sample might have been unbalanced in other aspects as well, such as the habitat or realm where respondents work (mainly terrestrial, 70%; Figure 1D), or the taxonomic groups they study (mainly animals, 71%). Nevertheless, we believe that our sample reflects the diversity of conservation managers present in Latin America regarding the type of organizations, role of respondents and including both *in-situ* and *ex-situ* managers (see Results; Figure 1C). Social desirability bias (i.e., the tendency to align responses with what is perceived to be socially acceptable or politically correct) may limit the interpretation of our findings, as it occurs in many other qualitative research studies using surveys (Bergen and Labonté, 2020).

When interpreting and discussing our results, we acknowledge the high heterogeneity of local realities and working conditions that coexist across Latin American countries regarding research and scientific development, infrastructure capabilities, specialized training, and access to published scientific information (UNESCO, 2021). Even though Latin American countries share similar socioeconomic histories and scenarios (Ceballos et al., 2009; Torres-Florez et al., 2018), there are significant differences across the region. For example, Brazil, Argentina, Mexico and Chile are more advanced in the integration of genetics into conservation management, compared to other Latin American countries (Oliveira-Miranda et al., 2013; Torres-Florez et al., 2018), due to a myriad of factors such as differences in funding opportunities and/or legislation, barriers and the possible ways genetics can be applied to conservation (e.g., Torres-Florez et al., 2018; Taft et al., 2020). In the context of this highly heterogeneous region, the conservation management of species with trans-national ranges is an enormous challenge. The marked diversity we observe in this regard across Latin America is probably much less pronounced across the Global North (UNESCO, 2021). Thus, despite our large sampling size (n = 468 respondents), the overall results and conclusions of our study may mask differences between Latin American countries, because it was not designed to address each one in detail. Future studies should tackle differences across the Latin American region using a larger sample size per country to allow a more in-depth assessment.

### Bridging the gap: recommendations for moving forward

Strategies for practitioners to incorporate genetics in their conservation management practices and to bridge the conservation-genetics gap between managers and researchers should be specifically designed for Latin America. Based on our results, we provide some recommendations, mechanisms and guiding examples for moving towards bridging the conservation-genetics gap in the region:

(1) TRAINING: Promote and expand technical and theoretical training opportunities for managers, practitioners and decision makers in conservation genetics issues.
• Develop and disseminate short courses and diplomas in conservation genetics specifically for managers, conservation practitioners and decision makers.
• Train managers and decision makers in the use of the jargon associated with conservation genetics, to help them interpret results in technical reports and scientific articles.
• Strengthen teaching of conservation genetics in secondary schools, technical secondary courses, biological sciences degrees and graduate courses related to biodiversity conservation.
  - Organizations, associations or societies offer short courses to strengthen general knowledge (e.g., The Paraguayan Mastozoology Association offers introductory Conservation Genetics courses during congresses).
  - Conservation Genetics courses offered by university postgraduate diplomas (e.g., Specialization in Conservation Biology, Universidad Nacional de Misiones, Argentina).
  - Since 2005, ReGenec (The Conservation Genetics Network) annually offers a Conservation Genetics course for Latin American graduate students (https://regenec.org).
• Provide opportunities and funding for scholarships, exchange programs and short stays to visit conservation genetics laboratories within Latin America.
  - Funds available for short stays: In Paraguay, CONACYT (https://www.conacyt.gov.py/conacyt-lanza-oportunidad-para-realizar-estancias-investigacion-corta-duracion); in Brazil, Move La America Program (https://www.gov.br/capes/pt-br/acesso-a-informacao/acoes-e-programas/bolsas/bolsas-e-auxilios-internacionais/encontre-aqui/paises/multinacional/programa-move-la-america).
(2) COLLABORATION: Foster constant interaction and collaboration between academic researchers, managers and decision makers to conduct conservation projects and integrate genetics into wider conservation strategies.
• As part of their social responsibility and public role, academic and research institutions should strengthen regular communication, engagement and collaboration with non-academic conservation management organizations.
• Foster close communication between scientists and managers with local experience in the field, from the onset of conservation projects onwards and throughout all stages of conservation projects, facilitating communication of needs, helping frame and co-design research questions, promoting direct feedback on the feasibility of implementation of conservation recommendations and actions, and strengthening trustful long-term partnerships.
• Promote multi, inter and transdisciplinary approaches, cooperation, active feedback between disciplines and constant dialogue among key actors and stakeholders.
  - Hold regular meetings between researchers/geneticists and managers/decision makers at the local level to present and discuss research results.
  - Organize regular intersectoral roundtables and panels for technical discussion among researchers in genetics and decision makers who need genetic data to improve evidence-based decisions for biodiversity conservation and management.
  - Open instances of regular dialogue between researchers and governmental agencies in charge of environmental resources to discuss how genetic information can help delineate and achieve wider conservation and sustainable development strategies.
• Facilitate access and interpretation of research results for managers, practitioners and decision-makers.
  - Encourage geneticists and researchers to write reports in a simple, straightforward and applied way, and share them with managers, practitioners and decision-makers.
  - Train researchers in scientific communication skills to help them disseminate results in a simpler way.
  - Promote researchers to publish research articles in Spanish, Portuguese or the local language to make them more widely accessible for managers, practitioners and decision makers for immediate applicability in local conservation practices (e.g., if published in an English-written journal, include a version in the local language in Supplementary Material or Appendices) (See Supplementary Appendix SIV, SV).
• Implement an open digital platform to bring together geneticists and managers across Latin America interested in collaboration: register, find colleagues in the region, communicate and interact, join a mentoring network system where professionals who have already used genetics could guide, support and share knowledge with their colleagues who have not, and access learning material on genetics tools for conservation (Figure 5).
• Increase incentives for academics to consider collaborating with managers and decision makers to jointly implement better conservation management strategies and practices.
  - National agencies and their funding programs (e.g., CONACYT (Paraguay), CONICET (Argentina), ANID (Chile), CNPq (Brazil)), universities and research institutions should recognize and consider academic-manager collaborations when scoring proposals for funding and applicants during recruitment and career progression.
  - Employers should encourage and motivate researchers to engage in activities promoting interaction with conservation managers, such as meetings in which decision makers and scientists could directly discuss their needs and find common ground for collaboration.
  - Employers should value and encourage geneticists and researchers to write reports in a simple, straightforward and applied way and in the local language.
(3) FUNDING: Promote increased funding for infrastructure and project implementation in academic and public institutions conducting conservation genetics in Latin America.
• International consortiums and intergovernmental programs sponsored by developed countries should provide opportunities to overcome the lower financial capacities of public and academic institutions in Latin America (e.g., Genotropics; www.genotropics.org).
• Urge the private sector to increase investment in conservation genetics.
• Promote population genetics research and capacity building projects in transboundary protected areas to evaluate the scope of conservation strategies for threatened species.
(4) WOMEN'S PARTICIPATION: Foster and increase women’s participation in conservation management and decision making.
• Provide specific opportunities for women to move to higher ranks in their organizations and obtain leadership positions.
• Promote training and funding specifically for women in conservation.
  - The Organization for Women in Science for the Developing World (OWSD) has funding and other opportunities for members.
  - Woman in Nature (WiNN) has a Mentorship Program and leadership training to empower women in conservation and management and help them thrive in their careers.
(5) POLICY: Integrate the use of genetics into regional conservation policies and promote policies of fairness, equity and diversity in research collaborations.
• Integrate conservation genetics issues into wider discussions on biodiversity conservation strategies and policies, by organizing specific fora with decision makers, from protected area managers to conservation policymakers, to mainstream the application of genetics to conservation.
  - Update the countries’ National Biodiversity Strategies and Action Plans (NBSAPs) to meet the targets of the Kumming-Montreal framework of the Convention on Biological Diversity (CBD).
  - Define national biodiversity conservation strategies.
• Disseminate, promote and enforce policies of fairness, equity and diversity in research collaborations in both scientific and professional contexts.
  - Create and enforce clear cooperation agreements and guidelines between institutions and countries to facilitate sample transport and the sharing of genetics results in collaborations for conservation purposes.
  - Disseminate and enforce clear regulations on permits for conducting research and accessing genetic resources, especially in the context of high biodiversity and high threats in the Latin American region (e.g., the Cartagena Protocol on Biosafety to the Convention on Biological Diversity (https://bch.cbd.int/protocol), the Nagoya Protocol on Access and Benefit-sharing (https://www.cbd.int/abs), and different in-country regulations.
• Researchers and managers should promote and integrate perspectives on gender, diversity and inclusion, and avoid discrimination and bias in the implementation of conservation genetics studies.
• Promote collective leadership and partnerships between institutions in the Global North and the Global South towards respectful and fair biodiversity conservation practices (Ruelas Inzunza et al., 2023; Soares et al., 2023).
  - Contact Latin Americans residing in the Global North and the Global South to help forge international collaborations and partnerships.

## Data Availability

The raw data supporting the conclusion of this article will be made available by the authors, without undue reservation.
