# Supplementary material for: Understanding the conservation-genetics gap in Latin America: challenges and opportunities to integrate genetics into conservation practices
Source: Front Genet. 2024 Jul 8;15:1425531. doi: 10.3389/fgene.2024.1425531 (PMC11261212; doi:10.3389/fgene.2024.1425531)
Supplement: Supplementary file 1 [file DataSheet4.docx]

**Supplementary Material**

**Appendix IV: Spanish version of the manuscript**

**Comprendiendo la brecha entre conservación y genética en América Latina:
Desafíos y oportunidades para integrar la genética en las prácticas de conservación**

Constanza Napolitano*^1,2,3^, Cristhian Clavijo^4^, Viviana Rojas-Bonzi^5,6^, Carolina I Miño^7^, José F González-Maya^8^, Nadia Bou^9^, Alan Giraldo^10^, Angela Martino^11^, Cristina Yumi Miyaki^12^, Luis F Aguirre^13^, Andrea Cosacov^14^, Yoamel Milián-García^15^, Laura Prosdocimi^16^, O Eric Ramírez-Bravo^17^, Luis Antonio Tovar^18^, Ximena Velez-Zuazo^19^, Mercedes Barrios^20^, Bernal Herrera-Fernández^21^, María G Montiel-Villalobos^22^, María A Oliveira-Miranda^23^, Monique Pool^24^, Alonso Santos-Murgas^25^, M Claudia Segovia-Salcedo^26^, Felipe Cecchi^27^, Armando J Dans^28^, Nelanie Dilchand^29^, Sergio MQ Lima^30^, María Caridad Novas^31^, Karla Pelz-Serrano^32^, Nina Pougy^33^, Iris Rodríguez^34^, Liesbeth van der Meer^35^, Galo Zapata-Ríos^36^.

^1^Departamento de Ciencias Biológicas y Biodiversidad, Universidad de Los Lagos, Osorno, Chile.
^2^Institute of Ecology and Biodiversity, Concepción, Chile.
^3^Cape Horn International Center, Puerto Williams, Chile.
^4^Vida Silvestre Uruguay, Montevideo, Uruguay.
^5^Instituto de Investigación Biológica del Paraguay, Asuncion, Paraguay.
^6^Wildlife Ecology and Conservation Department, University of Florida, Gainesville, USA.
^7^Laboratorio de Genética Evolutiva - LGE, Instituto de Biología Subtropical - IBS, Consejo Nacional de Investigaciones Científicas y Técnicas - CONICET, Universidad Nacional de Misiones (UNaM), Posadas, Misiones, Argentina.
^8^Área de Biología de la Conservación, Departamento de Ciencias Ambientales, DCBS, Universidad Autónoma Metropolitana Unidad Lerma, Lerma de Villada, México & ProCAT Colombia/Costa Rica, Bogotá, Colombia.
^9^Departamento de Biodiversidad y Genética, Instituto de Investigaciones Biológicas Clemente Estable, Montevideo, Uruguay.
^10^Grupo de Investigación en Ecología Animal, Departamento de Biología, Facultad de Ciencias Naturales y Exactas, Universidad del Valle, Cali, Colombia.
^11^Centro de Investigaciones en Ecología y Zonas Aridas, Universidad Nacional Experimental Francisco de Miranda, Coro, Falcón, Venezuela.
^12^Departamento de Genética e Biologia Evolutiva, Instituto de Biociências, Universidade de São Paulo, São Paulo, Brazil.
^13^Centro de Biodiversidad y Genética, Universidad Mayor de San Simón, Cochabamba, Bolivia.
^14^Laboratorio de Ecología Evolutiva y Biología Floral, Instituto Multidisciplinario de Biología Vegetal, CONICET-Universidad Nacional de Córdoba, Córdoba, Argentina.
^15^Department of Integrative Biology, University of Guelph, Guelph, Canada.
^16^Laboratorio de Ecología, Comportamiento y Mamíferos Marinos (LECyMM), Museo Argentino de Ciencias Naturales (MACN-CONICET), Buenos Aires, Argentina.
^17^Centro de Agroecología, Instituto de Ciencias, Benemérita Universidad Autónoma de Puebla, Eco campus Valsequillo, San Pedro Zacachimalpa, México.
^18^Facultad de Ciencias Forestales, Universidad Nacional Agraria La Molina, Lima, Perú.
^19^Smithsonian National Zoological Park and Conservation Biology Institute, Washington DC, USA.
^20^Centro de Datos para la Conservación, Centro de Estudios Conservacionistas, Universidad de San Carlos de Guatemala, Ciudad de Guatemala, Guatemala.
^21^Instituto Internacional de Conservación y Manejo de Vida Silvestre (ICOMVIS), Universidad Nacional, Heredia, Costa Rica.
^22^Red Latinoamericana de Genética para la Conservación (ReGeneC), La Paz, BCS, México.
^23^Red Latinoamericana de Genética para la Conservación (ReGeneC), Caracas, Venezuela.
^24^Green Heritage Fund Suriname, Paramaribo, Suriname.
^25^Departamento de Zoología, Facultad de Ciencias Naturales Exactas y Tecnología, Universidad de Panamá, Ciudad de Panamá. Estación Científica Coiba AIP, Ciudad del Saber, Panamá.
^26^Departamento de Ciencias de la Vida y la Agricultura, Universidad de las Fuerzas Armadas -ESPE, Sangolquí, Ecuador.
^27^Grupo Antropología de la Conservación, Universidad de Los Lagos, Osorno, Chile.
^28^Departamento de Ciencias Ambientales y Producción Sostenible, Universidad URACCAN, Bluefields, Nicaragua.
^29^Aquatic and Terrestrial Pioneers Consulting Services, Georgetown, Guyana.
^30^Departamento de Botânica e Zoologia, Universidade Federal do Rio Grande do Norte, Natal, Brazil.
^31^División de Conservación, Departamento de Botánica, Jardín Botánico Nacional Dr. Rafael María Moscoso, Santo Domingo, República Dominicana.
^32^Departamento de Ciencias Ambientales, División de Ciencias Biológicas y de la Salud, Universidad Autónoma Metropolitana Unidad Lerma, Lerma, México.
^33^Departamento de Desenvolvimento Científico, Museu do Amanhã, Instituto de Desenvolvimento e Gestão - IDG, Rio de Janeiro, Brazil.
^34^Escuela de Biología, Universidad Nacional Autónoma de Honduras, Tegucigalpa, Honduras.
^35^Oceana Chile, Santiago, Chile.
^36^Wildlife Conservation Society - Ecuador Program, Quito, Ecuador.

*Autora correspondiente: constanza.napolitano@ulagos.cl

**Resumen**

La integración de los datos genéticos en las decisiones de manejo para la conservación es una tarea difícil que requiere una estrecha colaboración entre investigadores y gestores. La conservación en América Latina tiene crucial relevancia a nivel global, dados los altos niveles de biodiversidad y la presencia de puntos críticos de biodiversidad en esta región. Realizamos una encuesta en América Latina para identificar obstáculos y oportunidades entre investigadores dedicados a la genética y gestores de conservación. Nuestro objetivo es comprender mejor los puntos de vista de los gestores de la conservación y cómo la investigación genética podría ayudar a los profesionales de la conservación a alcanzar sus objetivos mediante la implementación de estudios genéticos que informen eficazmente las prácticas de conservación. A tal fin, distribuimos una encuesta en línea a través de cuatro organizaciones regionales colaboradoras y 32 puntos focales con sede en 20 países latinoamericanos. Los encuestados eran gestores de conservación de especies o áreas de América Latina. Recogimos un total de 468 cuestionarios contestados, procedentes de 21 países latinoamericanos. La mayoría de los encuestados (44%) pertenecen a instituciones académicas o de investigación, mientras que los no académicos proceden principalmente de instituciones no gubernamentales (30%) y organismos gubernamentales (25%). La mayoría de los encuestados (65%) han realizado o utilizado estudios genéticos en su área o especie gestionada, ya sea solos, en colaboración, contratando a alguien más, o utilizando resultados publicados. Para la mayoría de encuestados en este grupo, los resultados genéticos fueron considerados relevantes para sus objetivos de manejo para la conservación y contribuyeron a informar las decisiones de manejo. Los encuestados que no habían realizado o utilizado estudios genéticos (35%) pertenecen principalmente al grupo no académico, y entre estos, los principales obstáculos mencionados fueron el acceso limitado a financiamiento, a instalaciones de laboratorio genético, y a personal capacitado para diseñar estudios y realizar trabajos de laboratorio. A partir de los hallazgos, describimos la situación actual y proporcionamos un diagnóstico general de la brecha entre conservación y genética en América Latina. Analizamos además la brecha de género, el desarrollo conjunto de preguntas y proyectos de conservación por parte de académicos y profesionales, y la nacionalidad y residencia de los gestores de la conservación latinoamericanos en relación con los países en los que trabajan. Discutimos asimismo las oportunidades de creación conjunta de preguntas de investigación y el codesarrollo de estudios basados en las necesidades de los profesionales de la conservación. Finalmente, ofrecemos recomendaciones para superar las barreras que impiden integrar la información genética en las acciones de conservación, y proponemos agendas ajustadas a las necesidades y realidades de la altamente heterogénea, biodiversa y desafiante región latinoamericana.

**Palabras clave:** prácticas de manejo para conservación, gestores de la conservación, genetistas, colaboraciones, conocimientos locales, especies amenazadas, áreas importantes para la conservación.

**Introducción**

Para responder a la actual crisis de biodiversidad provocada por el cambio global inducido por el ser humano (Ceballos et al., 2020), es esencial integrar y traducir la evidencia científica en marcos sociopolíticos, procesos clave de toma de decisiones, y políticas públicas (Díaz et al., 2019). Esta misión urgente exige una visión amplia e interdisciplinar por parte de investigadores, gestores de la conservación, responsables de la toma de decisiones, políticos, legisladores y todas las partes interesadas en general, que deben colaborar en la búsqueda de objetivos e indicadores de investigación comunes y en la aplicación de medidas para preservar la biodiversidad (Ferreira & Klütsch, 2021; Kershaw et al., 2022; Willi et al., 2022).

Entre los componentes de la biodiversidad, la diversidad genética desempeña un papel crucial en la resiliencia de los ecosistemas y en la adaptación y supervivencia de las especies (Hoban, et al., 2021a; Laikre et al., 2020; Taft et al., 2020; Díaz et al., 2019; Taylor et al., 2017). Los datos genéticos proporcionan información única sobre el estado de conservación y las tendencias de las especies y poblaciones (Willoughby et al. 2015; Pierson et al. 2016; Torres-Florez et al., 2018; Garner et al., 2020). Sin embargo, a pesar de su reconocida importancia, la incorporación de la información genética a las estrategias y políticas de gestión para la conservación sigue siendo un desafío mundial (Laikre, 2010; Laikre et al. 2010, 2016; Shafer et al 2015; Cook & Sgrò, 2017, 2018; Sandström et al., 2019; Taft et al., 2020; Hoban et al 2020, 2021b; Galetti, 2023), lo que contribuye a lo que se conoce como la "brecha entre la conservación y la genética" (Taylor et al., 2017).

Los estudios que han analizado la brecha entre la conservación y la genética han identificado cuatro barreras principales para incluir los datos genéticos en la gestión para la conservación: i) la falta de un mecanismo eficiente para la transferencia de conocimientos entre académicos y partes interesadas (Hoban et al., 2013; Taylor et al., 2017; Britt et al., 2018; Taft et al., 2020; Klütsch & Laikre, 2021), ii) la falta de formación formal entre los gestores para interpretar y evaluar los datos genéticos (Cook & Sgrò, 2017, 2018; Haig et al., 2016; Taft et al., 2020; Taylor et al., 2017), iii) el costo percibido por los gestores y el financiamiento necesario para realizar estudios genéticos (Klütsch & Laikre, 2021; Taylor et al., 2017) y iv) las estructuras o restricciones burocráticas de las instituciones gubernamentales pertinentes (Rojas Bonzi et al., 2018; Klütsch & Laikre, 2021). Estas barreras pueden verse exacerbadas en regiones extensas, muy diversas y culturalmente heterogéneas como América Latina. Esta región geográfica alberga una proporción importante de la biodiversidad total de la Tierra (≈40%; UNEP-WCMC 2016), incluye varios puntos críticos de biodiversidad (Myers et al., 2000, Mittermeier et al., 2011), y se enfrenta a una grave y creciente degradación del hábitat y a un rápido declive de las poblaciones silvestres (Josse & Fernández, 2021; Myers et al., 2000; Rodríguez-Clark et al., 2015; PNUMA-WCMC 2016).

La información genética se ha utilizado cada vez más para la conservación en América Latina (Torres-Florez et al., 2018; Moraes et al., 2023), con liderazgo de Brasil, México y Chile en cuanto al número de estudios publicados (Rodríguez-Clark et al., 2015). Estos estudios se centran principalmente en especies terrestres (Oliveira-Miranda et al., 2013; Torres-Florez et al., 2018), en temáticas de genética de poblaciones y diversidad genética, y en la evaluación de las consecuencias genéticas de la sobreexplotación y la pérdida de hábitat (Torres-Florez et al., 2018). Si bien varios de estos artículos han incluido recomendaciones específicas para acciones de conservación y gestión, el grado en que estas sugerencias u otros hallazgos genéticos se han integrado efectivamente en las estrategias de conservación, las acciones de manejo y/o las políticas formales en toda América Latina sigue sin estar claro (Torres-Florez et al., 2018), particularmente en países con una producción científica limitada. La comprensión de cómo la genética se integra en las estrategias de conservación es fundamental para desentrañar los impulsores y factores que perpetúan la brecha entre la conservación y la genética a través de América Latina.

En el presente estudio, nos propusimos específicamente caracterizar la brecha entre la conservación y la genética en América Latina, investigando exhaustivamente la relación entre la investigación genética y el manejo para la conservación en la región. Buscamos identificar y comprender los inconvenientes a superar, así como los desafíos y oportunidades que enfrenta la comunidad conservacionista latinoamericana para integrar la genética en la práctica de la conservación. Basamos nuestro estudio en el realizado por Taft et al. (2020), quienes exploraron las barreras que impiden el uso de datos genéticos para la práctica y la política de conservación mediante encuestas a profesionales de la conservación de diversas instituciones académicas y gubernamentales (n=50). Sin embargo, sus conclusiones proceden predominantemente de una muestra geográficamente sesgada, principalmente de los Estados Unidos de América. Por lo tanto, las ideas derivadas de Taft et al. (2020) no son directamente transferibles y no pueden generalizarse para evaluar el uso eficaz de la información genética en las prácticas de gestión para la conservación en la región altamente heterogénea de América Latina.

Con base en estas consideraciones, nuestros objetivos fueron: i) identificar las brechas y oportunidades de colaboración entre genetistas y profesionales con competencia en el campo de la conservación, con el fin de implementar estudios o evaluaciones genéticas (a nivel de población, especie y ecosistema) que puedan informar la gestión o manejo para la conservación en América Latina, y ii) comprender cómo los investigadores y los profesionales de la conservación pueden colaborar eficazmente y participar en asociaciones fructíferas para lograr objetivos comunes de conservación en la región. Evaluamos las principales barreras y discutimos recomendaciones y perspectivas para la cocreación y codesarrollo de estudios específicamente adaptados a las necesidades de los profesionales de la conservación, las partes interesadas y/o las comunidades locales, para así fomentar la integración efectiva de la información genética en las acciones de conservación en la región Latinoamericana rica en biodiversidad.

**Métodos**

**Área de estudio**

Se pretendió evaluar 20 países de las Américas Australes y Neotropicales (ANA), que comprenden México, Centroamérica, el Caribe (incluyendo Guyana, Surinam, Guayana Francesa y las islas del Caribe) y Sudamérica. Esta área se conoce políticamente como América Latina, cuyos países poseen historias y escenarios socioeconómicos similares o comunes (Ceballos et al., 2009, Torres-Florez et al., 2018).

**Distribución y aplicación de la encuesta**

Para seleccionar a nuestros encuestados objetivo utilizamos el muestreo intencional, una técnica implementada para identificar y seleccionar individuos o grupos de individuos especialmente conocedores o experimentados en un fenómeno de interés (Cresswell & Plano, 2011). Para lograr un tamaño de muestra representativo y una distribución homogénea del esfuerzo de muestreo en toda América Latina, trabajamos con 32 puntos focales (63% mujeres; 38% hombres) distribuidos en cada uno de los 20 países seleccionados. Definimos un punto focal como una persona que trabaja en conservación en un país latinoamericano, y cuya función fue identificar y contactar a otros encuestados potenciales, distribuir las encuestas, y realizar seguimientos. El número de puntos focales por país (1-3) se estableció en función de la población total del país y se estandarizó por el número de investigadores por país (expresado por millón de habitantes) a partir de los datos del Instituto de Estadística de la UNESCO (IEU) en el Banco de Datos del Banco Mundial (UNESCO, 2021). Además del muestreo directo a través de puntos focales, también distribuimos la encuesta utilizando listas de correo grupales a través de las siguientes organizaciones colaboradoras: El Capítulo del Cono Sur de Sudamérica de la Sociedad para la Biología de la Conservación (SCB), la Red Latinoamericana de Genética para la Conservación (ReGeneC), la Sección de América Latina y el Caribe de la SCB (LACA-SCB) y la Sociedad Mesoamericana de Biología y Conservación.

**Encuestados objetivo**

Los encuestados objetivo fueron gestores de la conservación en América Latina, implicados directa y recientemente (2017-2021) en la conservación de una especie o área, ya sea mediante la planificación de estrategias de conservación (por ejemplo, planes de acción para especies), la supervisión del manejo, el monitoreo de especies o la evaluación de los resultados de dichas prácticas. Los científicos que no participaban directamente en manejo para la conservación, o sin experiencia práctica en conservación, no fueron seleccionados como encuestados.

**Diseño de la encuesta**

Adaptamos la encuesta implementada por Taft et al. (2020) dado nuestro enfoque en gestores y profesionales latinoamericanos. Así, incluimos preguntas adicionales basadas en aspectos específicos que queríamos abordar en un contexto regional, como la presencia y práctica de la ciencia ‘colonial’ o ‘paracaidista’ (*colonial/parachute science*) (Haelewaters et al., 2021; Liboiron, 2021; Asase et al., 2022; de Vos & Swartz, 2022, Horn et al., 2022) y la brecha de género. La encuesta se tradujo a los cuatro idiomas oficiales más habladas en la región geográfica seleccionada: español, portugués, inglés y neerlandés. Utilizamos Google Forms (Google 2021) para realizar la encuesta. Recogimos las respuestas durante un periodo de 3 meses (del 15 de agosto al 16 de noviembre de 2021). La encuesta, de 15 minutos de duración (Apéndice I, Material Suplementario), no contenía preguntas obligatorias y todas las respuestas se registraron como anónimas. Las 35 preguntas se agruparon en cuatro secciones: (i) Datos demográficos del encuestado, (ii) Área/especie gestionada, (iii) Tipo de estudio genético realizado y (iv) Asociaciones con otros grupos de investigación y/o profesionales de la conservación. Se utilizaron diferentes formatos de pregunta: opción múltiple (exclusiva/no exclusiva; n=25), escala de Likert de cinco puntos (Likert, 1932; n=5) y preguntas abiertas (n=5). En la primera sección se indagaron nacionalidad, género, edad, lugar de trabajo, tipo de institución laboral y función del encuestado dentro de la institución/organización. En la segunda sección se recopiló información sobre la ubicación geográfica del área u objeto de estudio, el ámbito de las áreas/especies gestionadas (marinas, de agua dulce, o terrestres) y los intereses específicos de investigación y/o monitoreo en relación con el área/especie gestionada. En la tercera sección se indagó sobre la utilidad de los distintos tipos de evaluaciones genéticas, el interés en realizarlas, la información sobre aquellas ya realizadas, y los posibles obstáculos para llevarlas a cabo o utilizarlas. En la cuarta sección examinamos si el encuestado tuvo que pedir ayuda a expertos y si confian en ellos. Además, evaluamos la eficacia de los métodos de distribución y muestreo de la encuesta incluyendo una pregunta sobre cómo había llegado la encuesta al encuestado. Registramos el esfuerzo de muestreo (es decir, el número de invitaciones enviadas) y medimos el éxito del muestreo calculando porcentajes de encuestas contestadas/esfuerzo. Comparamos el éxito de la distribución de encuestas entre los puntos focales y las listas de correo de las organizaciones para explorar la eficacia de ambas estrategias de distribución de encuestas.

Se utilizó estadística descriptiva para resumir los resultados. La encuesta incluyó una declaración de Consentimiento Informado y fue revisada y aprobada por el Comité de Ética del Instituto de Ecología y Biodiversidad (IEB, Chile) (Certificado de aprobación emitido el 12 de agosto de 2021).

**Resultados**

**Distribución de la encuesta y éxito del muestreo**

Se enviaron 2.196 invitaciones a potenciales participantes en la encuesta, lo que representa el esfuerzo total de muestreo. El éxito del muestreo varió en función del método de distribución de la encuesta: las invitaciones enviadas a través de los puntos focales dieron lugar a 383 respuestas (de 1.344 invitaciones enviadas, con una tasa de respuesta del 28,5%, lo que representa el 81,8% de las respuestas), mientras que las enviadas a través de listas de correo de organizaciones colaboradoras dieron lugar a 79 respuestas (de 852 invitaciones, con una tasa de respuesta del 9,3%, lo que representa el 16,9% de las respuestas). Además, el 0,85% (4 respuestas) no especificó cómo había recibido la encuesta, mientras que el 0,43% (2 respuestas) declaró haberla recibido por otros medios, específicamente, de un colega y de una organización no gubernamental (ONG) local.

**Características demográficas de los encuestados**

En total, recibimos 468 respuestas de gestores de la conservación que trabajan en 21 países latinoamericanos (Figura 1a). El 55% de los encuestados se identificó como hombre, el 43% como mujer, el 1% prefirió no declarar su identidad de género y el 1% no contestó (0% Otros; Figura 4a). El 35% de los encuestados declaró tener entre 40 y 49 años, el 28% entre 30 y 39 años, el 21% entre 50 y 59 años, el 9% 60 o más años, el 5% menos de 30 años, y un 1% no contestó a esta pregunta. La mayoría de los encuestados (44%) trabajaban en una institución académica o de investigación, el 29% en una ONG, el 26% en un organismo gubernamental, y un 1% en una fundación o entidad de conservación de tierras (Figura 1b). En cuanto a la función desempeñada dentro de sus respectivas organizaciones, la mayoría de los encuestados declaró ser investigador/a en biología (laboratorio o trabajo de campo; 53%), gestor/a de recursos naturales (16%), analista (15%; responsable de políticas públicas, legislación y/o planificación estratégica), otro (10%), y educador/a (6%) (Figura 1c). En cuanto al cargo dentro de su organización, la mayoría de los encuestados declaró tener un rango laboral ejecutivo de nivel medio (investigador; 59%), seguido de jefe (director; 33%) y empleado operativo (estudiante; 7%) (el 0,4% no contestó).

La gran mayoría de los encuestados (97%) eran nacionales de América Latina, seguidos de nacionales de Europa (2%) y América del Norte (1%). En cuanto al país de residencia y la nacionalidad de los encuestados, la mayoría eran residentes (92%) o nacionales (92%) con respecto al país donde se encuentra el área/especie gestionada, mientras que el 87% eran tanto nacionales como residentes. Un porcentaje menor (2%, 10 encuestados) no eran ni nacionales ni residentes del país donde se encuentra el área/especie gestionada. De este último grupo, el 1,4% (7 encuestados) eran latinoamericanos que trabajaban en un país de América Latina distinto de su país de origen, el 0,4% eran norteamericanos que trabajaban en América Latina (2 encuestados) y 1 encuestado era un latinoamericano que gestionaba un área/especie en América Latina pero residía en América del Norte (0,2%).

**Áreas/especies gestionadas y desafíos identificados**

Los encuestados gestionaban áreas o especies situadas en ámbitos terrestres (70%), marinos (15%), y de agua dulce (9%), mientras que el 6% gestionaba áreas o especies en múltiples ámbitos (Figura 1d). La mayoría de los encuestados (69%) trabajaba en la gestión *in situ* (dentro del hábitat natural de la especie), el 24% trabajaba tanto en la gestión *in situ* como *ex situ* (fuera del hábitat natural de la especie), mientras que sólo el 7% de los encuestados trabajaba exclusivamente *ex situ*. Los principales grupos taxonómicos estudiados mediante evaluación genética fueron los animales (71%), seguidos de las plantas (13%), los microorganismos (Bacterias, Archaea, Protistas; 1%), y los hongos (1%), en tanto el 14% de los encuestados declaró haber estudiado múltiples taxones.

Los encuestados declararon que los desafíos más importantes en relación con su área/especie gestionada eran: (i) Evaluar el tamaño de la población (muy importante: 67%; importante: 21%), (ii) Mantener la conectividad o identificar corredores (muy importante: 63%; importante: 20%), (iii) Identificar unidades de manejo (muy importante: 56%; importante: 25%), (iv) Delimitar poblaciones (muy importante: 50%; importante: 25%), (v) Elaborar inventarios de especies (muy importante: 45%; importante: 21%), (vi) Evaluar características de historia de vida (muy importante: 40%; importante: 26%), (vii) Evaluar la presencia de endogamia o parentesco de los individuos (muy importante: 32%; importante: 18%), y (viii) La detección/prevención de la hibridación (muy importante: 23%; importante: 13%).

**Estudios genéticos en el área/especie gestionada**

La mayoría de los encuestados (79%) indicó que ya habían considerado la posibilidad de utilizar la genética para apoyar sus prácticas de gestión, y el 67% declaró que sabría cómo realizar un estudio genético en su área/especie gestionada si estuviera interesado en hacerlo. Además, el 25% declaró haber realizado un inventario de biodiversidad o haber identificado especies con técnicas de códigos de barras de ADN o de ADN ambiental.

En cuanto a la utilidad de la información genética, los encuestados la consideraron extremadamente útil o útil para: (i) Informar las acciones de manejo (82%), (ii) Establecer información de base sobre el área gestionada (por ejemplo, censo de población o composición de especies) (82%), (iii) Evaluar la eficacia de las acciones de manejo (75%), y (iv) Informar la protección o acciones legislativas (71%).

La mayoría de los encuestados (65%) declaró haber realizado o utilizado un estudio genético en su área/especie gestionada (Figura 2a). Entre estos, el 51% realizó el estudio solo/a o en colaboración, el 27% solicitó/contrató a otra persona para que realizara la evaluación, y el 22% utilizó datos genéticos publicados (Figura 2b). El porcentaje de encuestados que había utilizado/realizado un estudio genético en su área/especie gestionada fue mayor entre los académicos (70%) que entre los no académicos (60%). Consecuentemente, el porcentaje de encuestados que no había utilizado/realizado un estudio genético fue mayor entre los no académicos (39%) que entre los académicos (29%; Figura 2c).

Para la proporción total de encuestados de procedencia académica o no académica que no habían realizado o utilizado un estudio genético en su área/especie gestionada (35%; Figura 2a), las principales barreras o factores limitantes a tal efecto fueron, por orden de importancia: (i) Acceso limitado a financiamiento (48%), (ii) Acceso limitado a un laboratorio de genética (37%), (iii) Falta de ayuda u orientación en el diseño de una evaluación genética (33%), (iv) Falta de personal calificado para realizar el trabajo de laboratorio (33%), (v) Acceso limitado a muestras (24%), (vi) Falta de conocimiento sobre las preguntas que pueden abordarse (22%), (vii) Falta de confianza en la aplicabilidad de los resultados genéticos a las decisiones de gestión (19%) y (viii) Falta de personal calificado para realizar el trabajo de campo (18%) (Figura 3a).

En cuanto a la aplicabilidad de los resultados de la evaluación genética, los datos recabados muestran que: (i) el 41% de los académicos y el 46% de los no académicos están ‘completamente en desacuerdo’ o ‘en desacuerdo’, respectivamente, con la afirmación "Los resultados fueron demasiado técnicos", (ii) el 82% de los académicos y el 79% de los no académicos están ‘completamente en desacuerdo’ o ‘en desacuerdo’ con la afirmación "Los resultados no fueron aplicables a nuestros objetivos de manejo para conservación", y (iii) el 71% de los académicos y el 71% de los no académicos están ‘completamente de acuerdo’ o ‘de acuerdo’ con la afirmación "Los resultados ayudaron a informar las decisiones de manejo" (Figura 3b).

**Codesarrollo de proyectos de conservación y disponibilidad/compartición de datos**

Para describir mejor la colaboración entre académicos y profesionales en América Latina, preguntamos quién había planteado inicialmente las preguntas abordadas por la evaluación genética. Nuestros resultados muestran que para la mayoría de los académicos encuestados (58%), las preguntas de investigación fueron planteadas por ellos mismos o por alguien de su grupo, en comparación con el 25% de los no académicos. En cambio, la mayoría de los encuestados no académicos (46%) plantearon las preguntas en colaboración con socios ajenos a su organización, frente al 35% de los académicos. Sólo el 7% de los académicos declararon que las preguntas surgieron de un grupo ajeno a su organización, frente a una proporción mucho mayor de no académicos (29%).

Nuestros resultados también muestran que el 64% de los académicos y el 65% de los no académicos declararon que los estudios genéticos habían finalizado y los resultados se habían puesto a disposición de los gestores en el momento en que finalizó nuestra encuesta (16 de noviembre de 2021), mientras que el 11% de los académicos y el 7% de los no académicos declararon que los estudios habían finalizado, pero que hasta tal fecha los resultados no se habían puesto a disposición de los gestores. En tanto, el 25% de los académicos y el 28% de los no académicos encuestados declararon que las evaluaciones estaban en curso y, por tanto, los resultados no estaban disponibles al finalizar nuestra encuesta.

**Alianzas entre grupos u organizaciones**

Para comprender mejor las barreras o desafíos para iniciar y/o mantener alianzas o asociaciones entre investigadores en genética de la conservación y gestores en América Latina, preguntamos a los gestores qué probabilidad tenían de ponerse en contacto con diferentes grupos como socios potenciales para realizar un estudio genético. Nuestros resultados muestran que los encuestados se pondrían en contacto, por orden de importancia, con: (i) un laboratorio académico (‘extremadamente probable’ o ‘probable’, 88%); (ii) otra persona/unidad/sucursal en su organización (‘extremadamente probable’ o ‘probable’, 68%); (iii) una ONG (‘extremadamente probable’ o ‘probable’, 53%); (iv) una organización gubernamental (‘extremadamente probable’ o ‘probable’, 41%; ‘baja probabilidad’ o ‘improbable’, 39%); (v) una empresa consultora privada (‘baja probabilidad’ o ‘improbable’, 55%), mientras que (vi) el 71% no se pondría en contacto (‘baja probabilidad’ o ‘improbable’) con ningún grupo y la llevaría a cabo por sí mismo.

Además, nuestros resultados muestran que, si a los gestores se les ofreciese ayuda de parte de un genetista académico para diseñar y/o llevar a cabo un estudio genético, el 80% de aquellos estaría dispuesto a aceptar, el 19% tal vez aceptaría, y el 1% no aceptaría. En tanto, si un servicio de consultoría no académico estuviera disponible para ayudar a los gestores a diseñar y llevar a cabo una evaluación genética, el 51% de los encuestados declaró que ellos o sus organizaciones tal vez buscarían su ayuda, el 32% la buscaría, y el 17% no buscaría ayuda.

Más de la mitad de los encuestados (56%) declararon que habían sido contactados para realizar un estudio genético en su área/especie gestionada y que la solicitud procedía de los siguientes grupos, por orden de prevalencia: (i) laboratorio académico externo (44%), (ii) otra persona/unidad de su organización (22%), (iii) ONG externa (16%), (iv) organización gubernamental externa (13%) y (v) empresa consultora privada (5%). Una mayoría considerable de estos contactos procedía de la propia América Latina (70%), seguida de América del Norte (17%), Europa (8%), Otros (2%) y sin respuesta (3%).

**Brecha de género**

Nos interesaba evaluar si existía una brecha de género entre mujeres y hombres en el ámbito del manejo para la conservación en América Latina. Nuestros resultados muestran que el 63% de las mujeres gestoras ocupaban puestos ejecutivos de nivel medio (investigadoras) y el 28% ocupaban puestos de dirección o de mayor rango (directoras). Por el contrario, una mayor proporción de gestores hombres ocupaba puestos de director o de rango superior (37%), y el 56% ocupaba puestos ejecutivos de nivel medio (Figura 4b).

En cuanto a los encuestados que declararon haber sido contactados por algún grupo de investigación latinoamericano para realizar una evaluación genética, preguntamos por el género del líder del grupo que se había puesto en contacto con ellos. La representación de las mujeres en tales funciones de liderazgo (40%) fue inferior en comparación con la de los hombres (51%), y un 9% indicó no saber, prefirió no declarar u otro (Figura 4b).

**Discusión**

Este estudio ofrece un diagnóstico general de la situación actual de la brecha entre conservación y genética en América Latina. A partir de los resultados obtenidos, ofrecemos ideas y soluciones para superar obstáculos y avanzar agendas que se ajusten a las necesidades y realidades de este territorio altamente heterogéneo, biodiverso y desafiante (Figura 5).

El éxito global del muestreo fue mucho mayor a través de los puntos focales (28,8%) que recurriendo a las listas de correo de las organizaciones (9,3%). La distribución a través de los puntos focales también facilitó el alcance de la encuesta entre los encuestados (proveyendo el 81,8% de las respuestas totales). La naturaleza personalizada de las invitaciones enviadas por los puntos focales a los encuestados objetivo probablemente fomentó un mayor sentimiento de confianza y relevancia entre los participantes potenciales, aumentando así la motivación para responder. Por el contrario, el enfoque más impersonal de utilizar toda la lista de correo de las organizaciones puede haber dado lugar a niveles más bajos de compromiso personal, y en consecuencia, a tasas de respuesta reducidas. Abogamos por la adopción generalizada de puntos focales para la realización de encuestas en línea, ya que estos tienen, más probablemente, un mejor conocimiento de los aspectos culturales y las redes locales de sus países, redundando en un mayor éxito a la hora de obtener respuestas.

Los participantes en nuestra encuesta pertenecían a distintos tipos de organizaciones, incluyendo instituciones académicas o de investigación (44%), ONGs (29%) e instituciones gubernamentales (26%) (Figura 1b). La composición demográfica de nuestro estudio difiere de la comunicada por Taft et al. (2020), en la que un mayor porcentaje de participantes procedía de instituciones gubernamentales (50%), seguidas de instituciones académicas (24%) y ONGs (16%). Taft et al. (2020) describieron una tendencia entre los gestores hacia el uso de la genética, así como una mayor incidencia de encuestados que habían realizado o empleado evaluaciones genéticas. En comparación, nuestro estudio describió una menor proporción de estudios genéticos implementados por gestores. Por lo tanto, las diferencias observadas entre nuestro estudio y el de Taft et al. (2020) podrían deberse a un sesgo geográfico o a diferencias en los tipos de instituciones representadas en cada estudio. En general, las naciones desarrolladas destinan más fondos públicos a la investigación científica, incluida la ciencia de la conservación, que las naciones en desarrollo (González-Brambila et al., 2016). Teniendo en cuenta que los encuestados en el estudio de Taft et al. (2020) eran principalmente gestores de instituciones gubernamentales con sede en EE.UU. (50% de su muestra), es probable que aquellos enfrenten menos limitaciones financieras para llevar a cabo evaluaciones de genética de la conservación en comparación con los gestores latinoamericanos. Como resultado, es concebible que en el estudio arriba mencionado los entrevistados posean una mayor conciencia, formación y/o experiencia en este campo, en comparación con sus homólogos latinoamericanos.

En consonancia con Torres-Florez et al. (2018), la mayoría de los encuestados en nuestro estudio trabajaban en el ámbito terrestre (Figura 1d). Los encuestados declararon haber realizado evaluaciones genéticas principalmente en animales, mientras que las plantas estuvieron infrarrepresentadas en nuestra muestra. Sin embargo, otro metaestudio informó que, de todas las publicaciones revisadas sobre genética de la conservación (1980-2010), el 46% trataba sobre plantas (Oliveira-Miranda et al., 2013).

Las respuestas a nuestra encuesta muestran que la mayoría de los gestores de la conservación en América Latina ya habían realizado un estudio genético o utilizado datos genéticos en su área/especie gestionada, y que la mitad de ellos trabajaban solos o en colaboración (Figura 2a, b). De manera alentadora, encontramos que la mayoría de los gestores para la conservación no sólo son conscientes de la utilidad de las evaluaciones genéticas para informar y evaluar las acciones de manejo (Figura 3b), sino que también ya han utilizado la genética y consideran que tienen los conocimientos técnicos necesarios para realizar o diseñar un estudio genético por sí mismos, saben cómo aplicar o interpretar los datos genéticos publicados para lograr sus objetivos de conservación, y/o son capaces de encontrar activamente un colaborador. Encontramos asimismo que una mayor proporción de encuestados académicos, en comparación con los no académicos, había realizado o utilizado la genética para apoyar sus objetivos de conservación. Esta situación probablemente refleja el mayor acceso a la investigación actualizada, las oportunidades de formación y las redes de colaboración dentro de los entornos académicos, en contraste con los no académicos. En particular, nuestros hallazgos contrastan con otros estudios que han indicado una falta de concientización y conocimiento sobre la utilidad de incluir datos genéticos en las acciones de conservación (Cook et al., 2013; Klütsch & Laikre, 2021). Este prometedor escenario pone de relieve los beneficios potenciales de promover un sistema de red de mentores dentro de la comunidad de manejo para la conservación (ver detalles al respecto en "Acortando distancias: recomendaciones para avanzar").

Un desafío importante en América Latina debe estar enfocado en aquellos gestores/as que no han realizado o utilizado evaluaciones genéticas, quienes representan un tercio de nuestra muestra (Figura 2a). Estos encuestados indicaron que las principales barreras para la implementación de la genética son: el acceso limitado a financiamiento, el acceso limitado a un laboratorio de genética, la falta de ayuda u orientación en el diseño de un estudio genético, y la falta de personal calificado para llevar a cabo el trabajo de laboratorio (Figura 3a). Esto hace eco de otros estudios que muestran que la mayoría de las limitaciones para la implementación efectiva de la genética en las acciones de conservación en América Latina son operativas o financieras, en lugar de estar relacionadas con la falta de confianza en la genética (ver por ejemplo Ramírez et al., 2020).

Además, y en consonancia con encuestas realizadas en diversas regiones del mundo (Haig et al., 2016; Taylor et al., 2017; Holderegger et al., 2019; Klütsch & Laikre, 2021), algunos de los encuestados en nuestro estudio (el 25% de los académicos y el 30% de los no académicos) consideran que los resultados genéticos son demasiado técnicos para interpretarlos (Figura 3b). Esto no es sorprendente, dado que la genética de la conservación, como campo de investigación, requiere el uso de terminología técnica y conceptos teóricos complejos, que implican necesariamente cierta formación y conocimientos básicos para ser comprendidos. Además, los conceptos de genética de la conservación no suelen estar presentes en la educación secundaria ni en los cursos técnicos postsecundarios en América Latina (UNESCO, 2021).

Se observa que un menor número de encuestados no académicos (25%) definen las preguntas de estudio que abordan las evaluaciones genéticas, frente a una mayor proporción de académicos (58%) que lo hacen, ya sea individualmente o en colaboración. Recomendamos que se realicen esfuerzos para estrechar los lazos entre genetistas y gestores, de modo que codiseñen las evaluaciones genéticas desde el principio, definiendo conjuntamente las preguntas de estudio y eligiendo la metodología a aplicar, todo ello en conexión con las necesidades de los gestores.

Una elevada proporción de los encuestados en nuestro estudio, tanto académicos (64%) como no académicos (65%), afirma que cuando trabajaban en colaboración los resultados genéticos estaban fácilmente disponibles para ellos. Esto contrasta con otros estudios que lamentan que los resultados obtenidos de trabajos en genética de la conservación permanezcan en gran medida en el ámbito académico (Cook et al., 2013; Sandström et al., 2016; Taylor et al., 2017; Klütsch & Laikre, 2021). Nuestros datos indican por tanto un escenario positivo, dado que el intercambio de resultados científicos es la base para transformar la información en prácticas de manejo eficaces y para crear colaboraciones a largo plazo que inspiren confianza y tengan éxito.

Los resultados de nuestra encuesta también indican que es más probable que los gestores de América Latina se pongan en contacto con un laboratorio académico que con una empresa consultora privada para realizar una evaluación genética. Además, la mayoría de los gestores en conservación se inclinarían por aceptar la ayuda de un genetista académico para diseñar o llevar a cabo una evaluación genética, prefiriéndolo a un servicio de consultoría privado. Dado que los servicios ofrecidos por el sector privado tienden a ser más costosos, estas respuestas podrían poner de manifiesto la falta de financiamiento de los gestores en América Latina para pagar los servicios de empresas privadas (por lo que recurren a colaboraciones académicas que suelen depender del financiamiento obtenido por los investigadores) y/o reflejar la alta reputación de las instituciones académicas, consideradas de excelencia, con autonomía y socialmente responsables (Alzyoud et al., 2015). Esto enfatiza aún más la necesidad de promover un mayor financiamiento para infraestructura y capacitación de recursos humanos y personal en instituciones académicas y públicas que realizan investigación teórica y aplicada en genética de la conservación en América Latina.

Una menor proporción de los encuestados (43%) se autodeclaró mujer, en comparación con un 55% que declaró ser hombre (Figura 4a). Diferentes hipótesis, que no son mutuamente excluyentes pero que están fuera del alcance de este estudio, pueden explicar esta diferencia. La misma podría reflejar una menor participación de las mujeres en el manejo para la conservación en América Latina, o bien que las mujeres efectivamente participan de este, pero no respondieron a la encuesta (presumiblemente porque dedican proporcionalmente más tiempo a las tareas domésticas y a las funciones de cuidado en comparación con los hombres; Amarante et al., 2023; Mukhopadhyay, 2023). Dicha diferencia puede reflejar incluso un sesgo involuntario en la elección de los encuestados contactados por nuestros 32 puntos focales (a pesar de que casi dos tercios (63%) de ellos eran mujeres) o una subrepresentación en las listas de correo de las organizaciones colaboradoras (SCB, ReGeneC, LACA-SCB, Sociedad Mesoamericana de Biología y Conservación). Entre las encuestadas que se autodeclararon mujeres, una menor proporción (28%) ocupa cargos de jefatura o de mayor rango (directora) dentro de sus organizaciones, en comparación con los hombres (37%) (Figura 4b). De manera similar, una menor proporción de mujeres (40%), en comparación con los hombres (51%), lidera grupos de investigación que realizan evaluaciones genéticas (Figura 4c). Las brechas y diferencias de género en las áreas de ciencia, tecnología, ingeniería y matemáticas (STEM, por sus siglas en inglés) han sido ampliamente descritas y estudiadas en todo el mundo (Foro Económico Mundial, 2023), y también en América Latina (Braverman-Bronstein et al., 2023; García-Holgado et al., 2020; Osorio-Delvalle et al., 2020; Lappe et al., 2021). Durante las últimas décadas se han logrado avances significativos en América Latina para mejorar la igualdad de género en STEM (García-Holgado et al., 2019; Mosquera & Rodríguez, 2023), y aunque algunos países están avanzando bien hacia la reducción de la brecha de género (Foro Económico Mundial, 2023), todavía hay mucho margen de mejora.

También exploramos la posible ocurrencia y extensión de la ciencia de la conservación colonial o paracaidista en América Latina mediante la evaluación de la ciudadanía y residencia de los encuestados. Nuestros resultados muestran un escenario prometedor, indicando el potencial para el empoderamiento local en el manejo para la conservación, dado que la gran mayoría de los encuestados eran residentes (92%) o nacionales (92%), o ambos (87%), de los países donde se encuentra su área/especie gestionada. Más aún, nuestros resultados muestran que en la región existen formación y capacidades locales para llevar a cabo tanto el manejo para la conservación de la biodiversidad local como evaluaciones genéticas (Figuras 2a,b, 3b y ver Resultados). Nuestros hallazgos están en línea con los reportados en una revisión sobre genética de la conservación de plantas (Oliveira-Miranda et al., 2013) que describió un aumento en la participación de latinoamericanos como primeros autores (de 46% en la década de 1990 a 71% en 2006-2010), y un aumento en el número de publicaciones de autoría exclusiva de latinoamericanos. Oliveira-Miranda et al. (2013) también encontraron que el número de estudios en genética de la conservación de plantas en América Latina con sólo coautores extranjeros era mayor en los países situados entre México y Panamá, mientras que los que incluían solo coautores locales era mayor en aquellos ubicados desde Colombia y hasta Brasil y Bolivia.

Este estudio fue diseñado para priorizar una adecuada distribución geográfica del muestreo a lo largo de los países latinoamericanos, siendo uno de los primeros en realizar una amplia encuesta en toda la región para describir la brecha entre la conservación y la genética (Ceballos et al., 2009; Taylor et al., 2017; Fabian et al., 2019; Taft et al., 2020). Se destaca además que la representación geográfica de nuestros resultados es más amplia y extensa que la de estudios anteriores (Rojas Bonzi et al., 2018; Taft et al., 2020). No obstante, debemos señalar que los resultados de nuestra encuesta no pueden tomarse como una descripción de la naturaleza compleja de toda la población de gestores para la conservación de América Latina, porque la distribución de la misma no fue aleatoria. Sólo podemos describir y discutir nuestros resultados basándonos en nuestra muestra, considerando la información de los encuestados y reconociendo posibles sesgos. A pesar de nuestros esfuerzos por garantizar una muestra diversa y equilibrada, dirigida a encuestados con formación académica y no académica, y con diversas afiliaciones organizativas, reconocemos las limitaciones potenciales del presente estudio. En este sentido, los puntos focales podrían haber contactado involuntariamente a más personas que comparten un entusiasmo similar por el uso de datos genéticos en los esfuerzos de conservación, a pesar de que desde el principio se estableció un claro esfuerzo para evitar este sesgo. Por lo tanto, al igual que en Taft et al. (2020), nuestra muestra podría incluir una mayor representación de gestores que tenían conocimiento de, o estaban interesados o capacitados en, la investigación genética, en comparación con su proporción real en toda América Latina. Además, el uso de listas de correo de ciertas organizaciones, por ejemplo, la de la Red Latinoamericana de Genética de la Conservación (ReGeneC), también puede haber inducido un sesgo de muestreo, aunque su representación específica en nuestra muestra fue mucho menor (6,8%) en comparación con los puntos focales (81,8%). Nuestra muestra podría haber estado desequilibrada también en otros aspectos, como el hábitat o ámbito en el que trabajan los encuestados (principalmente terrestre, 70%; Figura 1d), o los grupos taxonómicos estudiados (principalmente animales, 71%). No obstante, creemos que nuestra muestra refleja la diversidad de gestores para la conservación presentes en América Latina en cuanto a tipos de organizaciones, función de los encuestados, y modalidad de gestión (*in situ* y/o *ex situ*) (Figura 1c; ver Resultados). Cabe mencionar asimismo que el sesgo de deseabilidad social (es decir, la tendencia a alinear las respuestas con lo que se percibe como socialmente aceptable o políticamente correcto) puede limitar la interpretación de nuestros hallazgos, como ocurre en muchos otros estudios de investigación cualitativa que utilizan encuestas (Bergen & Labonté, 2020).

Al interpretar y discutir nuestros resultados, reconocemos la alta heterogeneidad de las realidades locales y las condiciones de trabajo que coexisten a través de los países latinoamericanos con respecto a la investigación y el desarrollo científico, las capacidades de infraestructura, la formación especializada y el acceso a la información científica publicada (UNESCO, 2021). Aunque los países latinoamericanos comparten historias y escenarios socioeconómicos similares (Ceballos et al., 2009, Torres-Florez et al., 2018), existen diferencias significativas en toda la región. Por ejemplo, Brasil, Argentina, México y Chile están más avanzados en la integración de la genética en la gestión de la conservación en comparación con otros países latinoamericanos (Oliveira-Miranda et al., 2013; Torres-Florez et al., 2018) debido a una miríada de factores como diferencias en las oportunidades de financiamiento y/o legislación, barreras de implementación y formas posibles en que la genética puede aplicarse a la conservación (ver por ejemplo Torres-Florez et al., 2018; Taft et al., 2020). En el contexto de esta región tan heterogénea, la gestión de la conservación de especies con áreas de distribución transnacionales constituye un enorme desafío. La marcada diversidad que observamos a este respecto en toda América Latina es probablemente mucho menos pronunciada en el Norte Global (UNESCO, 2021). Por lo tanto, a pesar del gran tamaño de nuestra muestra (n=468 encuestados), los resultados y conclusiones generales de nuestro estudio pueden enmascarar las diferencias entre los países latinoamericanos, ya que no fue diseñado para abordar cada uno en detalle. Futuros estudios deberían abordar las diferencias en toda la región latinoamericana utilizando una muestra de mayor tamaño por país para permitir una evaluación más profunda.

**Acortando distancias: recomendaciones para avanzar**

Las estrategias para que los profesionales incorporen la genética en sus prácticas de manejo para la conservación, y para subsanar la brecha entre conservación y genética existente entre gestores e investigadores, deben ser específicamente diseñadas para América Latina. Basándonos en nuestros resultados, ofrecemos algunas recomendaciones, mecanismos y ejemplos orientadores para alcanzar estos objetivos:

**(1) CAPACITACIÓN: Promover y ampliar las oportunidades de capacitación técnica y teórica para gestores, profesionales y tomadores de decisiones en temas de genética de la conservación.**

● Desarrollar y difundir cursos cortos y diplomados en genética de la conservación específicamente para gestores, profesionales de la conservación y responsables de la toma de decisiones.

● Formar a gestores y responsables de la toma de decisiones en el uso de la terminología asociada a la genética de la conservación, para ayudarles a interpretar los resultados de informes técnicos y artículos científicos.

● Reforzar la enseñanza de la genética de la conservación en las escuelas secundarias, los cursos técnicos de secundaria, las licenciaturas en ciencias biológicas y los cursos de posgrado relacionados con la conservación de la biodiversidad.

- Organizaciones, asociaciones o sociedades ofrecen cursos cortos para reforzar los conocimientos generales (por ejemplo, la Asociación Paraguaya de Mastozoología ofrece cursos introductorios de Genética de la Conservación durante los congresos).

- Cursos de Genética de la Conservación ofrecidos por programas universitarios de posgrado (por ejemplo, Especialización en Biología de la Conservación, Universidad Nacional de Misiones, Argentina).

- Desde 2005, ReGeneC ofrece anualmente un curso de Genética de la Conservación para estudiantes de posgrado latinoamericanos (<https://regenec.org>).

● Proporcionar oportunidades y financiamiento para becas, programas de intercambio y estancias cortas para visitar laboratorios de genética de la conservación dentro de América Latina.

- Fondos disponibles para estancias cortas: En Paraguay, CONACYT (<https://www.conacyt.gov.py/conacyt-lanza-oportunidad-para-realizar-estancias-investigacion-corta-duracion>); en Brasil, Programa Move La América (<https://www.gov.br/capes/pt-br/acesso-a-informacao/acoes-e-programas/bolsas/bolsas-e-auxilios-internacionais/encontre-aqui/paises/multinacional/programa-move-la-america>).

**(2) COLABORACIÓN: Fomentar la interacción y colaboración constantes entre investigadores académicos, gestores y responsables de la toma de decisiones para llevar a cabo proyectos de conservación e integrar la genética en estrategias de conservación más amplias.**

● Como parte de su responsabilidad social y de su rol público, las instituciones académicas y de investigación deben reforzar la comunicación, el compromiso y la colaboración regulares con las organizaciones no académicas de gestión y manejo para la conservación.

● Fomentar una estrecha comunicación entre científicos y gestores con experiencia local sobre el terreno, desde el inicio de los proyectos de conservación y a lo largo de todas las etapas de los mismos, facilitando la comunicación de las necesidades, ayudando a enmarcar y codiseñar las preguntas de investigación, promoviendo la retroalimentación directa sobre la viabilidad de la aplicación de las recomendaciones y acciones de conservación, y fortaleciendo colaboraciones de confianza a largo plazo.

● Promover enfoques multi, inter y transdisciplinares, cooperación y retroalimentación activas entre disciplinas, y diálogo constante entre los actores clave y las partes interesadas.

- Realizar reuniones periódicas entre investigadores/genetistas y gestores/responsables de la toma de decisiones a nivel local para presentar y debatir los resultados de investigación.

- Organizar periódicamente mesas redondas y paneles intersectoriales para el debate técnico entre investigadores en genética y responsables de la toma de decisiones que necesitan datos genéticos para mejorar la toma de decisiones para la conservación y gestión de la biodiversidad basadas en evidencia.

- Abrir instancias de diálogo periódico entre investigadores y organismos gubernamentales encargados de los recursos ambientales para debatir cómo la información genética puede ayudar a delinear y lograr estrategias más amplias de conservación y desarrollo sostenible.

● Facilitar el acceso y la interpretación de los resultados de la investigación a gestores, profesionales y responsables de la toma de decisiones.

- Instar a genetistas e investigadores a redactar informes de forma sencilla, directa y aplicada, para compartirlos con gestores, profesionales y responsables de la toma de decisiones.

- Formar a los investigadores en técnicas de comunicación científica para ayudarles a difundir los resultados de forma más sencilla.

- Promover que los investigadores publiquen los artículos de investigación en español, portugués, o en la lengua local, para hacerlos más accesibles a los gestores, profesionales y responsables de la toma de decisiones para su aplicabilidad inmediata en las prácticas locales de conservación (por ejemplo, si se publican en una revista en inglés, incluir una versión en la lengua local en el Material Suplementario o en los Apéndices).

● Implementar una plataforma digital abierta para reunir a genetistas y gestores de toda América Latina interesados en colaborar: registrarse, encontrar colegas en la región, comunicarse e interactuar, unirse a un sistema de red de mentores donde los profesionales que ya han utilizado la genética puedan guiar, apoyar y compartir conocimientos con sus colegas que no lo han hecho, y acceder a material de aprendizaje sobre herramientas de genética para la conservación (Figura 5).

● Aumentar los incentivos para que los académicos consideren la posibilidad de colaborar con los gestores y responsables de la toma de decisiones para aplicar conjuntamente mejores estrategias y prácticas de manejo para la conservación.

- Las agencias nacionales y sus programas de financiamiento (por ejemplo, CONACYT [Paraguay], CONICET [Argentina], ANID [Chile], CNPq [Brasil]), así como las universidades y las instituciones de investigación deberían reconocer y considerar las colaboraciones entre académicos y gestores a la hora de puntuar las propuestas de financiamiento y a postulantes durante la contratación y la progresión en la carrera profesional.

- Los empleadores deberían animar y motivar a los investigadores para que participen en actividades que promuevan la interacción con los gestores de la conservación, como reuniones en las que los responsables de la toma de decisiones y los científicos puedan debatir directamente sus necesidades y encontrar puntos en común para la colaboración.

- Los empleadores deberían valorar y animar a los genetistas e investigadores a redactar informes de forma sencilla, directa y aplicada, y en el idioma local.

**(3) FINANCIAMIENTO:** **Promover un mayor financiamiento para infraestructura e implementación de proyectos en instituciones académicas y públicas que realizan genética de la conservación en América Latina.**

● Los consorcios internacionales y los programas intergubernamentales patrocinados por países desarrollados deberían brindar oportunidades para superar las menores capacidades financieras de las instituciones públicas y académicas de América Latina (por ejemplo, GenoTropics; [www.genotropics.org](file:///C:\Users\constanzanapolitano\Desktop\www.genotropics.org)).

● Instar al sector privado a aumentar la inversión en genética de la conservación.

● Promover proyectos de investigación y capacitación en genética de poblaciones en áreas protegidas transfronterizas para evaluar el alcance de las estrategias de conservación de especies amenazadas.

**(4) PARTICIPACIÓN DE LAS MUJERES: Fomentar y aumentar la participación de las mujeres en la gestión de la conservación y en la toma de decisiones.**

● Proporcionar oportunidades específicas para que las mujeres asciendan a rangos superiores en sus organizaciones y obtengan puestos de liderazgo.

● Promover la formación y el financiamiento específicos para las mujeres en el campo de la conservación.

- La Organización para las Mujeres en Ciencia para el Mundo en Desarrollo (en inglés *Organization for Women in Science for the Developing World*, OWSD) ofrece financiamiento y otras oportunidades a sus miembros.

- La Red de Mujeres en la Naturaleza (en inglés *Women in Nature Network*, WiNN) tiene un Programa de Mentores y formación en liderazgo para empoderar a las mujeres en la conservación y la gestión y ayudarlas a prosperar en sus carreras.

**(5) POLÍTICAS: Integrar el uso de la genética en las políticas regionales de conservación y promover políticas de justicia, equidad y diversidad en las colaboraciones de investigación.**

● Integrar los temas de genética de la conservación en debates más amplios sobre estrategias y políticas de conservación de la biodiversidad, organizando foros específicos con responsables de la toma de decisiones, desde gestores de áreas protegidas hasta responsables de políticas de conservación, para incorporar la aplicación de la genética a la conservación.

- Actualizar las Estrategias y Planes de Acción Nacionales de Biodiversidad (EPANB) de los países para cumplir los objetivos del marco del Marco Mundial Kumming-Montreal del Convenio sobre la Diversidad Biológica (CDB).

- Definir estrategias nacionales de conservación de la biodiversidad.

● Difundir, promover y hacer cumplir políticas de justicia, equidad y diversidad en las colaboraciones de investigación tanto en contextos científicos como profesionales.

- Crear y hacer cumplir acuerdos y directrices de cooperación claros entre instituciones y países para facilitar el transporte de muestras y el intercambio de resultados genéticos en colaboraciones con fines de conservación.

- Difundir y hacer cumplir regulaciones claras sobre permisos para realizar investigación y acceder a recursos genéticos, especialmente en el contexto de alta biodiversidad y altas amenazas en la región latinoamericana (por ejemplo, el Protocolo de Cartagena sobre Seguridad de la Biotecnología del Convenio sobre la Diversidad Biológica (<https://bch.cbd.int/protocol>), el Protocolo de Nagoya sobre Acceso y Participación en los Beneficios (<https://www.cbd.int/abs>), y diferentes regulaciones nacionales.

● Los investigadores y gestores deben promover e integrar perspectivas de género, diversidad e inclusión, y evitar la discriminación y los prejuicios en la realización de estudios de genética de la conservación.

● Promover el liderazgo colectivo y las alianzas entre instituciones del Norte Global y del Sur Global hacia prácticas de conservación de la biodiversidad respetuosas y justas (Ruelas Inzunza et al., 2023; Soares et al., 2023).

- Contactar con latinoamericanos residentes en el Norte Global y en el Sur Global para ayudar a forjar colaboraciones y asociaciones internacionales.

**Agradecimientos**Los autores desean agradecer a todos los gestores de conservación de América Latina que amablemente respondieron a la encuesta. Un agradecimiento especial al Grupo de Trabajo de Genética de la Conservación de la SCB y especialmente a Soraia Barbosa por sus fructíferas discusiones. También queremos agradecer a Emma Gleeman y Steve Flaherty por la corrección de la versión en inglés de la encuesta y el apoyo de Esperanza Beltrami, Carolina Ugarte y Camila Stuardo. Agradecemos a Michael G. Handford y a los revisores anónimos sus valiosos comentarios y sugerencias sobre el manuscrito. CN desea agradecer a la Agencia Nacional de Investigación y Desarrollo de Chile (ANID, Chile) por el apoyo brindado a través de los siguientes fondos: ANID PAI 77190064, ANID Fondecyt Regular 1220758, ANID PIA/BASAL FB210006, ANID/BASAL FB210018.

**Conflictos de intereses**Los autores no tienen conflictos de intereses que comunicar.

**Contribuciones de los autores**CN y CC concibieron la idea del estudio. CN dirigió el estudio y definió su enfoque. CN, FC, CC, MO-M, AMGM, y MM-V diseñaron e implementaron la encuesta. Todos los autores participaron en la distribución de la encuesta. KPS, AS-M, MO-M, y MM-V prestaron su apoyo distribuyendo la encuesta a través de organizaciones colaboradoras. CN y FC analizaron las respuestas de la encuesta. CN redactó el borrador principal. CN, CC, VR-B, CIM, JFG-M, NB, AG, AMGM, y CYM participaron en la redacción de secciones específicas del manuscrito. Todos los autores participaron en la redacción y revisión del manuscrito.

**Declaración sobre la disponibilidad de los datos**Las respuestas a la encuesta son confidenciales y, por tanto, no se puede acceder a ellas. Se puede solicitar un resumen de los datos al autor correspondiente.

**Declaración ética**

La encuesta fue aprobada por el Comité de Bioética del Instituto de Ecología y Biodiversidad (IEB) de Chile el 12 de agosto de 2021.

**Figuras**

**Figura 1.** Características de los gestores de la conservación en América Latina según 468 encuestados. (a) Mapa político de América Latina con los países coloreados según el número de encuestados. (b) Tipo de organización a la que pertenecen los encuestados. (c) Función o rol de los encuestados en sus respectivas organizaciones. Investigador(a) en biología (laboratorio o trabajo de campo); analista (responsable de la toma de decisiones en políticas públicas, legislación, planificación estratégica). (d) Tipo de ambiente en el que se encuentra el área/especie gestionada por el encuestado.

**
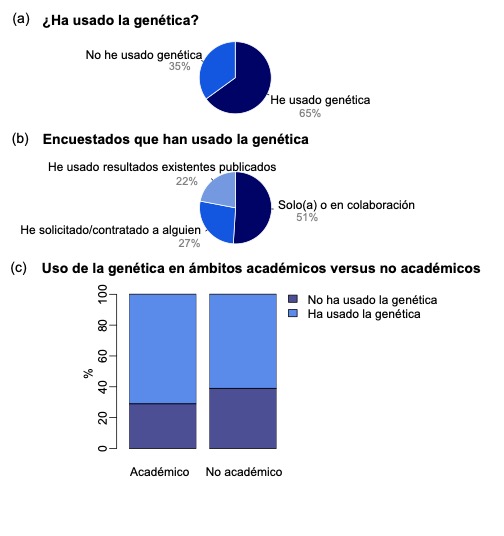
**

**Figura 2.** Utilización de evaluaciones genéticas por los gestores de la conservación en América Latina. (a) Los encuestados responden a la pregunta sobre si han utilizado o no la genética en sus áreas/especies gestionadas. (b) Los encuestados responden a la pregunta sobre cómo utilizaron la genética en sus áreas/especies gestionadas (realizaron el estudio solos o en colaboración; solicitaron o contrataron a otra persona para realizar la evaluación, o utilizaron resultados genéticos ya publicados). (c) Uso de evaluaciones genéticas en encuestados académicos frente a no académicos.

**
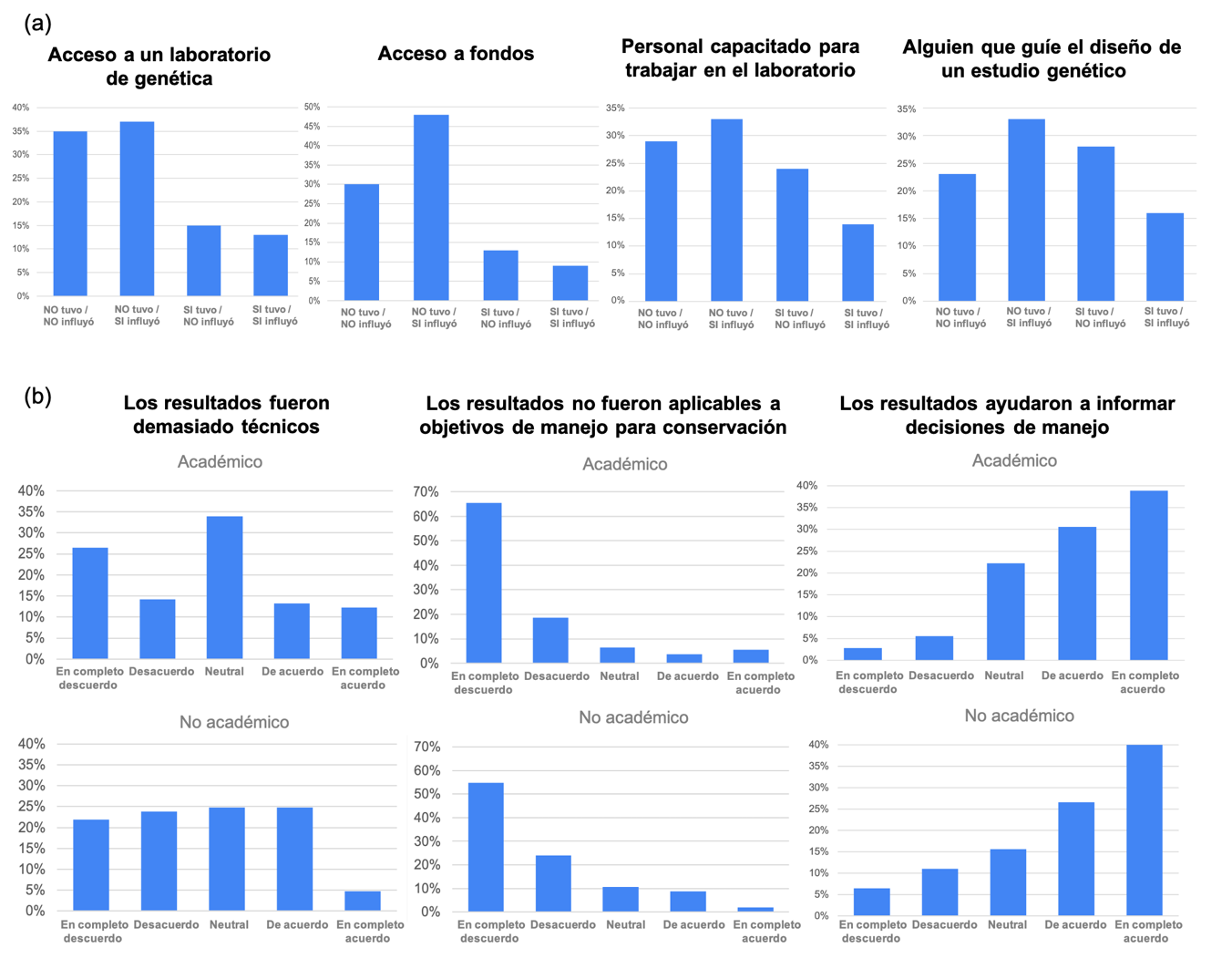
**

**Figura 3.** Principales barreras y utilidad de las evaluaciones genéticas para los gestores de la conservación en América Latina. (a) Principales limitaciones de los gestores de la conservación que no han utilizado la genética. Las categorías enumeradas en el eje x muestran si los encuestados tenían o no una condición o situación particular, y si eso influyó o no en su decisión de realizar/utilizar evaluaciones genéticas. Mostramos las categorías con las proporciones más altas para la situación "NO tuvo/SI influyó". (b) Utilidad de los resultados genéticos para los gestores de la conservación. Las categorías enumeradas en el eje x corresponden a las respuestas de los encuestados en una escala Likert de 1 (totalmente en desacuerdo) a 5 (totalmente de acuerdo).

**
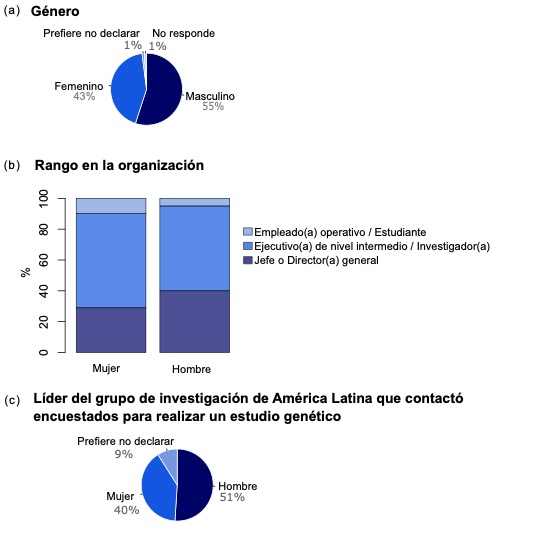
**

**Figura 4.** Brecha de género en los gestores de la conservación latinoamericanos. (a) Género de los encuestados. (b) Participación de mujeres frente a hombres en diferentes rangos de sus organizaciones. (c) Participación de mujeres frente a hombres como líderes de grupos de investigación latinoamericanos que contactaron con los encuestados para realizar una evaluación genética.


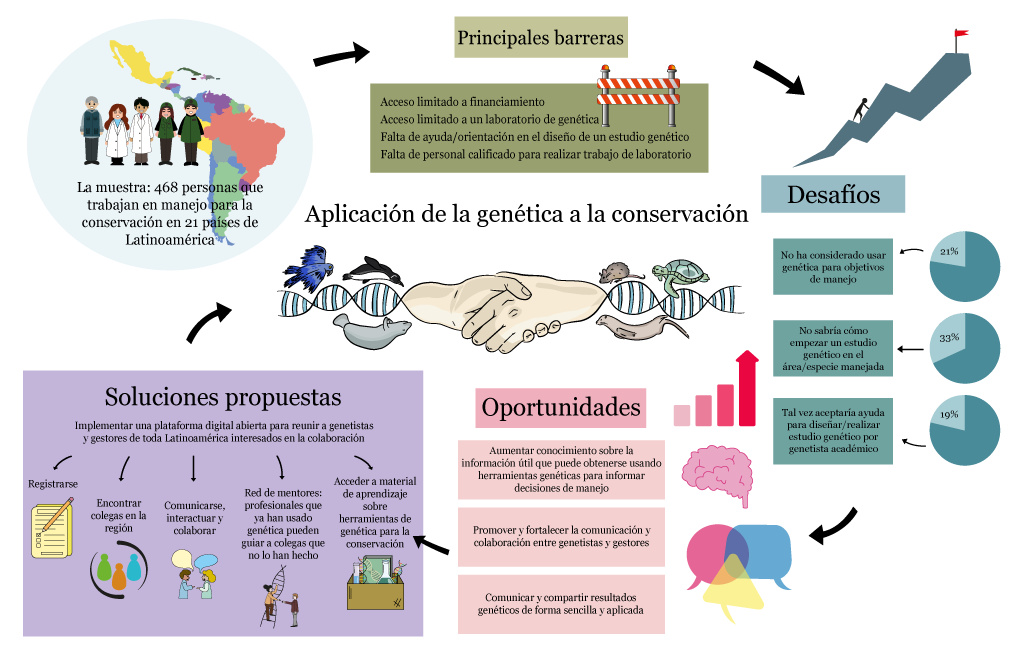


**Figura 5.** Marco conceptual para los profesionales de la conservación en América Latina sobre cómo subsanar la brecha entre la conservación y la genética: Barreras, desafíos, oportunidades y soluciones propuestas (Ilustración de Ahinoam González Marchant).

**Referencias**

Alzyoud, S. A., & Bani-Hani, K. (2015). Social responsibility in higher education institutions: Application case from the Middle East. *European Scientific Journal*, *11*(8): 122-129.

Amarante, V., Rossel, C., & Scalese, F. (2023). Housework and earnings: intrahousehold evidence from Latin America. *Journal of Family Studies*, 1-21.

Asase, A., Mzumara-Gawa, T. I., Owino, J. O., Peterson, A. T., & Saupe, E. (2022). Replacing “parachute science” with “global science” in ecology and conservation biology. *Conservation Science and Practice*, 4(5), e517.

Bergen, N., Labonté, R. (2020). “Everything is perfect, and we have no problems”: detecting and limiting social desirability bias in qualitative research. *Qualitative health research*, *30*(5), 783-792.

Braverman-Bronstein, A., Ortigoza, A. F., Vidaña-Pérez, D., Barrientos-Gutiérrez, T., Baldovino-Chiquillo, L., Bilal, U., ... & Roux, A. V. D. (2023). Gender inequality, women's empowerment, and adolescent birth rates in 363 Latin American cities. *Social Science & Medicine*, *317*, 115566.

Britt, M., Haworth, S. E., Johnson, J. B., Martchenko, D., & Shafer, A. B. (2018). The importance of non-academic coauthors in bridging the conservation genetics gap. *Biological Conservation*, 218, 118-123.

Ceballos, G., Vale, M. M., Bonacic, C., Calvo-Alvarado, J., List, R., Bynum, N., Medellín, R. A., Simonetti, J. A., & Rodríguez, J. P. (2009). Conservation Challenges for the Austral and Neotropical America Section. *Conservation Biology*, *23*(4), 811–817.

Ceballos, G., Ehrlich, P. R. & Raven, P. H. (2020). Vertebrates on the brink as indicators of biological annihilation and the sixth mass extinction. Proceedings of the National Academy of Sciences of the United States of America, 117(24), 13596– 13602.

Cook CN, Mascia MB, Schwartz MW, Possingham HP, Fuller RA. (2013). Achieving conservation science that bridges the knowledge-action boundary. Conservation Biology 27:669–678.

Cook, C. N., & Sgrò, C. M. (2017). Aligning science and policy to achieve evolutionarily enlightened conservation. *Conservation Biology*, *31*(3), 501–512.

Cook, C. N., & Sgrò, C. M. (2018). Understanding managers’ and scientists’ perspectives on opportunities to achieve more evolutionarily enlightened management in conservation. *Evolutionary Applications*, *11*(8), 1371–1388.

Cresswell, J. W., & Plano Clark, V. L. (2011). Designing and conducting mixed method research (2nd ed.). Thousand Oaks, CA: Sage.

de Vos, A., & Schwartz, M. W. (2022). Confronting parachute science in conservation. *Conservation Science and Practice*, *4*(5).

Díaz SM et al. (Eds.). IPBES (2019): Summary for policymakers of the global assessment report on biodiversity and ecosystem services of the Intergovernmental Science-Policy Platform on Biodiversity and Ecosystem Services. IPBES secretariat, Bonn, Germany. 56 pages.

Fabian, Y., Bollmann, K., Brang, P., Heiri, C., Olschewski, R., Rigling, A., ... & Holderegger, R. (2019). How to close the science-practice gap in nature conservation? Information sources used by practitioners. *Biological Conservation*, 235, 93-101.

Ferreira, C. C., & Klütsch, C. F. C. (2021). *Closing the Knowledge-Implementation Gap in Conservation Science* (C. C. Ferreira & C. F. C. Klütsch (eds.); Vol. 4). Springer International Publishing. <https://doi.org/10.1007/978-3-030-81085-6>

Galetti Jr, P. M. (Ed.). (2023). Conservation Genetics in the Neotropics. Springer Nature.

García-Holgado, A., Díaz, A. C., & García-Peñalvo, F. J. (2019). Engaging women into STEM in Latin America: W-STEM project. In *Proceedings of the Seventh International Conference on Technological Ecosystems for Enhancing Multiculturality* (pp. 232-239).

García-Holgado, A., Mena, J., García-Peñalvo, F. J., Pascual, J., Heikkinen, M., Harmoinen, S., ... & Amores, L. (2020). Gender equality in STEM programs: a proposal to analyze the situation of a university about the gender gap. In *2020 IEEE Global Engineering Education Conference (EDUCON)* (pp. 1824-1830). IEEE.

Garner, B. A., Hoban, S., & Luikart, G. (2020). IUCN Red List and the value of integrating genetics. *Conservation Genetics*, *21*(5), 795–801.

Gonzalez-Brambila CN, Reyes-Gonzalez L, Veloso F, Perez-Angón MA. (2016). The Scientific Impact of Developing Nations. *PLoS One* 11(3): e0151328.

Google 2021. Google Forms. Retrieved from <https://www.google.com/forms/>. Web. 26 June.

Haelewaters D, Hofmann TA, Romero-Olivares AL. (2021). Ten simple rules for Global North researchers to stop perpetuating research in the Global South. PLoS Comput Biol 17(8): e1009277.

Haig, S. M., Miller, M. P., Bellinger, R., Draheim, H. M., Mercer, D. M., & Mullins, T. D. (2016). The conservation genetics juggling act: integrating genetics and ecology, science and policy. *Evolutionary Applications*, *9*(1), 181–195.

Hoban, S., Bruford, M., D’Urban Jackson, J., Lopes-Fernandes, M., Heuertz, M., Hohenlohe, P. A., Paz-Vinas, I., Sjögren-Gulve, P., Segelbacher, G., Vernesi, C., Aitken, S., Bertola, L. D., Bloomer, P., Breed, M., Rodríguez-Correa, H., Funk, W. C., Grueber, C. E., Hunter, M. E., Jaffe, R., … Laikre, L. (2020). Genetic diversity targets and indicators in the CBD post-2020 Global Biodiversity Framework must be improved. *Biological Conservation*, *248*, 108654.

Hoban, S., Bruford, M. W., Funk, W. C., Galbusera, P., Griffith, M. P., Grueber, C. E., Heuertz, M., Hunter, M. E., Hvilsom, C., Stroil, B. K., Kershaw, F., Khoury, C. K., Laikre, L., Lopes-Fernandes, M., MacDonald, A. J., Mergeay, J., Meek, M., Mittan, C., Mukassabi, T. A., … Vernesi, C. (2021b). Global commitments to conserving and monitoring genetic diversity are now necessary and feasible. *BioScience*, *71*(9), 964–976.

Hoban, S., Campbell, C. D., da Silva, J. M., Ekblom, R., Funk, W. C., Garner, B. A., Godoy, J. A., Kershaw, F., MacDonald, A. J., Mergeay, J., Minter, M., O’Brien, D., Vinas, I. P., Pearson, S. K., Pérez-Espona, S., Potter, K. M., Russo, I.-R. M., Segelbacher, G., Vernesi, C., & Hunter, M. E. (2021a). Genetic diversity is considered important but interpreted narrowly in country reports to the Convention on Biological Diversity: Current actions and indicators are insufficient. *Biological Conservation*, *261*(July), 109233.

Hoban, S., Hauffe, H. C., Pérez-Espona, S., Arntzen, J. W., Bertorelle, G., Bryja, J., Frith, K., Gaggiotti, O. E., Galbusera, P., Godoy, J. A., Hoelzel, A. R., Nichols, R. A., Primmer, C. R., Russo, I.-R., Segelbacher, G., Siegismund, H. R., Sihvonen, M., Vernesi, C., Vilà, C., & Bruford, M. W. (2013). Bringing genetic diversity to the forefront of conservation policy and management. *Conservation Genetics Resources*, *5*(2), 593–598.

Holderegger, R., Balkenhol, N., Bolliger, J., Engler, J. O., Gugerli, F., Hochkirch, A., ... & Zachos, F. E. (2019). Conservation genetics: Linking science with practice. *Molecular Ecology* 28: 3848-3856.

Horn, L., Alba, S., Blom, F., Faure, M., Flack-Davison, E., Gopalakrishna, G., ... & Kombe, F. (2022). Fostering Research Integrity through the promotion of fairness, equity and diversity in research collaborations and contexts: Towards a Cape Town Statement (pre-conference discussion paper).

Josse, C. E., & Fernandez, M. (2021). Progress and Gaps in Biodiversity Data Mainstreaming and Knowledge Transfer for Conservation in South America. In *Closing the Knowledge-Implementation Gap in Conservation Science* (pp. 255–286).

Kershaw, F., Bruford, M. W., Funk, W. C., Grueber, C. E., Hoban, S., Hunter, M. E., Laikre, L., MacDonald, A. J., Meek, M. H., Mittan, C., O’Brien, D., Ogden, R., Shaw, R. E., Vernesi, C., & Segelbacher, G. (2022). The Coalition for Conservation Genetics: Working across organizations to build capacity and achieve change in policy and practice. *Conservation Science and Practice*, *4*(4), 1–14.

Klütsch, C. F. C., & Laikre, L. (2021). Closing the Conservation Genetics Gap: Integrating Genetic Knowledge in Conservation Management to Ensure Evolutionary Potential. In *Closing the Knowledge-Implementation Gap in Conservation Science* (pp. 51–82).

Liboiron, M. (2021). Decolonizing geoscience requires more than equity and inclusion. *Nat. Geosci.* 14, 876–877.

Laikre, L. (2010). Genetic diversity is overlooked in international conservation policy implementation. *Conservation Genetics*, *11*(2), 349–354.

Laikre, L., Allendorf, F. W., Aroner, L. C., Baker, C. S., Gregovich, D. P., Hansen, M. M., Jackson, J. A., Kendall, K. C., McKelvey, K., Neel, M. C., Olivieri, I., Ryman, N., Schwartz, M. K., Bull, R. S., Stetz, J. B., Tallmon, D. A., Taylor, B. L., Vojta, C. D., Waller, D. M., & Waples, R. S. (2010). Neglect of genetic diversity in implementation of the convention on biological diversity: Conservation in practice and policy. *Conservation Biology*, *24*(1), 86–88.

Laikre, L., Hoban, S., Bruford, M. W., Segelbacher, G., Allendorf, F. W., Gajardo, G., Rodríguez, A. G., Hedrick, P. W., Heuertz, M., Hohenlohe, P. A., Jaffé, R., Johannesson, K., Liggins, L., MacDonald, A. J., OrozcoterWengel, P., Reusch, T. B. H., Rodríguez-Correa, H., Russo, I.-R. M., Ryman, N., & Vernesi, C. (2020). Post-2020 goals overlook genetic diversity. *Science*, *367*(6482), 1083–1085.

Laikre, L., Lundmark, C., Jansson, E., Wennerström, L., Edman, M., & Sandström, A. (2016). Lack of recognition of genetic biodiversity: International policy and its implementation in Baltic Sea marine protected areas. *Ambio*, *45*(6), 661–680.

Lappe, A. K. R., Torales-Sanchez, D., Fuentes, A. B. G., & Caratozzolo, P. (2021). Work in Progress: Addressing Barriers for Women in STEM in Mexico. In *2021 IEEE Global Engineering Education Conference (EDUCON)* (pp. 1600-1604). IEEE.

Likert, R. (1932). A technique for the measurement of attitudes. *Archives of psychology*.

Mittermeier, R.A., Turner, W.R., Larsen, F.W., Brooks, T.M., Gascon, C. (2011). Global Biodiversity Conservation: The Critical Role of Hotspots. In: Zachos, F., Habel, J. (eds) Biodiversity Hotspots. Springer, Berlin, Heidelberg.

Moraes AM, Lima JDS, Alexandre BR, Ayala-Burbano PA, de Freitas PD, Ruiz-Miranda CR, Miyaki CY. (2023). Genetic Management Applied to Conservation of Reduced and Fragmented Wild Populations. In: Pedro M. Galetti Jr. (Ed). Conservation Genetics in the Neotropics. Springer, Cham. Pp.227-249.

Mosquera, D. M. V., & Rodríguez, J. A. V. (2023). Case of Policies for Gender Equality in Latin America Andean Community of Nations–CAN (Colombia, Bolivia, Peru, Ecuador). In *Economy, Gender and Academy: A Pending Conversation* (pp. 77-92). Emerald Publishing Limited.

Mukhopadhyay, U. (2023). Disparities in Female Labour Force Participation in South Asia and Latin America: A Review. *Review of Economics* 74(3): 265-288.

Myers, N., Mittermeier, R. A., Mittermeier, C. G., da Fonseca, G. A., & Kent, J. (2000). Biodiversity hotspots for conservation priorities. *Nature*, *403*(6772), 853–858.

Oliveira-Miranda, M. A, Martino, A.M., De Oliveira-Miranda, R. M., Balboa, K & Aguilera, M. (2013). Conserving the Genetic Diversity of Plants in Austral and Neotropical America (ANA): A Metanalysis of Published Studies Using Samples of the Region. Bot. Rev. 79: 449-468.

Osorio-Delvalle C, Ojeda-Caicedo VV, Villa-Ramirez JL, Contreras-Ortiz SH. (2020). Participation of women in STEM higher education programs in Latin America: The issue of inequality. 18th LACCEI International Multi-Conference for Engineering, Education, and Technology: “Engineering, Integration, and Alliances for a Sustainable Development” “Hemispheric Cooperation for Competitiveness and Prosperity on a Knowledge-Based Economy”, July 27-31, 2020, Virtual Edition.

Pierson, J. C., Coates, D. J., Oostermeijer, J. G. B., Beissinger, S. R., Bragg, J. G., Sunnucks, P., Schumaker, N. H., & Young, A. G. (2016). Genetic factors in threatened species recovery plans on three continents. *Frontiers in Ecology and the Environment*, *14*(8), 433–440.

Ramirez, J.L., Rosas-Puchuri, U., Cañedo, R.M., Alfaro-Shigueto, J., Ayon, P., Zelada-Mázmela, E., Siccha-Ramirez, R. & Velez-Zuazo, X. (2020). DNA barcoding in the Southeast Pacific marine realm: Low coverage and geographic representation despite high diversity. *PloS One* 15(12): e0244323.

Rodríguez-Clark, K. M., Oliveira-Miranda, M. A., Aguilera Meneses, M., Martino, Á., Méndez, M. A., Miyaki, C., Montiel-Villalobos, M. G., De Oliveira-Miranda, R. M., Poulin, E., Ruzzante, D., & Solé-Cava, A. (2015). Finding the “conservation” in Conservation Genetics - Progress in Latin America. *Journal of Heredity*, *106*(S1), 423–427.

Rojas Bonzi, V., Eguren, A., Nuñez-Regueiro, M. M., Marquez García, M., Hernandez, F., Clavijo, C., & Rodriguez Jorquera, I. (2018). Desafíos y oportunidades para conectar la investigación y la práctica de la conservación en el Cono Sur de América. *Paraquaria Natural*, *6*(August), 18–25.

Ruelas Inzunza, E., Cockle, K. L., Núñez Montellano, M. G., Fontana, C. S., Cuatianquiz Lima, C., Echeverry-Galvis, M. A., ... & Miño, C. I. (2023). How to include and recognize the work of ornithologists based in the Neotropics: Fourteen actions for Ornithological Applications, Ornithology, and other global-scope journals. *Ornithological Applications*, *125*(1), duac047.

Sandström A, Lundmark C, Jansson E, Edman M, Laikre L. (2016). Assessment of management practices regarding genetic biodiversity in Baltic Sea marine protected areas. *Biodiversity and Conservation* 25:1187–1205.

Sandström, A., Lundmark, C., Andersson, K., Johannesson, K., & Laikre, L. (2019). Understanding and bridging the conservation‐genetics gap in marine conservation. *Conservation Biology*, 33(3), 725.

Shafer, A. B., Wolf, J. B., Alves, P. C., Bergström, L., Bruford, M. W., Brännström, I., ... & Zieliński, P. (2015). Genomics and the challenging translation into conservation practice. *Trends in ecology & evolution*, *30*(2), 78-87.

Soares, L., Cockle, K. L., Ruelas Inzunza, E., Ibarra, J. T., Miño, C. I., Zuluaga, S., ... & Martins, P. V. R. (2023). Neotropical ornithology: Reckoning with historical assumptions, removing systemic barriers, and reimagining the future. *Ornithological Applications*, *125*(1), duac046.

Taft, H. R., McCoskey, D. N., Miller, J. M., Pearson, S. K., Coleman, M. A., Fletcher, N. K., Mittan, C. S., Meek, M. H., & Barbosa, S. (2020). Research–management partnerships: An opportunity to integrate genetics in conservation actions. *Conservation Science and Practice*, *2*(9), 1–8.

Taylor, H., Dussex, N., & van Heezik, Y. (2017). Bridging the conservation genetics gap by identifying barriers to implementation for conservation practitioners. *Global Ecology and Conservation*, *10*, 231–242.

Torres-Florez, J. P., Johnson, W. E., Nery, M. F., Eizirik, E., Oliveira-Miranda, M. A., & Galetti, P. M. (2018). The coming of age of conservation genetics in Latin America: what has been achieved and what needs to be done. *Conservation Genetics*, *19*(1), 0.

UNEP-WCMC United Nations Environmental Program - World Conservation Monitoring Centre (2016). The State of Biodiversity in Latin America and the Caribbean: A mid-term review of progress towards the Aichi Biodiversity Targets. UNEP-WCMC, Cambridge, UK.

UNESCO Institute for Statistics (UIS). (2021). Researchers in R&D (per million people). Data as of September 2021 (Tech. Rep.SP.POP.SCIE.RD.P6). Retrieved from https://data.worldbank.org/indicator/SP.POP.SCIE.RD.P6

Willi, Y., Kristensen, T. N., Sgro, C. M., Weeks, A. R., Ørsted, M., & Hoffmann, A. A. (2022). Conservation genetics as a management tool: The five best-supported paradigms to assist the management of threatened species. *Proceedings of the National Academy of Sciences of the United States of America*, *119*(1), 1–10.

Willoughby, J. R., Sundaram, M., Wijayawardena, B. K., Kimble, S. J., Ji, Y., Fernandez, N. B., ... & DeWoody, J. A. (2015). The reduction of genetic diversity in threatened vertebrates and new recommendations regarding IUCN conservation rankings. *Biological Conservation*, 191, 495-503.

World Economic Forum. (2023). Global Gender Gap Index. Published 20 June 2023. https://www.weforum.org/publications/global-gender-gap-report-2023/in-full/benchmarking-gender-gaps-2023/
