# Supplementary material for: Understanding the conservation-genetics gap in Latin America: challenges and opportunities to integrate genetics into conservation practices
Source: Front Genet. 2024 Jul 8;15:1425531. doi: 10.3389/fgene.2024.1425531 (PMC11261212; doi:10.3389/fgene.2024.1425531)
Supplement: Supplementary file 2 [file DataSheet3.docx]

**Supplementary Material**

**Appendix III: Portuguese version of the survey.**

**Questionário sobre a relação entre pesquisa genética e manejo para a conservação na América Latina**

O Capítulo Cone Sul da América do Sul da Sociedade para a Biologia da Conservação (SCB) está

realizando um estudo para investigar a relação entre a pesquisa em genética e o manejo em conservação na América Latina. Colaboram a Rede Latino-Americana de Genética para a Conservação (ReGeneC), a Seção América Latina e Caribe da SCB e a Sociedade Mesoamericana para a Biologia e a Conservação.

Nosso objetivo é identificar lacunas e oportunidades para a colaboração entre pesquisadores(as) em genética e profissionais responsáveis pelo manejo de populações, espécies ou ecossistemas, implementando estudos genéticos que possam orientar e informar a gestão da conservação.

Este questionário tem como foco pessoas encarregadas do manejo em conservação: pessoas diretamente envolvidas com a conservação de uma área ou espécie, tanto no planejamento de estratégias de conservação (como planos de ação de espécies), supervisão do manejo ou monitoramento de espécies, ou a avaliação dos resultados destas ações. Este trabalho de gestão da conservação deve ser atual ou recente (nos últimos 5 anos). Pesquisadores(as) sem experiência prática em manejo para a conservação não devem responder este questionário.

Se você realiza ou ajuda a realizar manejo para a conservação em uma ou mais áreas e/ou espécies, agradeceríamos muito por responder este questionário, que dura em torno de 15 minutos. Nenhuma resposta é obrigatória. Todas as suas respostas serão mantidas em anonimato e serão utilizadas apenas para este estudo.

Para mais informações, por favor contacte a responsável pelo estudo: Constanza Napolitano, acadêmica do Departamento de Ciências Biológicas e Biodiversidade da Universidade de Los Lagos (Chile), no e-mail: [encuesta.manejo.conservacion@gmail.com](mailto:encuesta.manejo.conservacion@gmail.com)

# INFORMAÇÃO DO(A) RESPONDENTE

## 1.1 Como este questionário chegou até você?

*Selecciona todos los que correspondan.*


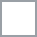
 Capítulo Cone Sul da América do Sul da Sociedade para a Biologia da Conservação (SCB)
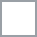
 Rede Latino-americana de Genética para a Conservação (ReGeneC)


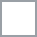
 Seção América Latina e Caribe (LACA) da Sociedade para a Biologia da Conservação
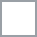
 Sociedade Mesoamericana para a Biologia e a Conservação (SMBC)


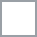
 Contato direto de um(a) colega ou amigo(a). Por favor, indicar nome da pessoa na opção “Outro”


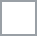
 Outro:

## 1.2 Qual a sua nacionalidade?

## 1.3 Em que país você vive atualmente?

## 1.4 Com qual gênero você se identifica?

### Marca solo um óvalo.


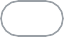
 Feminino
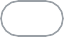
 Masculino
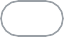
 Outro


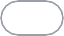
 Prefiro não declarar

## 1.5 Qual a sua idade? (coloque apenas números em anos)

1. 1.6 Em qual tipo de organização você realiza seu trabalho de manejo em conservação? (Se o seu trabalho de manejo é realizado em mais de um tipo de organização, responda aquela à que você dedica mais tempo).

### Marca solo um óvalo.

Agência governamental

Organização não-governamental (ONG) / Organização da sociedade civil (OSC)

Instituição acadêmica / de pesquisa

Organização concessionária de área de manejo (titular de concessão)

Outro:

## 1.7 Qual seu papel/função principal na organização?

### Marca solo um óvalo.


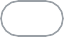
 Pesquisador(a) em biologia (em campo e/ou laboratório)
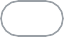
 Gestor(a) de manejo de recursos naturais


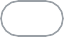
 Educador(a)


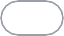
 Analista (tomada de decisões relacionadas a políticas públicas, normativas/legislação, planejamento estratégico)


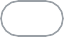
 Outro:

## 1.8 Qual sua posição na hierarquia da organização?

### Marca solo un óvalo.

Chefe(a)/Diretor(a) Intermediário(a)/Pesquisador(a) Nível operacional/Estudante

Outro:

# ÁREA/ESPÉCIE DE MANEJO

## 2.1 Em qual país está a área/espécie que você maneja? (Se estiver em mais de um país, assinale o principal)

## 2.2 Sua área/espécie de manejo encontra-se principalmente em ambiente:

### Marca solo un óvalo.

Terrestre Marinho Dulcícola

Outro:

## 2.3 Seu trabalho de manejo para a conservação em sua área/espécie é principalmente:

### Marca solo um óvalo.

In situ (no hábitat natural das espécies)

Ex situ (fora do hábitat natural das espécies) Ambas igualmente

Outro:

## 2.4 Em uma escala de 1 (nada importante) a 5 (muito importante), como você qualificaria as seguintes preocupações em sua área/espécie de manejo?

*Marca solo un óvalo por fila.*

1 2 3 4 5

Avaliar características da história de vida
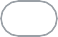


Avaliar o tamanho das populações Demarcar ou definir populações Detetar/ prevenir a hibridização

Avaliar a endogamia ou o grau de parentesco entre indivíduos

Cadastrar ou inventariar as espécies

Manter a conectividade entre populações ou identificar corredores

Identificar Unidades de Manejo

## 2.5 Já ocorreu algum dos seguintes casos com sua área/espécie?

*Marca solo um óvalo por fila.*

Sí No

Considerei usar estudo genéticos para fins de manejo

Colaborei com, ou contratei alguém para que fizesse um estudo genético

Usei resultados de estudos genéticos publicados por outra pessoa

Realizei um inventário de biodiversidade ou identifiquei espécies com DNA barcoding
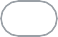
 ou DNA ambiental (eDNA)

# ESTUDOS GENÉTICOS NA ÁREA/ESPÉCIE DE MANEJO

## 3.1 Em uma escala de 1 (nada útil) a 5 (extremamente útil), como você qualificaria a utilidad dos estudos genéticos para os seguintes objetivos?

*Marca solo un óvalo por fila.*

1 2 3 4 5

Levantar informações básicas à respeito da área/espécie de manejo (ex. censo populacional ou composição de espécies)

Orientar/informar as ações de manejo Orientar/informar a proteção ou ação legal
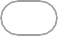
 Avaliar a efetividade de ações de manejo

## 3.2 Se estivesse interessado(a) em realizar um estudo genético em sua área/espécie de manejo, saberia como começar?

### Marca solo um óvalo.


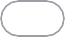
 Sim
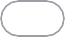
 Não

## 3.3 Você já realizou/utilizou um estudo genético em sua área/espécie de manejo? (Realizado você mesmo(a), ou delegado a outras pessoas, ou usado dados de estudos anteriores) (Se j realizou/utilizou mais de um estudo, refira-se ao que considere mais importante).

### Marca solo um óvalo.


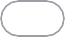
 Sim, realizei o estudo eu mesmo(a) (sozinho(a) ou em colaboração)
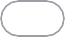
 Sim, deleguei o estudo a outras pessoas


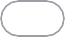
 Sim, utilizei resultados genéticos anteriores publicados por outras pessoas
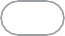
 Não


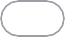
 Não sei

## 3.4 Se você já realizou/utilizou um estudo genético: quem elaborou a pergunta abordada pelo estudo? (Se já realizou/utilizou mais de um, refira-se ao que considere mais importante)

### Marca solo um óvalo.

Você ou alguém da sua organização

De forma conjunta com colaboradores(as) externos(as) à sua organização Um grupo externo à sua organização

Não se aplica (não realizei/utilizei estudo genético)

Outro:

## 3.5 Se você realizou/utilizou um estudo genético, quais grupos taxonômicos foram estudados (Marque todos os que correspondam)

*Selecciona todos los que correspondan.*


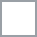
 Animais
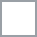
 Plantas
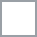
 Fungos


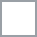
 Microorganismos (bactéria, arquea, protista)


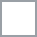
 Não se aplica (não realizei/utilizei estudo genético)


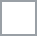
 Outro:

## 3.6 Mencione, se houve, a principal espécie que era o objetivo do estudo.

1. 3.7 Se você já realizou/utilizou um estudo genético em sua área/espécie de manejo, qual d seguintes situações corresponde ao seu caso? (Se realizou/utilizou mais de um estudo, refira se ao que considere mais importante):

### Marca solo um óvalo.


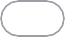
 O estudo ainda está em andamento, portanto os resultados não foram entregues/ não estão disponíveis para você


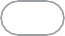
 O estudo foi concluído e os resultados não foram entregues/ não estão disponíveis para você
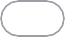
 O estudo foi concluído e os resultados foram entregues/ estão disponíveis para você


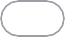
 Não se aplica (não realizei/utilizei estudo genético)

## 3.8 Se você realizou/utilizou um estudo genético e os resultados estiverem disponíveis ou foram entregues, em uma escala de 1 (complemente em desacordo) a 5 (completamente de acordo), qualifique as seguintes frases para o caso do estudo realizado/utilizado:

*Marca solo um óvalo por fila.*

Os resultados foram muito técnicos

Os resultados não corresponderam ou não foram relevantes para seus objetivos de manejo

Os resultados ajudaram a orientar/informar decisões de manejo

A experiência levou a outras colaborações ou pesquisas depois do estudo

A experiência ajudou a inspirar novos projetos

Não se aplica (não realizei/utilizei estudo genético, ou os resultados no estão disponíveis ou não foram entregues.

1 2 3 4 5

## 3.9 Se você já realizou/utilizou um estudo genético, classifique as seguintes condições de acordo com a sua presença ou ausência e se isto influenciou ou não a sua decisão de realizar o estudo:

*Marca solo um óvalo por fila.*

Sim, tivemos e isto influenciou nossa decisão

Acesso a um laboratório de genética

Sim, tivemos e isto NÃO influenciou nossa decisão

NÃO tivemos e isso SIM influenciou nossa decisão

NÃO tivemos e isso NÃO influenciou nossa decisão

Acesso a financiamento

Acesso a artigos atuais em revistas científicas

Acesso a artigos antigos em revistas científicas

Acesso a amostras

Confiança na aplicabilidade dos resultados às decisões de manejo

Confiança nas perguntas que podem ser respondidas pelo estudo

Pessoal apto a realizar trabalho de campo (você inclusive)

Pessoal apto a realizar trabalho de laboratório (você inclusive)

Alguém que possa orientar o

planejamento de um estudo genético (você inclusive)

Não se aplica (não realizei/utilizei estudo genético.

## 3.10 Se você NÃO realizou/utilizou um estudo genético, classifique as seguintes condições d acordo com presença ou ausência e se isso influenciou a sua decisão de Não realizar o estudo:

*Marca solo um óvalo por fila.*

SIM, tivemos e isto influenciou nossa decisão

Acesso à um laboratório de genética

Acesso a financiamento

Acesso a artigos atuais em revistas científicas

Acesso a artigos antigos em revistas científicas

Acesso a amostras

SIM, tivemos e isto NÃO influenciou nossa decisão

NÃO tivemos e isso SIM influenciou nossa decisão

NÃO tivemos e isso NÃO influenciou nossa decisão

Confiança na aplicabilidade dos resultados às decisões de manejo

Confiança nas perguntas que podem ser respondidas pelo estudo

Pessoal apto a realizar trabalho de campo (você inclusive)

Pessoal apto a realizar trabalho de laboratório (você inclusive)

Alguém que possa orientar o planejamento de um estudo genético (você inclusive)

Não se aplica (não realizei/utilizei estudo genético.

## 3.11 Na ausência de restrições, em uma escala de 1 (não usaria) a 5 (definitivamente usaria quão provável seria você realizar/usar estudos genéticos em sua área/espécie para os seguintes objetivos?

*Marca solo um óvalo por fila.*

1 2 3 4 5

Avaliação de conectividade

Avaliação de endogamia

Avaliação de características de história de vida

Avaliação do tamanho das populações Deteção de hibridização

Deteção de estrutura populacional

Deteção de espécies (ex. DNA ambiental)
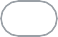
 Identificação de Unidades de Manejo

# COLABORAÇÃO COM OUTROS GRUPOS

## 4.1 Se você tivesse todos os recursos necessários e estivesse pronto(a) para realizar um estudo genético em sua área/espécie de manejo, em uma escala de 1 (nada provável a 5 (extremamente provável), quão provável seria que você contatasse os seguintes grupos para realizar o estudo?

*Marca solo um óvalo por fila.*

Laboratório acadêmico

Outra pessoa, unidade ou departamento dentro da sua organização

Agência governamental

Organização não-governamental (ONG)

/organização da sociedade civil (OSC) Empresa de consultoria privada

Não contataria a nenhum grupo e realizaria você mesmo(a)

1 2 3 4 5

## 4.2 Alguma vez já te contataram com uma proposta para realizar um estudo genético em s área/espécie de manejo?

### Marca solo um óvalo.


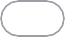
 Sim
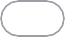
 Não

## 4.3 Se te contataram para realizar um estudo genético, que tipo de grupo te contatou? (Marque todas as respostas que correspondam)

*Selecciona todos los que correspondan.*


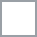
 Outra pessoa/unidade na sua organização
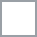
 Empresa de consultoria privada


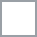
 Laboratório acadêmico externo à sua organização


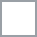
 Organização governamental externa à sua organização


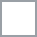
 Organização não-governamental (ONG) / organização da sociedade civil externa à sua organização


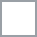
 Não se aplica (Não me contataram)

## 4.4 Se te contataram para realizar um estudo genético, de que país é o grupo que

te contatou? (Se mais de um grupo te contatou, refira-se àquele que considere mais importante

## 4.5 Se te contataram para realizar um estudo genético, de que gênero é a pessoa que lidera equipe que te contatou?

### Marca solo um óvalo.


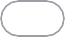
 Feminino
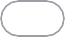
 Masculino
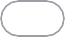
 Outro


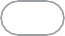
 Prefere não declarar
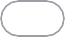
 Não sabe

## 4.6 Se um(a) acadêmico(a) geneticista te oferecesse ajuda para elaborar e/ou realizar um estudo genético em sua área/espécie de manejo, você aceitaria?

### Marca solo um óvalo.


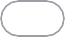
 Sim
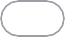
 Não
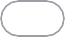
 Talvez

## 4.7 A respeito da sua resposta anterior, por que aceitaria ou não?

1. 4.8 Se o serviço de uma empresa de consultoria não acadêmica estivesse disponível para ajudar a elaborar e implementar um estudo genético em sua área/espécie de manejo, você pediria ajuda?

### Marca solo um óvalo.


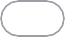
 Sim
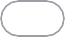
 Não
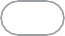
 Talvez

1. 4.9 A respeito a sua resposta anterior, por que pediria ajuda ou não pediria?

# COMENTÁRIOS FINAIS

1. 5.1 Tem alguma informação que você gostaria de compartilhar que possa ajudar a aproxima geneticistas preocupados com conservação e os gestores de manejo para a conservação? Este espaço é para que você colocar uma frase que possa ser publicada em um artigo científico para ajudar a entender sua perspetiva. Por favor, limite sua resposta a 2 ou 3 frases Se quiser que seu nome seja associado à frase, por favor, indique seu nome ao final; caso contrário, será mencionado como uma frase anônima.
2. 5.2 Há algo mais que gostaria que soubéssemos sobre a aplicação ou o potencial dos estudos genéticos na sua área/espécie?
3. Muito obrigado por participar desta pesquisa! Agradecemos muito sua ajuda. Se quiser nos autorizar a realizar possíveis contatos futuros por e-mail, por favor, indique aqui:

### Marca solo um óvalo.


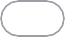
 Sim, autorizo ser contactado(a)
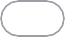
 Não, prefiro não ser contactado(a)

## Se você nos autorizar, digite seu endereço de e-mail aqui:
