# Supplementary material for: Understanding the conservation-genetics gap in Latin America: challenges and opportunities to integrate genetics into conservation practices
Source: Front Genet. 2024 Jul 8;15:1425531. doi: 10.3389/fgene.2024.1425531 (PMC11261212; doi:10.3389/fgene.2024.1425531)
Supplement: Supplementary file 3 [file DataSheet5.docx]

**Supplementary Material**

**Appendix V: Portuguese version of the manuscript**

**Compreendendo a lacuna entre conservação e genética na América Latina: Desafios e oportunidades para integrar a genética às práticas de conservação**

Constanza Napolitano*^1,2,3^, Cristhian Clavijo^4^, Viviana Rojas-Bonzi^5,6^, Carolina I Miño^7^, José F González-Maya^8^, Nadia Bou^9^, Alan Giraldo^10^, Angela Martino^11^, Cristina Yumi Miyaki^12^, Luis F Aguirre^13^, Andrea Cosacov^14^, Yoamel Milián-García^15^, Laura Prosdocimi^16^, O Eric Ramírez-Bravo^17^, Luis Antonio Tovar^18^, Ximena Velez-Zuazo^19^, Mercedes Barrios^20^, Bernal Herrera-Fernández^21^, María G Montiel-Villalobos^22^, María A Oliveira-Miranda^23^, Monique Pool^24^, Alonso Santos-Murgas^25^, M Claudia Segovia-Salcedo^26^, Felipe Cecchi^27^, Armando J Dans^28^, Nelanie Dilchand^29^, Sergio MQ Lima^30^, María Caridad Novas^31^, Karla Pelz-Serrano^32^, Nina Pougy^33^, Iris Rodríguez^34^, Liesbeth van der Meer^35^, Galo Zapata-Ríos^36^

^1^Departamento de Ciencias Biológicas y Biodiversidad, Universidad de Los Lagos, Osorno, Chile.
^2^Institute of Ecology and Biodiversity, Concepción, Chile.
^3^Cape Horn International Center, Puerto Williams, Chile.
^4^Vida Silvestre Uruguay, Montevideo, Uruguay.
^5^Instituto de Investigación Biológica del Paraguay, Asuncion, Paraguay.
^6^Wildlife Ecology and Conservation Department, University of Florida, Gainesville, USA.
^7^Laboratorio de Genética Evolutiva - LGE, Instituto de Biología Subtropical - IBS, Consejo Nacional de Investigaciones Científicas y Técnicas - CONICET, Universidad Nacional de Misiones (UNaM), Posadas, Misiones, Argentina.
^8^Área de Biología de la Conservación, Departamento de Ciencias Ambientales, DCBS, Universidad Autónoma Metropolitana Unidad Lerma, Lerma de Villada, México & ProCAT Colombia/Costa Rica, Bogotá, Colombia.
^9^Departamento de Biodiversidad y Genética, Instituto de Investigaciones Biológicas Clemente Estable, Montevideo, Uruguay.
^10^Grupo de Investigación en Ecología Animal, Departamento de Biología, Facultad de Ciencias Naturales y Exactas, Universidad del Valle, Cali, Colombia.
^11^Centro de Investigaciones en Ecología y Zonas Aridas, Universidad Nacional Experimental Francisco de Miranda, Coro, Falcón, Venezuela.
^12^Departamento de Genética e Biologia Evolutiva, Instituto de Biociências, Universidade de São Paulo, São Paulo, Brazil.
^13^Centro de Biodiversidad y Genética, Universidad Mayor de San Simón, Cochabamba, Bolivia.
^14^Laboratorio de Ecología Evolutiva y Biología Floral, Instituto Multidisciplinario de Biología Vegetal, CONICET-Universidad Nacional de Córdoba, Córdoba, Argentina.
^15^Department of Integrative Biology, University of Guelph, Guelph, Canada.
^16^Laboratorio de Ecología, Comportamiento y Mamíferos Marinos (LECyMM), Museo Argentino de Ciencias Naturales (MACN-CONICET), Buenos Aires, Argentina.
^17^Centro de Agroecología, Instituto de Ciencias, Benemérita Universidad Autónoma de Puebla, Eco campus Valsequillo, San Pedro Zacachimalpa, México.
^18^Facultad de Ciencias Forestales, Universidad Nacional Agraria La Molina, Lima, Perú.
^19^Smithsonian National Zoological Park and Conservation Biology Institute, Washington DC, USA.
^20^Centro de Datos para la Conservación, Centro de Estudios Conservacionistas, Universidad de San Carlos de Guatemala, Ciudad de Guatemala, Guatemala.
^21^Instituto Internacional de Conservación y Manejo de Vida Silvestre (ICOMVIS), Universidad Nacional, Heredia, Costa Rica.
^22^Red Latinoamericana de Genética para la Conservación (ReGeneC), La Paz, BCS, México.
^23^Red Latinoamericana de Genética para la Conservación (ReGeneC), Caracas, Venezuela.
^24^Green Heritage Fund Suriname, Paramaribo, Suriname.
^25^Departamento de Zoología, Facultad de Ciencias Naturales Exactas y Tecnología, Universidad de Panamá, Ciudad de Panamá. Estación Científica Coiba AIP, Ciudad del Saber, Panamá.
^26^Departamento de Ciencias de la Vida y la Agricultura, Universidad de las Fuerzas Armadas -ESPE, Sangolquí, Ecuador.
^27^Grupo Antropología de la Conservación, Universidad de Los Lagos, Osorno, Chile.
^28^Departamento de Ciencias Ambientales y Producción Sostenible, Universidad URACCAN, Bluefields, Nicaragua.
^29^Aquatic and Terrestrial Pioneers Consulting Services, Georgetown, Guyana.
^30^Departamento de Botânica e Zoologia, Universidade Federal do Rio Grande do Norte, Natal, Brazil.
^31^División de Conservación, Departamento de Botánica, Jardín Botánico Nacional Dr. Rafael María Moscoso, Santo Domingo, República Dominicana.
^32^Departamento de Ciencias Ambientales, División de Ciencias Biológicas y de la Salud, Universidad Autónoma Metropolitana Unidad Lerma, Lerma, México.
^33^Departamento de Desenvolvimento Científico, Museu do Amanhã, Instituto de Desenvolvimento e Gestão - IDG, Rio de Janeiro, Brazil.
^34^Escuela de Biología, Universidad Nacional Autónoma de Honduras, Tegucigalpa, Honduras.
^35^Oceana Chile, Santiago, Chile.
^36^Wildlife Conservation Society - Ecuador Program, Quito, Ecuador.

*Autor correspondente: constanza.napolitano@ulagos.cl

**Resumo**

A integração de dados genéticos nas decisões de gestão da conservação é uma tarefa desafiadora que exige parcerias sólidas entre pesquisadores e gestores. A conservação na América Latina é de relevância crucial para o planeta, dados os altos níveis de biodiversidade e a presença de hotspots nesta região. Realizamos uma pesquisa em toda a América Latina para identificar lacunas e oportunidades de colaboração entre pesquisadores de genética e gestores de conservação. Nosso objetivo foi de entender melhor os pontos de vista dos gestores de conservação e como a pesquisa genética poderia ajudar os profissionais de conservação a atingir suas metas, implementando avaliações genéticas que pudessem embasar efetivamente as práticas de conservação. Distribuímos uma enquete on-line por meio de quatro organizações colaboradoras regionais e 32 pontos focais localizados em 20 países da América Latina. Os entrevistados-alvo eram gestores de conservação de espécies ou de áreas na América Latina. Obtivemos um total de 468 questionários respondidos de 21 países latino-americanos. A maioria dos entrevistados (44%) pertencia a alguma instituição acadêmica ou de pesquisa, enquanto os não acadêmicos eram principalmente de instituições não governamentais (30%) e agências governamentais (25%). A maioria dos entrevistados (65%) realizou ou usou avaliações genéticas em sua área ou espécie gerenciada, seja sozinho, em parceria, contratando outra pessoa ou usando resultados publicados. Para a maioria desse grupo, os resultados genéticos foram relevantes para suas metas de gestão de conservação, ajudando a informar as decisões de gestão. Os entrevistados que não realizaram avaliações genéticas (35%) pertenciam principalmente ao grupo não acadêmico, e suas principais barreiras foram o acesso limitado a verbas, instalações de laboratórios de genética e pessoal treinado para planejar estudos e realizar trabalhos de laboratório. Com base nos resultados, descrevemos a situação atual e fornecemos um diagnóstico geral da falta de uso da genética na conservação na América Latina. Descrevemos o viés de gênero, a cooperação entre os acadêmicos e os gestores no planejamento de questões e projetos de conservação e a nacionalidade e residência dos gestores de conservação latino-americanos em relação aos países onde trabalham. Discutimos as oportunidades de colaborações na proposição de perguntas de pesquisa e no desenvolvimento de estudos com base nas necessidades dos profissionais de conservação. Oferecemos recomendações para superar barreiras à integração de informações genéticas em ações de conservação e para o avanço de agendas que se ajustem às necessidades e realidades da região latino-americana altamente heterogênea, biodiversa e desafiadora.

**Palavras-chave**: práticas de gestão de conservação, gestores de conservação, geneticistas, parcerias, conhecimento local, espécies ameaçadas de extinção, áreas importantes para a conservação.

**Introdução**

Para responder à atual crise de biodiversidade provocada pelas mudanças globais induzidas pelo homem (Ceballos et al., 2020), é essencial integrar e transpor as evidências científicas em estruturas sociopolíticas, processos-chave de tomada de decisão e políticas públicas (Díaz et al. 2019). Essa missão urgente exige uma visão ampla e interdisciplinar de pesquisadores, gestores de conservação, tomadores de decisão, políticos, legisladores e todas as partes interessadas em geral, que devem colaborar para buscar metas e indicadores de pesquisa comuns e aplicar medidas para preservar a biodiversidade (Ferreira & Klütsch, 2021; Kershaw et al., 2022; Willi et al., 2022).

Entre os componentes da biodiversidade, a diversidade genética desempenha um papel crucial na resiliência do ecossistema, na sobrevivência das espécies e na adaptação (Hoban, et al., 2021a; Laikre et al., 2020; Taft et al., 2020; Díaz et al., 2019; Taylor et al., 2017). Os dados genéticos fornecem percepções exclusivas sobre o status e as tendências de conservação de espécies e populações (Willoughby et al., 2015; Pierson et al., 2016; Torres-Florez et al., 2018; Garner et al., 2020). No entanto, apesar de sua reconhecida importância, a incorporação de informações genéticas às estratégias e políticas de gestão da conservação continua sendo um desafio global (Laikre, 2010; Laikre et al. 2010, 2016; Shafer et al 2015; Cook & Sgrò, 2017, 2018; Sandström et al 2019; Taft et al., 2020; Hoban et al 2020, 2021b; Galetti, 2023), contribuindo para o que é conhecido como a "lacuna de aplicação da genética na conservação" (Taylor et al., 2017).

Estudos que analisaram a lacuna entre conservação e genética identificaram quatro grandes barreiras para incluir dados genéticos na gestão da conservação: i) a falta de um mecanismo eficiente para a transferência de conhecimento entre acadêmicos e partes interessadas (Hoban et al., 2013; Taylor et al., 2017; Britt et al. 2018; Taft et al, 2020; Klütsch & Laikre, 2021), ii) a falta de treinamento formal dos gestores para interpretar e avaliar dados genéticos (Cook & Sgrò, 2017, 2018; Haig et al., 2016; Taft et al., 2020; Taylor et al, 2017), iii) a percepção dos gestores sobre o custo e o financiamento necessário para realizar estudos genéticos (Klütsch & Laikre, 2021; Taylor et al., 2017) e iv) as estruturas burocráticas ou restrições das instituições governamentais relevantes (Rojas Bonzi et al., 2018; Klütsch & Laikre, 2021). Essas barreiras podem ser exacerbadas em regiões extensas, altamente diversificadas e culturalmente heterogêneas, como a América Latina. Essa região geográfica abriga uma proporção importante da biodiversidade total da Terra (≈40%; UNEP-WCMC 2016), inclui vários "hotspots" de biodiversidade (Myers et al., 2000, Mittermeier et al., 2011) e enfrenta uma degradação grave e crescente do habitat e um rápido declínio das populações selvagens (Josse & Fernandez, 2021; Myers et al., 2000; Rodríguez-Clark et al., 2015; UNEP-WCMC 2016).

As informações genéticas têm sido cada vez mais usadas na conservação na América Latina (Torres-Florez et al., 2018; Moraes et al., 2023), com o Brasil, o México e o Chile liderando o campo em termos de número de estudos publicados (Rodríguez-Clark et al., 2015). Esses estudos se concentram principalmente em espécies terrestres (Oliveira-Miranda et al., 2013; Torres-Florez et al., 2018), questões de genética populacional e diversidade genética, e na avaliação das consequências genéticas da superexploração e da perda de habitat (Torres-Florez et al., 2018). Embora vários desses artigos tenham incluído recomendações específicas para ações de conservação e gerenciamento, ainda não está claro até que ponto essas sugestões ou outras descobertas genéticas foram efetivamente integradas às estratégias de conservação, ações de gerenciamento e/ou políticas formais em toda a América Latina (Torres-Florez et al., 2018), principalmente em países com produção científica limitada. Esse entendimento é fundamental para desvendar os fatores que perpetuam a lacuna entre conservação e genética em toda a América Latina.

No presente estudo, nosso objetivo específico foi caracterizar a lacuna entre conservação e genética na América Latina, investigando de forma abrangente a relação entre a pesquisa genética e o manejo para conservação na região. Nosso objetivo foi identificar e compreender as lacunas que precisam ser preenchidas e os desafios e oportunidades enfrentados pela comunidade de conservação da América Latina para integrar a genética à prática de conservação. Baseamos nosso estudo naquele realizado por Taft et al. (2020), que exploraram as barreiras que impedem o uso de dados genéticos para a prática e a política de conservação, consultando profissionais de conservação de diversas instituições acadêmicas e governamentais (n=50). No entanto, suas descobertas são predominantemente provenientes de uma amostra com viés geográfico, principalmente dos Estados Unidos da América. Portanto, as percepções derivadas de Taft et al. (2020) não são diretamente transferíveis e não podem ser generalizadas para avaliar o uso eficaz de informações genéticas em práticas de gestão para conservação na região altamente heterogênea da América Latina.

Portanto, nossos objetivos são: i) identificar lacunas e oportunidades de colaboração entre pesquisadores e profissionais da área de genética com o objetivo de implementar avaliações genéticas (nos níveis de população, espécie e ecossistema) que possam informar a gestão da conservação na América Latina; e ii) entender como os pesquisadores e profissionais da área de conservação podem colaborar de forma eficaz e se envolver em parcerias frutíferas para atingir metas comuns de conservação na região. Avaliamos as principais barreiras e discutimos recomendações e perspectivas para a criação e o desenvolvimento de modo colaborativo de estudos especificamente adaptados para atender às necessidades dos profissionais da área de conservação, das partes interessadas e das comunidades locais, a fim de incentivar assim a integração efetiva da informação genética nas ações de conservação desta rica região em biodiversidade que é a América Latina.

**Métodos**

**Área de estudo**

Nós pretendíamos avaliar 20 países das Américas Austral e Neotropical (ANA), que inclui o México, a América Central, o Caribe (incluindo Guiana, Suriname, Guiana Francesa e as ilhas do Caribe) e a América do Sul. Essa área é politicamente conhecida como América Latina, cujos países possuem histórias e cenários socioeconômicos semelhantes ou comuns (Ceballos et al., 2009, Torres-Florez et al., 2018).

**Distribuição e aplicação da enquete**

Para selecionar nossos entrevistados-alvo, usamos a amostragem intencional, uma técnica implementada para identificar e selecionar indivíduos ou grupos de indivíduos que são especialmente conhecedores ou experientes em um determinado assunto (Cresswell & Plano, 2011). Para obter amostragem com tamanho representativo e uma distribuição homogênea do esforço de amostragem em toda a América Latina, trabalhamos com 32 pontos focais (63% mulheres; 38% homens) de cada um dos 20 países-alvo. Definimos um ponto focal como uma pessoa que trabalha com conservação em um país da América Latina e cuja função era identificar e contatar outros entrevistados-alvo, distribuir as enquetes e fazer o acompanhamento. O número de pontos focais por país (1-3) foi estabelecido de acordo com a população total do país e padronizado pelo número de pesquisadores por país (expresso por milhão) com base nos dados do Instituto de Estatística da UNESCO (UIS) no Banco de Dados do Banco Mundial (UNESCO, 2021). Além da amostragem direta por meio de pontos focais, também distribuímos a enquete usando listas de mala direta de grupos por meio das seguintes organizações colaboradoras: o Capítulo do Cone Sul da América do Sul da Sociedade de Biologia da Conservação (SCB), a Rede Latino-Americana de Genética da Conservação (ReGeneC), a Seção da América Latina e Caribe da SCB (LACA-SCB) e a Sociedade Mesoamericana de Biologia e Conservação.

**Público-alvo de entrevistados**

Os entrevistados-alvo eram gestores de conservação na América Latina, envolvidos direta e recentemente (ou seja, nos últimos 5 anos; 2017-2021) com a conservação de uma espécie ou área, seja por meio do planejamento de estratégias de conservação (ex. planos de ação para a conservação de espécies), supervisão de gestão, monitoramento de espécies ou avaliação do resultado de tais práticas. Os cientistas que não estão diretamente envolvidos no gerenciamento da conservação ou que não têm experiência em conservação na América Latina não foram incluídos na lista de respondentes.

**Desenho da enquete**

Adaptamos a enquete implementada por Taft et al. (2020) direcionando o nosso foco nos gestores e profissionais da América Latina. Assim, incluímos perguntas adicionais com base em aspectos específicos que queríamos abordar em um contexto regional, como a ciência com práticas coloniais ("parachute science"; Haelewaters et al., 2021; Liboiron, 2021; Asase et al., 2022; de Vos & Swartz, 2022, Horn et al., 2022) e a lacuna de gênero. A enquete foi traduzida para os quatro idiomas oficiais mais comuns da região geográfica visada: espanhol, português, inglês e holandês. Usamos o Google Forms (Google 2021) para implementar a enquete. Coletamos respostas durante um período de três meses (15 de agosto a 16 de novembro de 2021). A enquete de 15 minutos (Apêndice I, Material suplementar) não tinha perguntas obrigatórias e todas as respostas foram registradas como anônimas. As 35 perguntas foram agrupadas em quatro seções: (i) Dados demográficos do entrevistado, (ii) Área/espécie gerenciada, (iii) Tipo de estudo genético realizado e (iv) Parcerias com outros grupos de pesquisa e/ou profissionais da conservação. Foram usados diferentes formatos de perguntas: múltipla escolha (exclusivo/não exclusivo; n=25), escala Likert de cinco pontos (Likert, 1932; n=5) e perguntas abertas (n=5). Na primeira seção, perguntamos sobre nacionalidade, sexo, idade, local de trabalho, tipo de instituição de trabalho e função na instituição/organização. Na segunda seção, perguntamos sobre o local do estudo, o tipo de ambiente das áreas/espécies gerenciadas (ou seja, marinho, terrestre, de água doce) e os interesses específicos de pesquisa e/ou monitoramento relacionados à área/espécie gerenciada. Na terceira seção, perguntamos sobre a utilidade de diferentes tipos de avaliações genéticas, o interesse em realizar tais avaliações, informações sobre as avaliações realizadas e possíveis barreiras à sua realização/utilização. Na quarta seção, examinamos se o entrevistado precisou solicitar a ajuda de especialistas e se confiou em especialistas. Além disso, avaliamos a eficácia da distribuição da enquete e dos métodos de amostragem, incluindo uma pergunta sobre como a enquete chegou ao entrevistado. Registramos o esforço de amostragem (ou seja, o número de convites enviados) e medimos o sucesso da amostragem calculando a taxa de enquetes respondidas/esforço. Comparamos o sucesso da distribuição da enquete entre os pontos focais e as listas de mala direta das organizações para avaliar a eficácia de ambas as estratégias de distribuição da enquete.

Usamos estatísticas descritivas para resumir nossos resultados. A enquete incluiu um termo de consentimento livre e esclarecido e foi revisada e aprovada pelo Comitê de Ética do Instituto de Ecologia e Biodiversidade (IEB, Chile) (certificado de aprovação em 12 de agosto de 2021).

**Resultados**

**Distribuição da enquete e sucesso da amostragem**

Enviamos 2.196 convites a potenciais participantes da pesquisa, sendo esse o esforço total de amostragem. O sucesso em obter resposta variou de acordo com o método de distribuição da pesquisa: os convites enviados por meio de pontos focais resultaram em 383 respostas (de 1.344 convites enviados, com uma taxa de resposta de 28,5%, representando 81,8% das respostas), enquanto os enviados por meio de listas de mala direta de organizações colaboradoras produziram 79 respostas (de 852 convites, com uma taxa de resposta de 9,3%, representando 16,9% das respostas). Além disso, 0,85% (4 respostas) não especificaram como receberam a pesquisa, enquanto 0,43% (2 respostas) relataram ter recebido a pesquisa por outros meios, ou seja, de um colega ou de uma organização não governamental (ONG) local.

**Características demográficas dos entrevistados**

No total, recebemos 468 respostas de gestores de conservação que trabalham em 21 países da América Latina (Figura 1a). Cinquenta e cinco por cento dos respondentes se identificaram como homens, 43% como mulheres, 1% preferiu não declarar sua identidade de gênero e 1% não respondeu (0% Outros; Figura 4a). A maioria dos entrevistados tinha de 40 a 49 anos (35%), seguida por 30 a 39 (28%), 50 a 59 (21%), >60 (9%), <30 (5%) e 1% não respondeu a essa pergunta. ﻿A maioria dos entrevistados trabalhava em instituição acadêmica ou de pesquisa (44%), seguida por ONG (29%), agência governamental (26%) e truste fundiário para a conservação ("land trusts or conservancy"; 1%) (Figura 1b). Quanto à função dentro da respectiva organização, a maioria dos entrevistados declarou ser biólogo pesquisador (laboratório ou trabalho de campo; 53%), seguido por gestores de recursos naturais (16%), analistas (15%; tomadores de decisão em políticas públicas, legislação e/ou planejamento estratégico), outros (10%) e educadores (6%) (Figura 1c). Com relação ao cargo em sua organização, a maioria dos respondentes declarou ser de nível executivo de nível médio (pesquisador; 59%), seguido por diretor (33%) e funcionário operacional (7%) (0,4% não responderam).

A grande maioria dos entrevistados (97%) era de cidadãos da América Latina, seguidos por cidadãos da Europa (2%) e da América do Norte (1%). Com relação ao país de residência e à nacionalidade dos entrevistados, a maioria era residente (92%) ou nacional (92%) do país onde a área/espécie gerenciada está localizada, enquanto 87% eram nacionais e residentes. Uma porcentagem menor (2%, 10 entrevistados) não tinha nacionalidade nem era residente do país onde a área/espécie administrada está localizada. Desse último grupo, 1,4% (7 entrevistados) eram latino-americanos que trabalhavam em um país latino-americano diferente de seu país de origem, 0,4% eram norte-americanos que trabalhavam na América Latina (2 entrevistados) e 1 entrevistado era latino-americano que gerenciava uma área/espécie na América Latina, mas residia na América do Norte (0,2%).

**Área/espécie gerenciada e desafios identificados**

Os entrevistados gerenciam áreas ou espécies localizadas em ambientes terrestres (70%), marinhos (15%) e de água doce (9%), enquanto 6% gerenciam áreas ou espécies em vários tipos de ambiente (Figura 1d). A maioria dos entrevistados (69%) trabalha com manejo "in-situ" (ou seja, dentro do habitat natural da espécie), 24% trabalham tanto com manejo "in-situ" quanto "ex-situ" (ou seja, fora do habitat natural da espécie), enquanto apenas 7% dos entrevistados trabalham exclusivamente "ex-situ". Os principais grupos taxonômicos avaliados geneticamente foram animais (71%), seguidos por plantas (13%), microrganismos (Bactéria, Archaea, Protista) (1%), fungos (1%) e 14% dos entrevistados declararam ter estudado vários táxons.

Os entrevistados declararam que os desafios mais significativos em relação à sua área/espécie gerenciada eram: (i) Avaliar o tamanho da população (muito importante 67%, importante 21%); (ii) Manter a conectividade ou identificar corredores (muito importante 63%, importante 20%); (iii) Identificar unidades de manejo (muito importante 56%, importante 25%); (iv) Delinear populações (muito importante 50%, importante 25%); (v) Inventariar espécies (muito importante 45%, importante 21%); (vi) Avaliar características de história de vida (muito importante 40%, importante 26%); (vii) Avaliar consanguinidade ou parentesco de indivíduos (muito importante 32%, importante 18%) e (viii) Detectar/prevenir hibridização (muito importante 23%, importante 13%).

**Estudos genéticos na área/espécie gerenciada**

A maioria dos entrevistados (79%) indicou que já havia considerado o uso da genética para apoiar suas práticas de manejo, sendo que 67% afirmaram que saberiam como realizar uma avaliação genética em sua área/espécie manejada se tivessem interesse em fazê-lo. Além disso, 25% relataram ter realizado um inventário de biodiversidade ou identificado espécies com "DNA barcoding" ou técnicas de DNA ambiental.

Com relação à utilidade das informações genéticas, os entrevistados as consideraram extremamente úteis ou úteis para: (i) Informar ações de gestão (82%), (ii) Estabelecer informações de base sobre a área gerenciada (por exemplo, censo populacional ou composição de espécies) (82%), (iii) Avaliar a eficácia das ações de gestão (75%), ou (iv) Informar ações ou proteção legislativa (71%).

A maioria dos entrevistados (65%) declarou ter realizado ou usado uma avaliação genética em sua área/espécie gerenciada (Figura 2a). Desses, 51% realizaram a avaliação sozinhos ou em colaboração, 27% solicitaram/contrataram outra pessoa para realizar a avaliação e 22% usaram dados genéticos publicados (Figura 2b). A porcentagem de entrevistados que usaram/realizaram uma avaliação genética em sua área/espécie administrada foi maior entre os acadêmicos (70%) do que entre os não acadêmicos (60%). Por outro lado, a porcentagem de entrevistados que não usaram/realizaram uma avaliação genética foi maior entre os não acadêmicos (39%) do que entre os acadêmicos (29%; Figura 2c).

Para a proporção total de entrevistados de formação acadêmica ou não acadêmica que não realizaram ou não usaram uma avaliação genética em sua área/espécie administrada (35%; Figura 2a), as principais barreiras ou fatores limitantes que influenciaram sua decisão foram, em ordem de importância (i) Acesso limitado a financiamento (48%), (ii) Acesso limitado a um laboratório de genética (37%), (iii) Falta de ajuda ou orientação na elaboração de uma avaliação genética (33%), (iv) Falta de pessoal qualificado para realizar o trabalho de laboratório (33%), (v) Acesso limitado a amostras (24%), (vi) Falta de conhecimento das questões que podem ser abordadas (22%), (vii) Falta de confiança em relação à aplicabilidade dos resultados genéticos às decisões de gerenciamento (19%) e (viii) Falta de pessoal qualificado para realizar o trabalho de campo (18%) (Figura 3a).

Com relação à aplicabilidade dos resultados da avaliação genética, nossos resultados mostram que: (i) 41% dos acadêmicos e 46% dos não acadêmicos discordam completamente e discordam da afirmação "Os resultados foram muito técnicos", (ii) 82% dos acadêmicos e 79% dos não acadêmicos discordam completamente e discordam da afirmação "Os resultados não se aplicaram às nossas metas de gestão de conservação", e (iii) 71% dos acadêmicos e 71% dos não acadêmicos concordam completamente e concordam com a afirmação "Os resultados ajudaram a informar as decisões de gestão" (Figura 3b).

**Desenvolvimento de projetos de conservação em colaboração e disponibilidade / compartilhamento de dados**

Para descrever melhor a colaboração entre acadêmicos e profissionais na América Latina, perguntamos quem havia levantado inicialmente as questões abordadas pela avaliação genética. Nossos resultados mostram que, para a maioria dos entrevistados acadêmicos (58%), as questões de pesquisa foram levantadas por eles mesmos ou por alguém de seu grupo, em comparação com 25% dos não acadêmicos. Por outro lado, a maioria dos entrevistados não acadêmicos (46%) apresentou as perguntas em colaboração com parceiros de fora de sua organização, em comparação com 35% dos acadêmicos. Apenas 7% dos acadêmicos declararam que as perguntas surgiram de um grupo externo à sua organização, em comparação com uma proporção muito maior de não acadêmicos (29%).

Nossos resultados também mostram que 64% dos acadêmicos e 65% dos não acadêmicos declararam que as avaliações genéticas haviam sido concluídas e os resultados foram disponibilizados aos gestores no momento em que nossa enquete foi encerrada (16 de novembro de 2021), enquanto 11% dos acadêmicos e 7% dos não acadêmicos declararam que as avaliações haviam sido concluídas, mas os resultados não haviam sido disponibilizados aos gestores até aquele momento. Além disso, 25% dos acadêmicos e 28% dos entrevistados não acadêmicos declararam que as avaliações estavam em andamento e, portanto, os resultados não estavam disponíveis até o final de nossa pesquisa.

**Parcerias entre grupos ou organizações**

Para entender melhor as barreiras ou os desafios para iniciar e/ou manter parcerias entre gestores e pesquisadores da área de genética aplicada à conservação na América Latina, perguntamos aos gestores qual era a probabilidade de entrarem em contato com diferentes grupos como possíveis parceiros para realizar uma avaliação genética. Nossos resultados mostram que os entrevistados entrariam em contato, em ordem de importância, com (i) um laboratório acadêmico (Extremamente provável e provável, 88%), (ii) outra pessoa/unidade/filial em sua organização (Extremamente provável e provável, 68%), (iii) uma ONG (Extremamente provável e provável, 53%), (iv) uma organização governamental (Extremamente provável e provável, 41%; baixa probabilidade e improvável, 39%), (v) uma empresa de consultoria privada (baixa probabilidade e improvável, 55%), enquanto (vi) 71% (baixa probabilidade e improvável) não entrariam em contato com nenhum grupo e fariam isso sozinhos.

Além disso, nossos resultados mostram que, se um geneticista acadêmico oferecesse ajuda aos gestores para projetar e/ou conduzir uma avaliação genética, 80% deles estariam inclinados a aceitar, 19% talvez aceitassem e 1% não aceitaria. Se um serviço de consultoria não acadêmico estivesse disponível para ajudar os gestores a projetar e implementar uma avaliação genética, 51% dos entrevistados declararam que eles ou suas organizações talvez procurassem ajuda desse serviço, 32% procurariam ajuda e 17% não procurariam ajuda.

Mais da metade dos entrevistados (56%) declarou ter sido contatada para realizar uma avaliação genética em sua área/espécie administrada e que a solicitação veio dos seguintes grupos, em ordem de prevalência: (i) laboratório acadêmico externo (44%), (ii) outra pessoa/unidade em sua organização (22%), (iii) ONG externa (16%), (iv) organização governamental externa (13%) e (v) empresa de consultoria privada (5%). Uma maioria considerável desses contatos veio da própria América Latina (70%), seguida pela América do Norte (17%), Europa (8%), Outros (2%) e sem resposta (3%).

**Diferença de gênero**

Estávamos interessados em avaliar se havia diferença no número de homens e de mulheres na área de gestão de conservação na América Latina. Nossos resultados mostram que 63% das gestoras do sexo feminino ocupavam cargos executivos de nível médio (ou seja, pesquisadoras) e 28% ocupavam cargos de chefia ou de nível superior (ou seja, diretoras). Por outro lado, uma proporção maior de gestores do sexo masculino (37%) ocupou cargos de diretor executivo ou de nível superior, com 56% ocupando cargos executivos de nível médio (ou seja, pesquisador; Figura 4b).

Em relação aos entrevistados que foram contactados por algum grupo de pesquisa latino-americano para realizar uma avaliação genética, perguntamos sobre o gênero do líder desse grupo. A representação das mulheres em tais funções de liderança (40%) foi menor em comparação com os homens (51%), com 9% indicando incerteza, preferindo não declarar, ou outro (Figura 4b).

**Discussão**

Este estudo fornece um diagnóstico geral da situação atual da lacuna de aplicação da genética na conservação na América Latina. A partir dos resultados obtidos, oferecemos ideias e soluções para superar obstáculos e promover agendas que se ajustem às necessidades e realidades desse território altamente heterogêneo, biodiverso e desafiador (Figura 5).

O sucesso geral da amostragem foi muito maior por meio de pontos focais (28,8%) em comparação com o uso das listas de mala direta das organizações (9,3%). A distribuição por meio de pontos focais também facilitou o alcance da enquete aos respondentes na maioria dos casos (81,8%). A natureza personalizada dos convites enviados pelos pontos focais aos respondentes-alvo provavelmente promoveu um maior senso de confiança e relevância entre os participantes em potencial, aumentando assim a motivação para responder. Por outro lado, a abordagem mais impessoal de utilizar listas de mala direta das organizações pode ter resultado em níveis mais baixos de envolvimento pessoal e, consequentemente, em taxas de resposta reduzidas. Defendemos a adoção generalizada de pontos focais para a realização de enquetes on-line, já que eles podem estar mais cientes dos aspectos culturais e das redes locais em seus países, o que leva a um maior sucesso na obtenção de respostas.

Os entrevistados de nossa pesquisa pertenciam a diferentes tipos de organização, como instituições acadêmicas ou de pesquisa (44%), ONGs (29%) e instituições governamentais (26%) (Figura 1b). A composição demográfica em nosso estudo foi diferente daquela relatada por Taft et al. (2020), pois em nosso estudo a maior porcentagem de participantes veio de instituições governamentais (50%), seguida por instituições acadêmicas (24%) e ONGs (16%). Taft et al. (2020) descreveram uma tendência favorável entre os gestores ao uso da genética, bem como uma maior incidência de entrevistados que realizaram ou empregaram avaliações genéticas em comparação com nosso estudo, que descreveu uma proporção menor de estudos genéticos implementados por gestores. Portanto, as diferenças observadas entre nosso estudo e o de Taft et al. (2020) podem ser devido a um viés geográfico ou a diferenças nos tipos de instituições representadas em cada estudo. Em geral, as nações desenvolvidas alocam mais verba pública do que as nações em desenvolvimento para a pesquisa científica, incluindo a ciência da conservação (Gonzalez-Brambila et al., 2016). Considerando que os entrevistados da pesquisa de Taft et al. (2020) eram principalmente gestores de instituições governamentais sediadas nos EUA (50% da amostra), é provável que eles enfrentem menos restrições financeiras para realizar avaliações genéticas aplicadas à conservação em comparação com os gestores da América Latina. Como resultado, eles podem ter maior conscientização, treinamento e/ou experiência nessa área, em comparação com seus colegas latino-americanos.

Assim como Torres-Florez et al. (2018), a maioria dos entrevistados de nossa pesquisa trabalhou no ambiente terrestre (Figura 1d). Os entrevistados declararam ter realizado avaliações genéticas principalmente em animais, enquanto as plantas estavam sub-representadas em nossa amostra. No entanto, outro estudo de metadados relatou que, de todas as publicações revisadas de genética aplicada à conservação (1980-2010), 46% eram sobre plantas (Oliveira-Miranda et al., 2013).

As respostas à nossa enquete mostram que a maioria dos gestores de conservação na América Latina já havia realizado algum estudo genético ou usado dados genéticos em sua área/espécie gerenciada, e que metade deles trabalhava sozinho ou em colaboração (Figura 2a,b). De forma encorajadora, descobrimos que a maioria dos gestores de conservação não só está ciente da utilidade das avaliações genéticas para informar e avaliar as ações de gestão (Figura 3b), mas também já usou a genética e considera que tem o conhecimento técnico necessário para conduzir ou projetar uma avaliação genética por conta própria, sabe como aplicar ou interpretar dados genéticos publicados para atingir suas metas de conservação e/ou é capaz de encontrar ativamente um colaborador. Descobrimos que uma proporção maior de entrevistados acadêmicos havia realizado ou usado a genética para apoiar suas metas de conservação em comparação com os não acadêmicos; essa situação provavelmente reflete o maior acesso a pesquisas atualizadas, oportunidades de treinamento e redes de colaboração em ambientes acadêmicos, em comparação com os não acadêmicos. Notavelmente, nossos resultados contrastam com outros estudos que indicaram uma falta de conscientização e conhecimento sobre a utilidade de incluir dados genéticos em ações de conservação (Cook et al., 2013; Klütsch & Laikre, 2021). Esse cenário promissor destaca os possíveis benefícios da promoção de um sistema de rede de mentores dentro da comunidade de gestão da conservação (veja detalhes em "Diminuindo a lacuna: recomendações para melhorar”).

Um desafio importante na América Latina se concentra nos gestores que não realizaram ou não usaram nenhuma avaliação genética, o que representa um terço de nossa amostra (Figura 2a). Esses entrevistados indicaram que as principais barreiras para a implementação da genética são: acesso limitado a financiamento, acesso limitado a um laboratório de genética, falta de ajuda ou orientação na elaboração de uma avaliação genética e falta de pessoal qualificado para realizar o trabalho de laboratório (Figura 3a). Isso ecoa outros estudos que mostram que a maioria das limitações para a implementação efetiva da genética em ações de conservação na América Latina é operacional ou financeira, em vez de estar relacionada à falta de confiança na genética (por exemplo, Ramirez et al. 2020).

Além disso, e de acordo com pesquisas realizadas em todo o mundo (Haig et al., 2016; Taylor et al., 2017; Holderegger et al., 2019; Klütsch & Laikre, 2021), alguns entrevistados de nossa pesquisa consideram os resultados genéticos muito técnicos para serem interpretados (25% dos acadêmicos e 30% dos não acadêmicos) (Figura 3b). Isso não é surpreendente, já que a genética aplicada à conservação como campo de pesquisa exige o uso de termos técnicos e conceitos teóricos complexos, que necessariamente precisam de algum conhecimento básico e treinamento para serem compreendidos. Além disso, os conceitos de genética aplicada à conservação não estão comumente presentes no ensino médio ou em cursos técnicos pós Ensino Básico na América Latina (UNESCO, 2021).

Descobrimos que menos entrevistados não acadêmicos (25%) delinearam as questões de estudo abordadas pelas avaliações genéticas, em comparação com uma proporção maior de acadêmicos (58%) que o fizeram, seja individualmente ou em colaboração. Recomendamos que sejam feitos esforços para fortalecer os vínculos entre geneticistas e gestores, de modo que eles colaborem desde o início nas avaliações genéticas, definindo conjuntamente as perguntas do estudo e escolhendo a metodologia a ser aplicada, tudo em conexão com as necessidades dos gestores.

Uma alta proporção de entrevistados em nossa pesquisa, tanto acadêmicos (64%) quanto não acadêmicos (65%), declarou que, quando trabalharam em colaboração, os resultados genéticos estavam prontamente disponíveis para eles, em contraste com outros estudos que lamentam que os resultados obtidos com a genética permaneçam em grande parte no domínio acadêmico (Cook et al., 2013; Sandström et al., 2016; Taylor et al., 2017; Klütsch & Laikre, 2021). Esse resultado sinaliza um cenário positivo, uma vez que o compartilhamento de resultados científicos é a base para a transformação das informações em práticas de gestão eficazes e para a construção de parcerias de longo prazo confiáveis e bem-sucedidas.

Os resultados de nossa pesquisa também indicam que os gestores na América Latina provavelmente entrariam em contato com um laboratório acadêmico em vez de uma empresa de consultoria privada como parceiro para realizar uma avaliação genética. Além disso, a maioria dos gestores estaria inclinada a aceitar a ajuda de um geneticista acadêmico para projetar ou implementar uma avaliação genética, preferindo-os a um serviço de consultoria privado. Como o setor privado tende a ser mais caro, essas respostas poderiam destacar a falta de financiamento dos gestores na América Latina para pagar pelos serviços de empresas privadas (consequentemente, recorrendo a colaborações acadêmicas que geralmente dependem do financiamento garantido pelos pesquisadores) e/ou espelhar a alta reputação das instituições acadêmicas que são consideradas de excelência, com autonomia e socialmente responsáveis (Alzyoud et al., 2015). Isso enfatiza ainda mais a necessidade de promover maior financiamento para infraestrutura e treinamento de recursos humanos e pessoal em instituições acadêmicas e públicas que realizam pesquisas teóricas e aplicadas em genética aplicada à conservação na América Latina.

Uma proporção menor de entrevistados se autodeclarou mulher (43%), em comparação com os homens (55%) (Figura 4a). Diferentes cenários, que não são mutuamente excludentes, mas que estão além do escopo deste estudo, podem explicar essa diferença. As possíveis explicações são que elas refletem uma menor participação das mulheres na gestão da conservação na América Latina, ou que as mulheres estão de fato participando, mas não responderam à pesquisa (presumivelmente porque dedicam proporcionalmente mais tempo às tarefas domésticas e aos papéis de cuidadoras em comparação com os homens; Amarante et al., 2023; Mukhopadhyay, 2023). A diferença pode até refletir um viés não intencional na escolha dos entrevistados-alvo contatados por nossos 32 pontos focais [embora quase dois terços (63%) deles fossem do sexo feminino] ou uma sub-representação nas listas de mala direta das organizações colaboradoras (SCB, ReGeneC, LACA-SCB, Sociedade Mesoamericana de Biologia e Conservação). Entre as respondentes autodeclaradas mulheres, uma proporção menor (28%) ocupa cargos de chefia ou cargos de nível superior (ou seja, diretor) em suas organizações, em comparação com os homens (37%) (Figura 4b), e uma proporção menor de mulheres era líder de grupos de pesquisa que realizavam avaliações genéticas (40%) em comparação com os homens (51%) (Figura 4c). As lacunas e diferenças de gênero em ciência, tecnologia, engenharia e matemática (STEM) têm sido amplamente descritas e estudadas em todo o mundo (Fórum Econômico Mundial, 2023) e também na América Latina (Braverman-Bronstein et al., 2023; García-Holgado et al., 2020; Osorio-delvalle et al., 2020; Lappe et al., 2021). Nas últimas décadas, houve um progresso significativo na América Latina para melhorar a igualdade de gênero em STEM (García-Holgado et al., 2019; Mosquera & Rodriguez, 2023) e, embora alguns países estejam avançando bem no sentido de reduzir a lacuna de gênero (Fórum Econômico Mundial, 2023), ainda há muito espaço para melhorias.

Também exploramos a possível ocorrência e extensão da ciência com práticas coloniais ("parachute science") na América Latina, avaliando a cidadania e a residência dos entrevistados. Nossos resultados mostram um cenário promissor, indicando uma aparente capacitação local na gestão da conservação, uma vez que a grande maioria dos entrevistados era residente (92%) ou nacional (92%), ou ambos (87%), dos países onde a área/espécie gerenciada está localizada. Além disso, nossos resultados mostram que o treinamento e as capacidades locais para conduzir a gestão da conservação da biodiversidade local e as avaliações genéticas estão em vigor na região (Figuras 2a,b, 3b e ver Resultados). Nossas descobertas estão de acordo com as relatadas em uma revisão sobre genética aplicada à conservação de plantas (Oliveira-Miranda et al., 2013), que descreveu um aumento na participação de latino-americanos como primeiros autores (de 46% na década de 1990 para 71% em 2006-2010) e um aumento no número de publicações de autoria exclusiva de latino-americanos. Oliveira-Miranda et al. (2013) também observaram que o número de estudos em genética aplicada à conservação de plantas na América Latina com apenas co-autores estrangeiros era maior nos países entre o México e o Panamá, enquanto aqueles que incluíam apenas coautores locais eram maiores da Colômbia ao Brasil e à Bolívia.

O presente estudo foi projetado para priorizar a distribuição geográfica da amostragem nos países da América Latina, sendo um dos primeiros estudos a realizar uma ampla pesquisa em toda a região para descrever a lacuna de aplicação da genética na conservação (Ceballos et al., 2009; Taylor et al., 2017; Fabian et al., 2019; Taft et al., 2020). A representação geográfica de nossos resultados é mais ampla e maior do que a de estudos anteriores (Rojas Bonzi et al., 2018; Taft et al., 2020). Vale ressaltar que os resultados de nossa pesquisa não podem ser considerados como uma descrição da natureza complexa de toda a população de gestores de conservação da América Latina, porque a distribuição de nossa pesquisa não foi aleatória. Só podemos descrever e discutir nossos resultados com base em nossa amostra, considerando as informações dos entrevistados e reconhecendo possíveis vieses. Apesar dos nossos esforços para garantir uma amostra diversificada e equilibrada, visando respondentes de formação acadêmica e não acadêmica, bem como de várias afiliações organizacionais, reconhecemos as possíveis limitações. Os pontos focais podem ter entrado em contato involuntariamente com mais pessoas que compartilham o mesmo entusiasmo pelo uso de dados genéticos em esforços de conservação, embora um esforço claro para evitar esse viés tenha sido estabelecido desde o início. Portanto, como em Taft et al. (2020), nossa amostra pode ter uma representação maior de gestores que já tinham conhecimento, interesse ou treinamento em genética em comparação com a proporção real que eles podem representar na América Latina. Além disso, o uso de listas de mala direta de determinadas organizações também pode ter induzido a um viés de amostragem, por exemplo, a da Rede Latino-Americana de Genética Aplicada à Conservação (ReGeneC), embora sua representação específica em nossa amostra tenha sido muito menor (6,8%) em comparação com os pontos focais (81,8%). Nossa amostra também pode ter sido desequilibrada em outros aspectos, como o habitat ou ambiente em que os entrevistados trabalham (principalmente terrestre, 70%; Figura 1d), ou os grupos taxonômicos que estudam (principalmente animais, 71%). No entanto, acreditamos que nossa amostra reflete a diversidade de gestores de conservação presentes na América Latina em relação ao tipo de organização, função dos entrevistados e inclusão de gestores "in-situ" e "ex-situ" (consulte Resultados; Figura 1c). O viés de desejo social (ou seja, a tendência de alinhar as respostas com o que é percebido como socialmente aceitável ou politicamente correto) pode limitar a interpretação de nossos resultados, como ocorre em muitos outros estudos de pesquisa qualitativa que utilizam enquetes (Bergen & Labonté, 2020).

Ao interpretar e discutir nossos resultados, reconhecemos a alta heterogeneidade das realidades locais e das condições de trabalho que coexistem nos países latino-americanos em relação à pesquisa e ao desenvolvimento científico, capacidades de infraestrutura, treinamento especializado e acesso a informações científicas publicadas (UNESCO, 2021). Embora os países latino-americanos compartilhem histórias e cenários socioeconômicos semelhantes (Ceballos et al., 2009, Torres-Florez et al., 2018), há diferenças significativas na região. Por exemplo, o Brasil, a Argentina, o México e o Chile estão mais avançados na integração da genética ao manejo da conservação, em comparação com outros países latino-americanos (Oliveira-Miranda et al., 2013; Torres-Florez et al., 2018), devido a uma miríade de fatores, como diferenças nas oportunidades de financiamento e/ou na legislação, barreiras e as possíveis maneiras pelas quais a genética pode ser aplicada à conservação (por exemplo, Torres-Florez et al., 2018; Taft et al., 2020). No contexto dessa região altamente heterogênea, o manejo da conservação de espécies com distribuição transnacional é um enorme desafio. A diversidade acentuada que observamos a esse respeito na América Latina é provavelmente muito menos pronunciada no Norte Global (UNESCO, 2021). Portanto, apesar do nosso grande tamanho de amostragem (n=468 entrevistados), os resultados e as conclusões gerais do nosso estudo podem mascarar as diferenças entre os países latino-americanos, pois ele não foi projetado para abordar cada um deles em detalhes. Estudos futuros devem abordar as diferenças na região da América Latina usando um tamanho de amostra maior por país para permitir uma avaliação mais aprofundada.

**Diminuindo a lacuna: recomendações para melhorar**

As estratégias para que os profissionais incorporem a genética em suas práticas de gestão da conservação e preencham a lacuna da aplicação da genética na conservação entre os gestores e os pesquisadores devem ser projetadas especificamente para a América Latina. Com base em nossos resultados, apresentamos algumas recomendações, mecanismos e exemplos orientadores para avançar no sentido de preencher a lacuna de aplicação da genética na conservação na região:

**(1) TREINAMENTO: Promover e expandir as oportunidades de treinamento técnico e teórico para gestores, profissionais e tomadores de decisão em questões de genética aplicada à conservação.**

- Desenvolver e disseminar cursos de curta duração e diplomas em genética aplicada à conservação especificamente para gestores, profissionais da conservação e tomadores de decisão.
- Treinar gestores e tomadores de decisão no uso do jargão associado à genética aplicada à conservação, para ajudá-los a interpretar os resultados em relatórios técnicos e artigos científicos.
- Fortalecer o ensino da genética aplicada à conservação em escolas de ensino médio, cursos técnicos de ensino médio, cursos de ciências biológicas e cursos de pós-graduação relacionados à conservação da biodiversidade.

- Organizações, associações ou sociedades oferecem cursos de curta duração para fortalecer o conhecimento geral (por exemplo, a Associação Paraguaia de Mastozoologia oferece cursos introdutórios de Genética Aplicada à Conservação durante congressos).

- Cursos de Genética Aplicada à Conservação oferecidos por programas universitários de pós-graduação (por exemplo, Especialização em Biologia da Conservação, Universidad Nacional de Misiones, Argentina).

- Desde 2005, a ReGeneC (Rede de Genética da Conservação) oferece anualmente um curso de Genética Aplicada à Conservação para estudantes de pós-graduação da América Latina (https://regenec.org).

- Oferecer oportunidades e financiamento para bolsas de estudo, programas de intercâmbio e estadias curtas para visitar laboratórios de genética aplicada à conservação na América Latina.

- Fundos disponíveis para estadias curtas: No Paraguai, CONACYT (https://www.conacyt.gov.py/conacyt-lanza-oportunidad-para-realizar-estancias-investigacion-corta-duracion); no Brasil, Programa Move La America (https://www.gov.br/capes/pt-br/acesso-a-informacao/acoes-e-programas/bolsas/bolsas-e-auxilios-internacionais/encontre-aqui/paises/multinacional/programa-move-la-america).

**(2) COLABORAÇÃO: Promover a interação e a colaboração constantes entre pesquisadores acadêmicos, gestores e tomadores de decisão para conduzir projetos de conservação e integrar a genética em estratégias de conservação mais amplas.**

- Como parte de sua responsabilidade social e papel público, as instituições acadêmicas e de pesquisa devem fortalecer a comunicação, o envolvimento e a colaboração regulares com organizações não acadêmicas de gestão da conservação.
- Promover a comunicação próxima entre cientistas e gestores com experiência local no campo, desde o início dos projetos de conservação e durante todos os estágios dos projetos de conservação, facilitando a comunicação das necessidades, ajudando a estruturar e a co-projetar questões de pesquisa, promovendo troca direta de informações sobre a viabilidade da implementação de recomendações e ações de conservação e fortalecendo parcerias confiáveis de longo prazo.
- Promover abordagens multi, inter e transdisciplinares, cooperação, trocas de informação ativas entre disciplinas e diálogo constante entre os principais atores e partes interessadas.

- Realizar reuniões regulares entre pesquisadores/geneticistas e gestores/tomadores de decisão em nível local para apresentar e discutir os resultados da pesquisa.

- Organizar regularmente mesas-redondas e painéis intersetoriais para discussão técnica entre pesquisadores em genética e tomadores de decisão que precisam de dados genéticos para melhorar as decisões baseadas em evidências para a conservação e o gerenciamento da biodiversidade.

- Abrir instâncias de diálogo regulares entre pesquisadores e órgãos governamentais responsáveis pelos recursos ambientais para discutir como as informações genéticas podem ajudar a delinear e alcançar estratégias mais amplas de conservação e desenvolvimento sustentável.

- Facilitar o acesso e a interpretação dos resultados da pesquisa para gestores, profissionais e tomadores de decisão.

- Incentivar geneticistas e pesquisadores a redigir relatórios de forma simples, direta e aplicada, e compartilhá-los com gestores, profissionais e tomadores de decisão.

- Treinar pesquisadores em habilidades de comunicação científica para ajudá-los a divulgar os resultados de forma mais simples.

- Promover a publicação de artigos de pesquisa por pesquisadores em espanhol, português ou no idioma local para torná-los mais amplamente acessíveis a gestores, profissionais e tomadores de decisão para aplicabilidade imediata em práticas locais de conservação (por exemplo, se publicados em uma revista em inglês, incluir uma versão no idioma local no Material Suplementar ou Apêndices).

- Implementar uma plataforma digital aberta para reunir geneticistas e gestores de toda a América Latina interessados em colaborar na qual seja possível: registrar, encontrar colegas na região, comunicar e interagir, participar de um sistema de rede de mentores que sejam profissionais que já usaram a genética e que possam orientar, apoiar e compartilhar conhecimento com seus colegas que ainda não o fizeram e acessar material de aprendizagem sobre ferramentas genéticas aplicadas à conservação (Figura 5).
- Aumentar os incentivos para que os acadêmicos considerem a colaboração com gestores e tomadores de decisão para implementar conjuntamente melhores estratégias e práticas de gestão de conservação.

- As agências nacionais e seus programas de financiamento (por exemplo, CONACYT (Paraguai), CONICET (Argentina), ANID (Chile), CNPq (Brasil)), universidades e instituições de pesquisa devem reconhecer e considerar as colaborações entre acadêmicos e gestores ao avaliar propostas de financiamento e candidatos durante o recrutamento e a progressão na carreira.

- Os empregadores devem incentivar e motivar os pesquisadores a se envolverem em atividades que promovam a interação com os gestores de conservação, como reuniões nas quais os tomadores de decisão e os cientistas possam discutir diretamente suas necessidades e encontrar pontos em comum para a colaboração.

- Os empregadores devem valorizar e incentivar os geneticistas e pesquisadores a redigir relatórios de forma simples, direta e aplicada e no idioma local.

**(3) FINANCIAMENTO: Promover o aumento do financiamento para a infraestrutura e a implementação de projetos em instituições acadêmicas e públicas que conduzem a genética aplicada à conservação na América Latina.**

- Os consórcios internacionais e os programas intergovernamentais patrocinados por países desenvolvidos devem oferecer oportunidades para superar a baixa capacidade financeira das instituições públicas e acadêmicas da América Latina (por exemplo, Genotropics; www.genotropics.org).
- Incentivar o setor privado a aumentar o investimento em genética aplicada à conservação.
- Promover pesquisas de genética populacional e projetos de capacitação em áreas protegidas transfronteiriças para avaliar o escopo das estratégias de conservação de espécies ameaçadas.

**(4) PARTICIPAÇÃO DAS MULHERES: Promover e aumentar a participação das mulheres no gerenciamento da conservação e na tomada de decisões.**

- Fornecer oportunidades específicas para que as mulheres alcancem níveis mais altos em suas organizações e obtenham posições de liderança.
- Promover treinamento e financiamento especificamente para mulheres na conservação.

- A "Organization for Women in Science for the Developing World" (OWSD) oferece financiamento e outras oportunidades para os membros.

- A "Woman in Nature" (WiNN) possui um programa de mentoria e treinamento de liderança para capacitar mulheres em conservação e gestão e ajudá-las a prosperar em suas carreiras.

**(5) POLÍTICA: Integrar o uso da genética às políticas regionais de conservação e promover políticas de justiça, equidade e diversidade nas colaborações de pesquisa.**

- Integrar as questões de genética aplicada à conservação em discussões mais amplas sobre estratégias e políticas de conservação da biodiversidade, organizando fóruns específicos com tomadores de decisão, desde gestores de áreas protegidas até formuladores de políticas de conservação, para integrar a aplicação da genética à conservação.

- Atualizar as Estratégias Nacionais de Biodiversidade e os Planos de Ação (NBSAPs) dos países para cumprir as metas da estrutura de Kumming-Montreal da Convenção sobre Diversidade Biológica (CBD).

- Definir estratégias nacionais de conservação da biodiversidade.

- Divulgar, promover e aplicar políticas de justiça, equidade e diversidade em colaborações de pesquisa em contextos científicos e profissionais.

- Criar e aplicar acordos de cooperação e diretrizes claras entre instituições e países para facilitar o transporte de amostras e o compartilhamento de resultados genéticos em colaborações para fins de conservação.

- Divulgar e aplicar regulamentações claras sobre permissões para a realização de pesquisas e acesso a recursos genéticos, especialmente no contexto de alta biodiversidade e grandes ameaças na região da América Latina (por exemplo, o Protocolo de Cartagena sobre Biossegurança da Convenção sobre Diversidade Biológica (https://bch.cbd.int/protocol), o Protocolo de Nagoya sobre Acesso e Compartilhamento de Benefícios (https://www.cbd.int/abs) e diferentes regulamentações nacionais).

- Os pesquisadores e gestores devem promover e integrar perspectivas de gênero, diversidade e inclusão, e evitar a discriminação e o preconceito na implementação de estudos de genética de conservação.
- Promover liderança coletiva e parcerias entre instituições do Norte Global e do Sul Global para práticas respeitosas e justas de conservação da biodiversidade (Ruelas Inzunza et al., 2023; Soares et al. 2023).

- Entrar em contato com latino-americanos residentes no Norte e no Sul Global para ajudar a criar colaborações e parcerias internacionais.

**Agradecimentos**

Os autores gostariam de agradecer a todos os gestores de conservação da América Latina que gentilmente responderam à enquete. Agradecimentos especiais ao Grupo de Trabalho de Genética da Conservação da SCB e especialmente a Soraia Barbosa pelas discussões frutíferas. Também gostaríamos de agradecer a Emma Gleeman e Steve Flaherty pela revisão da versão em inglês da pesquisa e pelo apoio de Esperanza Beltrami, Carolina Ugarte e Camila Stuardo. Agradecemos a Michael G. Handford e aos revisores anônimos por seus valiosos comentários e sugestões sobre o manuscrito. CN agradece à Agência Nacional de Pesquisa e Desenvolvimento do Chile (ANID, Chile) com os seguintes auxílios: ANID PAI 77190064, ANID Fondecyt Regular 1220758, ANID PIA/BASAL FB210006, ANID/BASAL FB210018.

**Conflito de interesses**

Os autores não têm conflitos de interesse a relatar.

**Contribuições dos autores**

CN e CC conceberam a ideia do estudo. CN conduziu o estudo e definiu seu foco. CN, FC, CC, MO-M, AMGM, MM-V elaboraram e implementaram a pesquisa. Todos os autores estiveram envolvidos na distribuição da pesquisa. KPS, AS-M, MO-M, MM-V forneceram suporte distribuindo a pesquisa por meio de organizações colaboradoras. CN e FC analisaram as respostas da pesquisa. CN escreveu o rascunho principal. CN, CC, VR-B, CIM, JFG-M, NB, AG, AMGM, CYM participaram da redação de seções específicas do manuscrito. Todos os autores participaram da redação e revisão do manuscrito.

**Declaração de disponibilidade de dados**

As respostas da pesquisa são confidenciais e, portanto, não estão acessíveis. Um resumo dos dados pode ser obtido com o autor correspondente mediante solicitação.

**Declaração de ética**

A pesquisa foi aprovada pelo Comitê de Bioética do Instituto de Ecologia e Biodiversidade (IEB) do Chile em 12 de agosto de 2021.

**Figuras**

**Figura 1.** Características dos gestores de conservação na América Latina com base em 468 respondentes da enquete. (a) Mapa político da América Latina com os países codificados por cores de acordo com o número de respondentes. (b) Tipo de organização dos entrevistados. (c) Função dos entrevistados em suas respectivas organizações. Biólogo pesquisador (laboratório ou trabalho de campo); analista (tomador de decisões em políticas públicas, legislação, planejamento estratégico). (d) Tipo de ambiente em que a área/espécie gerenciada pelo entrevistado está localizada.


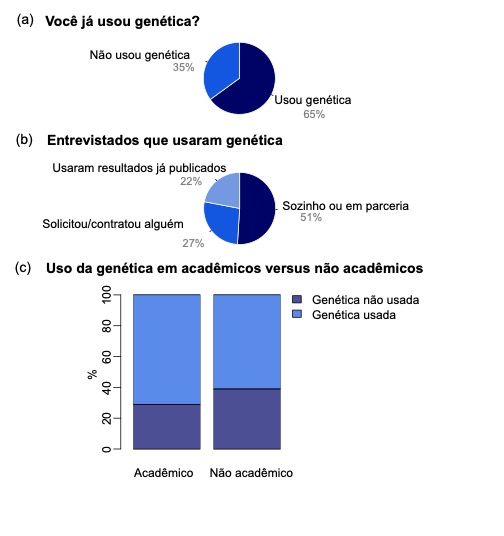


**Figura 2.** Uso de avaliações genéticas por gestores de conservação na América Latina. (a) Os entrevistados responderam à pergunta sobre se usaram ou não a genética em suas áreas/espécies gerenciadas. (b) Os entrevistados responderam à pergunta sobre como usaram a genética em suas áreas/espécies gerenciadas (realizaram o estudo sozinhos ou em parceria; solicitaram ou contrataram outra pessoa para fazer a avaliação ou usaram resultados genéticos existentes publicados). (c) Uso de avaliações genéticas por entrevistados acadêmicos e não acadêmicos.

**
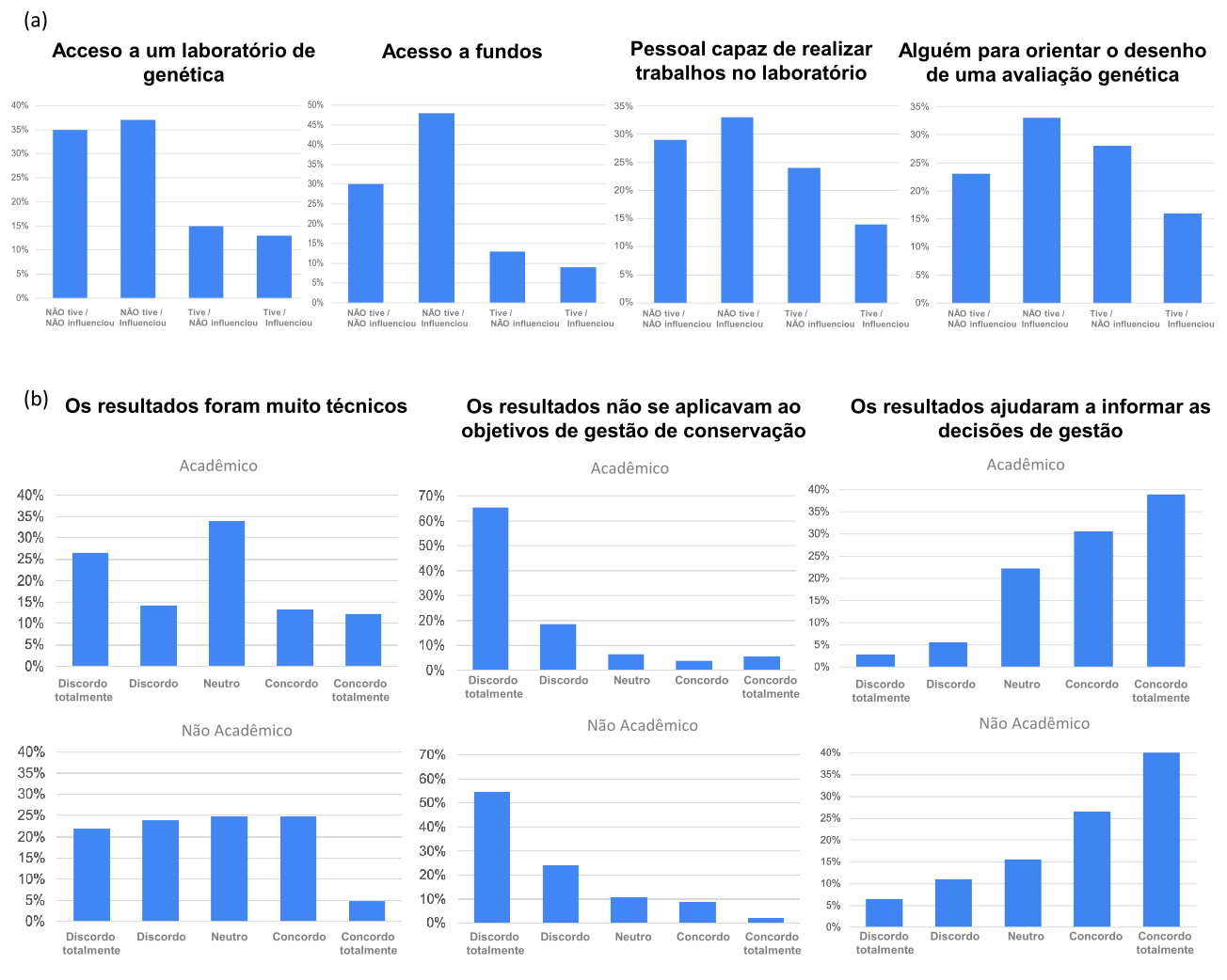
**

**Figura 3.** Principais barreiras e utilização das avaliações genéticas para os gestores de conservação na América Latina. (a) Principais limitações dos gestores de conservação que não usaram a genética. As categorias listadas no eixo x mostram se os entrevistados tinham ou não uma condição ou situação específica e se isso influenciou ou não sua decisão de realizar/usar avaliações genéticas. Mostramos as categorias com as proporções mais altas para a situação "NÃO tinha/DEVE influenciar". (b) Utilização dos resultados genéticos pelos gestores de conservação. As categorias listadas no eixo x correspondem às respostas dos entrevistados em uma escala Likert de 1 (discordo totalmente) a 5 (concordo totalmente).


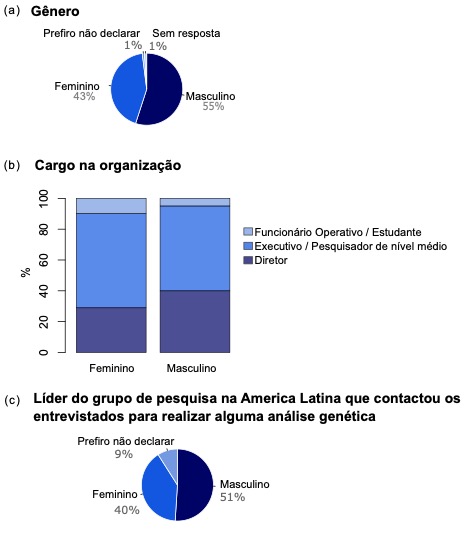


**Figura 4.** Lacuna de gênero nos gestores de conservação da América Latina. (a) Gênero do entrevistado. (b) Participação de mulheres e homens em diferentes níveis em suas organizações. (c) Participação de mulheres e homens como líderes de grupos de pesquisa latino-americanos que contactaram os entrevistados para realizar uma avaliação genética. AL=América Latina.


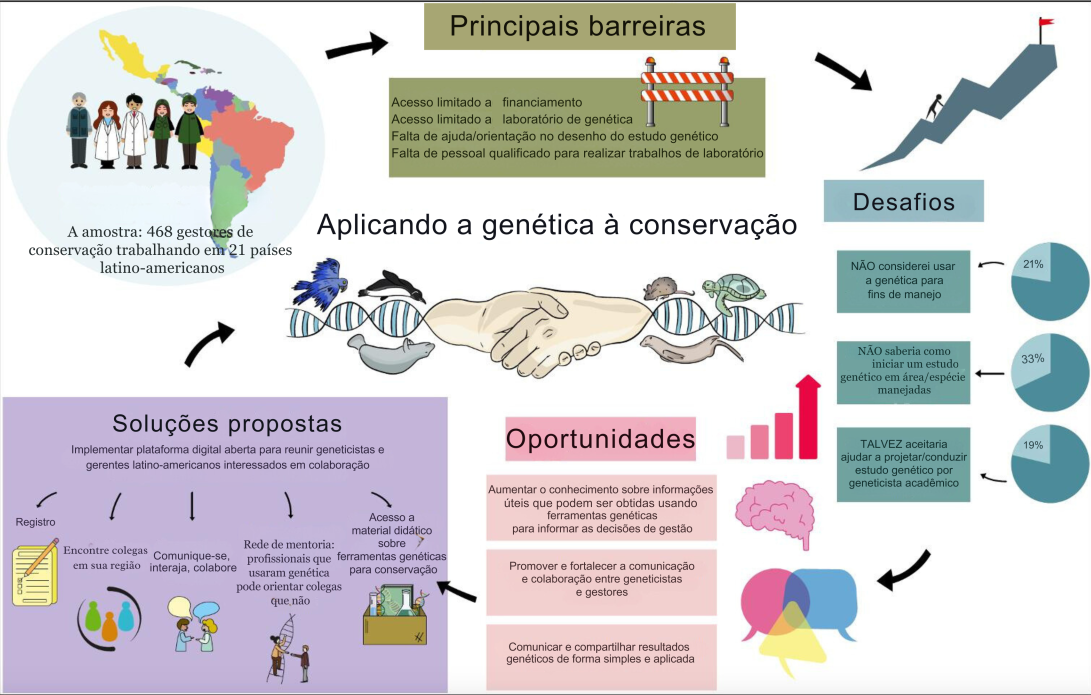


**Figura 5.** Estrutura conceitual para profissionais de conservação na América Latina sobre como preencher a lacuna de aplicação da conservação na genética: Barreiras, desafios, oportunidades e soluções propostas (Ilustração de Ahinoam Gonzalez Marchant).

**Referências**

Alzyoud, S. A., & Bani-Hani, K. (2015). Social responsibility in higher education institutions: Application case from the Middle East. *European Scientific Journal*, *11*(8): 122-129.

Amarante, V., Rossel, C., & Scalese, F. (2023). Housework and earnings: intrahousehold evidence from Latin America. *Journal of Family Studies*, 1-21.

Asase, A., Mzumara-Gawa, T. I., Owino, J. O., Peterson, A. T., & Saupe, E. (2022). Replacing “parachute science” with “global science” in ecology and conservation biology. *Conservation Science and Practice*, 4(5), e517.

Bergen, N., Labonté, R. (2020). “Everything is perfect, and we have no problems”: detecting and limiting social desirability bias in qualitative research. *Qualitative health research*, *30*(5), 783-792.

Braverman-Bronstein, A., Ortigoza, A. F., Vidaña-Pérez, D., Barrientos-Gutiérrez, T., Baldovino-Chiquillo, L., Bilal, U., ... & Roux, A. V. D. (2023). Gender inequality, women's empowerment, and adolescent birth rates in 363 Latin American cities. *Social Science & Medicine*, *317*, 115566.

Britt, M., Haworth, S. E., Johnson, J. B., Martchenko, D., & Shafer, A. B. (2018). The importance of non-academic coauthors in bridging the conservation genetics gap. *Biological Conservation*, 218, 118-123.

Ceballos, G., Vale, M. M., Bonacic, C., Calvo-Alvarado, J., List, R., Bynum, N., Medellín, R. A., Simonetti, J. A., & Rodríguez, J. P. (2009). Conservation Challenges for the Austral and Neotropical America Section. *Conservation Biology*, *23*(4), 811–817.

Ceballos, G., Ehrlich, P. R. & Raven, P. H. (2020). Vertebrates on the brink as indicators of biological annihilation and the sixth mass extinction. Proceedings of the National Academy of Sciences of the United States of America, 117(24), 13596– 13602.

Cook CN, Mascia MB, Schwartz MW, Possingham HP, Fuller RA. (2013). Achieving conservation science that bridges the knowledge-action boundary. Conservation Biology 27:669–678.

Cook, C. N., & Sgrò, C. M. (2017). Aligning science and policy to achieve evolutionarily enlightened conservation. *Conservation Biology*, *31*(3), 501–512.

Cook, C. N., & Sgrò, C. M. (2018). Understanding managers’ and scientists’ perspectives on opportunities to achieve more evolutionarily enlightened management in conservation. *Evolutionary Applications*, *11*(8), 1371–1388.

Cresswell, J. W., & Plano Clark, V. L. (2011). Designing and conducting mixed method research (2nd ed.). Thousand Oaks, CA: Sage.

de Vos, A., & Schwartz, M. W. (2022). Confronting parachute science in conservation. *Conservation Science and Practice*, *4*(5).

Díaz SM et al. (Eds.). IPBES (2019): Summary for policymakers of the global assessment report on biodiversity and ecosystem services of the Intergovernmental Science-Policy Platform on Biodiversity and Ecosystem Services. IPBES secretariat, Bonn, Germany. 56 pages.

Fabian, Y., Bollmann, K., Brang, P., Heiri, C., Olschewski, R., Rigling, A., ... & Holderegger, R. (2019). How to close the science-practice gap in nature conservation? Information sources used by practitioners. *Biological Conservation*, 235, 93-101.

Ferreira, C. C., & Klütsch, C. F. C. (2021). *Closing the Knowledge-Implementation Gap in Conservation Science* (C. C. Ferreira & C. F. C. Klütsch (eds.); Vol. 4). Springer International Publishing. <https://doi.org/10.1007/978-3-030-81085-6>

Galetti Jr, P. M. (Ed.). (2023). Conservation Genetics in the Neotropics. Springer Nature.

García-Holgado, A., Díaz, A. C., & García-Peñalvo, F. J. (2019). Engaging women into STEM in Latin America: W-STEM project. In *Proceedings of the Seventh International Conference on Technological Ecosystems for Enhancing Multiculturality* (pp. 232-239).

García-Holgado, A., Mena, J., García-Peñalvo, F. J., Pascual, J., Heikkinen, M., Harmoinen, S., ... & Amores, L. (2020). Gender equality in STEM programs: a proposal to analyze the situation of a university about the gender gap. In *2020 IEEE Global Engineering Education Conference (EDUCON)* (pp. 1824-1830). IEEE.

Garner, B. A., Hoban, S., & Luikart, G. (2020). IUCN Red List and the value of integrating genetics. *Conservation Genetics*, *21*(5), 795–801.

Gonzalez-Brambila CN, Reyes-Gonzalez L, Veloso F, Perez-Angón MA. (2016). The Scientific Impact of Developing Nations. *PLoS One* 11(3): e0151328.

Google 2021. Google Forms. Retrieved from <https://www.google.com/forms/>. Web. 26 June.

Haelewaters D, Hofmann TA, Romero-Olivares AL. (2021). Ten simple rules for Global North researchers to stop perpetuating research in the Global South. PLoS Comput Biol 17(8): e1009277.

Haig, S. M., Miller, M. P., Bellinger, R., Draheim, H. M., Mercer, D. M., & Mullins, T. D. (2016). The conservation genetics juggling act: integrating genetics and ecology, science and policy. *Evolutionary Applications*, *9*(1), 181–195.

Hoban, S., Bruford, M., D’Urban Jackson, J., Lopes-Fernandes, M., Heuertz, M., Hohenlohe, P. A., Paz-Vinas, I., Sjögren-Gulve, P., Segelbacher, G., Vernesi, C., Aitken, S., Bertola, L. D., Bloomer, P., Breed, M., Rodríguez-Correa, H., Funk, W. C., Grueber, C. E., Hunter, M. E., Jaffe, R., … Laikre, L. (2020). Genetic diversity targets and indicators in the CBD post-2020 Global Biodiversity Framework must be improved. *Biological Conservation*, *248*, 108654.

Hoban, S., Bruford, M. W., Funk, W. C., Galbusera, P., Griffith, M. P., Grueber, C. E., Heuertz, M., Hunter, M. E., Hvilsom, C., Stroil, B. K., Kershaw, F., Khoury, C. K., Laikre, L., Lopes-Fernandes, M., MacDonald, A. J., Mergeay, J., Meek, M., Mittan, C., Mukassabi, T. A., … Vernesi, C. (2021b). Global commitments to conserving and monitoring genetic diversity are now necessary and feasible. *BioScience*, *71*(9), 964–976.

Hoban, S., Campbell, C. D., da Silva, J. M., Ekblom, R., Funk, W. C., Garner, B. A., Godoy, J. A., Kershaw, F., MacDonald, A. J., Mergeay, J., Minter, M., O’Brien, D., Vinas, I. P., Pearson, S. K., Pérez-Espona, S., Potter, K. M., Russo, I.-R. M., Segelbacher, G., Vernesi, C., & Hunter, M. E. (2021a). Genetic diversity is considered important but interpreted narrowly in country reports to the Convention on Biological Diversity: Current actions and indicators are insufficient. *Biological Conservation*, *261*(July), 109233.

Hoban, S., Hauffe, H. C., Pérez-Espona, S., Arntzen, J. W., Bertorelle, G., Bryja, J., Frith, K., Gaggiotti, O. E., Galbusera, P., Godoy, J. A., Hoelzel, A. R., Nichols, R. A., Primmer, C. R., Russo, I.-R., Segelbacher, G., Siegismund, H. R., Sihvonen, M., Vernesi, C., Vilà, C., & Bruford, M. W. (2013). Bringing genetic diversity to the forefront of conservation policy and management. *Conservation Genetics Resources*, *5*(2), 593–598.

Holderegger, R., Balkenhol, N., Bolliger, J., Engler, J. O., Gugerli, F., Hochkirch, A., ... & Zachos, F. E. (2019). Conservation genetics: Linking science with practice. *Molecular Ecology* 28: 3848-3856.

Horn, L., Alba, S., Blom, F., Faure, M., Flack-Davison, E., Gopalakrishna, G., ... & Kombe, F. (2022). Fostering Research Integrity through the promotion of fairness, equity and diversity in research collaborations and contexts: Towards a Cape Town Statement (pre-conference discussion paper).

Josse, C. E., & Fernandez, M. (2021). Progress and Gaps in Biodiversity Data Mainstreaming and Knowledge Transfer for Conservation in South America. In *Closing the Knowledge-Implementation Gap in Conservation Science* (pp. 255–286).

Kershaw, F., Bruford, M. W., Funk, W. C., Grueber, C. E., Hoban, S., Hunter, M. E., Laikre, L., MacDonald, A. J., Meek, M. H., Mittan, C., O’Brien, D., Ogden, R., Shaw, R. E., Vernesi, C., & Segelbacher, G. (2022). The Coalition for Conservation Genetics: Working across organizations to build capacity and achieve change in policy and practice. *Conservation Science and Practice*, *4*(4), 1–14.

Klütsch, C. F. C., & Laikre, L. (2021). Closing the Conservation Genetics Gap: Integrating Genetic Knowledge in Conservation Management to Ensure Evolutionary Potential. In *Closing the Knowledge-Implementation Gap in Conservation Science* (pp. 51–82).

Liboiron, M. (2021). Decolonizing geoscience requires more than equity and inclusion. *Nat. Geosci.* 14, 876–877.

Laikre, L. (2010). Genetic diversity is overlooked in international conservation policy implementation. *Conservation Genetics*, *11*(2), 349–354.

Laikre, L., Allendorf, F. W., Aroner, L. C., Baker, C. S., Gregovich, D. P., Hansen, M. M., Jackson, J. A., Kendall, K. C., McKelvey, K., Neel, M. C., Olivieri, I., Ryman, N., Schwartz, M. K., Bull, R. S., Stetz, J. B., Tallmon, D. A., Taylor, B. L., Vojta, C. D., Waller, D. M., & Waples, R. S. (2010). Neglect of genetic diversity in implementation of the convention on biological diversity: Conservation in practice and policy. *Conservation Biology*, *24*(1), 86–88.

Laikre, L., Hoban, S., Bruford, M. W., Segelbacher, G., Allendorf, F. W., Gajardo, G., Rodríguez, A. G., Hedrick, P. W., Heuertz, M., Hohenlohe, P. A., Jaffé, R., Johannesson, K., Liggins, L., MacDonald, A. J., OrozcoterWengel, P., Reusch, T. B. H., Rodríguez-Correa, H., Russo, I.-R. M., Ryman, N., & Vernesi, C. (2020). Post-2020 goals overlook genetic diversity. *Science*, *367*(6482), 1083–1085.

Laikre, L., Lundmark, C., Jansson, E., Wennerström, L., Edman, M., & Sandström, A. (2016). Lack of recognition of genetic biodiversity: International policy and its implementation in Baltic Sea marine protected areas. *Ambio*, *45*(6), 661–680.

Lappe, A. K. R., Torales-Sanchez, D., Fuentes, A. B. G., & Caratozzolo, P. (2021). Work in Progress: Addressing Barriers for Women in STEM in Mexico. In *2021 IEEE Global Engineering Education Conference (EDUCON)* (pp. 1600-1604). IEEE.

Likert, R. (1932). A technique for the measurement of attitudes. *Archives of psychology*.

Mittermeier, R.A., Turner, W.R., Larsen, F.W., Brooks, T.M., Gascon, C. (2011). Global Biodiversity Conservation: The Critical Role of Hotspots. In: Zachos, F., Habel, J. (eds) Biodiversity Hotspots. Springer, Berlin, Heidelberg.

Moraes AM, Lima JDS, Alexandre BR, Ayala-Burbano PA, de Freitas PD, Ruiz-Miranda CR, Miyaki CY. (2023). Genetic Management Applied to Conservation of Reduced and Fragmented Wild Populations. In: Pedro M. Galetti Jr. (Ed). Conservation Genetics in the Neotropics. Springer, Cham. Pp.227-249.

Mosquera, D. M. V., & Rodríguez, J. A. V. (2023). Case of Policies for Gender Equality in Latin America Andean Community of Nations–CAN (Colombia, Bolivia, Peru, Ecuador). In *Economy, Gender and Academy: A Pending Conversation* (pp. 77-92). Emerald Publishing Limited.

Mukhopadhyay, U. (2023). Disparities in Female Labour Force Participation in South Asia and Latin America: A Review. *Review of Economics* 74(3): 265-288.

Myers, N., Mittermeier, R. A., Mittermeier, C. G., da Fonseca, G. A., & Kent, J. (2000). Biodiversity hotspots for conservation priorities. *Nature*, *403*(6772), 853–858.

Oliveira-Miranda, M. A, Martino, A.M., De Oliveira-Miranda, R. M., Balboa, K & Aguilera, M. (2013). Conserving the Genetic Diversity of Plants in Austral and Neotropical America (ANA): A Metanalysis of Published Studies Using Samples of the Region. Bot. Rev. 79: 449-468.

Osorio-Delvalle C, Ojeda-Caicedo VV, Villa-Ramirez JL, Contreras-Ortiz SH. (2020). Participation of women in STEM higher education programs in Latin America: The issue of inequality. 18th LACCEI International Multi-Conference for Engineering, Education, and Technology: “Engineering, Integration, and Alliances for a Sustainable Development” “Hemispheric Cooperation for Competitiveness and Prosperity on a Knowledge-Based Economy”, July 27-31, 2020, Virtual Edition.

Pierson, J. C., Coates, D. J., Oostermeijer, J. G. B., Beissinger, S. R., Bragg, J. G., Sunnucks, P., Schumaker, N. H., & Young, A. G. (2016). Genetic factors in threatened species recovery plans on three continents. *Frontiers in Ecology and the Environment*, *14*(8), 433–440.

Ramirez, J.L., Rosas-Puchuri, U., Cañedo, R.M., Alfaro-Shigueto, J., Ayon, P., Zelada-Mázmela, E., Siccha-Ramirez, R. & Velez-Zuazo, X. (2020). DNA barcoding in the Southeast Pacific marine realm: Low coverage and geographic representation despite high diversity. *PloS One* 15(12): e0244323.

Rodríguez-Clark, K. M., Oliveira-Miranda, M. A., Aguilera Meneses, M., Martino, Á., Méndez, M. A., Miyaki, C., Montiel-Villalobos, M. G., De Oliveira-Miranda, R. M., Poulin, E., Ruzzante, D., & Solé-Cava, A. (2015). Finding the “conservation” in Conservation Genetics - Progress in Latin America. *Journal of Heredity*, *106*(S1), 423–427.

Rojas Bonzi, V., Eguren, A., Nuñez-Regueiro, M. M., Marquez García, M., Hernandez, F., Clavijo, C., & Rodriguez Jorquera, I. (2018). Desafíos y oportunidades para conectar la investigación y la práctica de la conservación en el Cono Sur de América. *Paraquaria Natural*, *6*(August), 18–25.

Ruelas Inzunza, E., Cockle, K. L., Núñez Montellano, M. G., Fontana, C. S., Cuatianquiz Lima, C., Echeverry-Galvis, M. A., ... & Miño, C. I. (2023). How to include and recognize the work of ornithologists based in the Neotropics: Fourteen actions for Ornithological Applications, Ornithology, and other global-scope journals. *Ornithological Applications*, *125*(1), duac047.

Sandström A, Lundmark C, Jansson E, Edman M, Laikre L. (2016). Assessment of management practices regarding genetic biodiversity in Baltic Sea marine protected areas. *Biodiversity and Conservation* 25:1187–1205.

Sandström, A., Lundmark, C., Andersson, K., Johannesson, K., & Laikre, L. (2019). Understanding and bridging the conservation‐genetics gap in marine conservation. *Conservation Biology*, 33(3), 725.

Shafer, A. B., Wolf, J. B., Alves, P. C., Bergström, L., Bruford, M. W., Brännström, I., ... & Zieliński, P. (2015). Genomics and the challenging translation into conservation practice. *Trends in ecology & evolution*, *30*(2), 78-87.

Soares, L., Cockle, K. L., Ruelas Inzunza, E., Ibarra, J. T., Miño, C. I., Zuluaga, S., ... & Martins, P. V. R. (2023). Neotropical ornithology: Reckoning with historical assumptions, removing systemic barriers, and reimagining the future. *Ornithological Applications*, *125*(1), duac046.

Taft, H. R., McCoskey, D. N., Miller, J. M., Pearson, S. K., Coleman, M. A., Fletcher, N. K., Mittan, C. S., Meek, M. H., & Barbosa, S. (2020). Research–management partnerships: An opportunity to integrate genetics in conservation actions. *Conservation Science and Practice*, *2*(9), 1–8.

Taylor, H., Dussex, N., & van Heezik, Y. (2017). Bridging the conservation genetics gap by identifying barriers to implementation for conservation practitioners. *Global Ecology and Conservation*, *10*, 231–242.

Torres-Florez, J. P., Johnson, W. E., Nery, M. F., Eizirik, E., Oliveira-Miranda, M. A., & Galetti, P. M. (2018). The coming of age of conservation genetics in Latin America: what has been achieved and what needs to be done. *Conservation Genetics*, *19*(1), 0.

UNEP-WCMC United Nations Environmental Program - World Conservation Monitoring Centre (2016). The State of Biodiversity in Latin America and the Caribbean: A mid-term review of progress towards the Aichi Biodiversity Targets. UNEP-WCMC, Cambridge, UK.

UNESCO Institute for Statistics (UIS). (2021). Researchers in R&D (per million people). Data as of September 2021 (Tech. Rep.SP.POP.SCIE.RD.P6). Retrieved from https://data.worldbank.org/indicator/SP.POP.SCIE.RD.P6

Willi, Y., Kristensen, T. N., Sgro, C. M., Weeks, A. R., Ørsted, M., & Hoffmann, A. A. (2022). Conservation genetics as a management tool: The five best-supported paradigms to assist the management of threatened species. *Proceedings of the National Academy of Sciences of the United States of America*, *119*(1), 1–10.

Willoughby, J. R., Sundaram, M., Wijayawardena, B. K., Kimble, S. J., Ji, Y., Fernandez, N. B., ... & DeWoody, J. A. (2015). The reduction of genetic diversity in threatened vertebrates and new recommendations regarding IUCN conservation rankings. *Biological Conservation*, 191, 495-503.

World Economic Forum. (2023). Global Gender Gap Index. Published 20 June 2023. https://www.weforum.org/publications/global-gender-gap-report-2023/in-full/benchmarking-gender-gaps-2023/
