# Supplementary material for: Understanding the conservation-genetics gap in Latin America: challenges and opportunities to integrate genetics into conservation practices
Source: Front Genet. 2024 Jul 8;15:1425531. doi: 10.3389/fgene.2024.1425531 (PMC11261212; doi:10.3389/fgene.2024.1425531)
Supplement: Supplementary file 4 [file DataSheet2.docx]

**Supplementary Material**

**Appendix II: Spanish version of the survey.**

**Encuesta sobre la relación entre investigación genética y manejo para conservación en Latinoamérica**

El Capítulo Cono Sur de Sudamérica de la Sociedad para la Biología de la Conservación (SCB) está

realizando un estudio para indagar la relación entre investigación en genética y manejo para conservación en Latinoamérica. Nos acompaña la Red Latinoamericana de Genética para la Conservación (ReGeneC), junto a la Sección Latinoamérica y Caribe de la SCB, y la Sociedad Mesoamericana para la Biología y la Conservación.

Nuestro objetivo es identificar brechas y oportunidades para la colaboración entre investigadoras(es) en genética y profesionales encargadas(os) del manejo de poblaciones, especies y ecosistemas, implementando estudios genéticos que puedan orientar e informar la gestión de la conservación.

Esta encuesta se dirige a personas encargadas de manejo en conservación: personas directamente involucradas con la conservación de un área o especie, ya sea en la planificación de estrategias de conservación (como planes de acción de especies), supervisión de manejo o monitoreo de especies, o la evaluación de los resultados de estas acciones. Este trabajo en manejo para la conservación debe ser actual o reciente (durante los últimos 5 años). Investigadoras(es) sin experiencia práctica en manejo para la conservación no deben contestar esta encuesta.

Si Ud. realiza o ayuda a realizar manejo para la conservación en una o más áreas y/o especies, agradeceríamos mucho que conteste esta encuesta. La encuesta dura alrededor de 15 minutos. Ninguna respuesta es obligatoria. Todas sus respuestas se mantendrán en anonimato y se utilizarán solamente para este estudio.

Para mayor información, contactarse con la responsable del estudio: Constanza Napolitano, académica del Departamento de Ciencias Biológicas y Biodiversidad de la Universidad de Los Lagos (Chile), al correo electrónico: [encuesta.manejo.conservacion@gmail.com](mailto:encuesta.manejo.conservacion@gmail.com)

# INFORMACIÓN DEL ENCUESTADO(A)

## 1.1 ¿Por qué medio llegó a Ud. esta encuesta?

*Selecciona todos los que correspondan.*


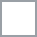
 Capítulo Cono Sur de Sudamérica de la Sociedad para la Biología de la Conservación (SCB)
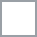
 Red Latinoamericana de Genética para la Conservación (ReGeneC)


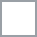
 Sección Latinoamérica y Caribe (LACA) de la Sociedad para la Biología de la Conservación (SCB
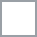
 Sociedad Mesoamericana para la Biología y la Conservación (SMBC)


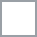
 Contacto directo de un(a) colega o amigo(a). Por favor indicar nombre de la persona en opción "Otro"


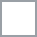
 Otro:

## 1.2 ¿De qué país es su nacionalidad?

## 1.3 ¿En qué país reside actualmente?

## 1.4 ¿Con qué género se identifica Ud.?


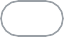
 Femenino
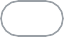
 Masculino
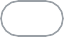
 Otro


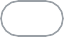
 Prefiero no declararlo

## 1.5 ¿Cuál es su edad? (ingrese solo número en años)

1.6 ¿En qué tipo de organización realiza Ud. su trabajo de manejo en conservación? (Si su trabajo de manejo lo realiza en más de un tipo de organización, responda por aquella a la que dedica mayor tiempo).

Agencia gubernamental

Organización no gubernamental (ONG) / Organización de la sociedad civil (OSC)

Institución académica / de investigación

Organización concesionaria de área de manejo (titular de concesión)

Otro:

## 1.7 ¿Cuál es su papel/rol/función principal en la organización?


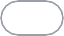
 Investigador(a) en biología (en campo y/o laboratorio)
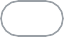
 Encargado(a) de manejo de recursos naturales


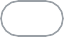
 Educador(a)


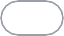
 Analista (toma de decisiones relacionadas con políticas públicas, normativas/legislación, planificación estratégica)


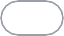
 Otro:

## 1.8 ¿Cuál es su rango en la jerarquía de la organización?

Jefe(a)/Director(a)

Mando medio/Investigador(a) Nivel operativo/Estudiante

Otro:

# ÁREA/ESPECIE DE MANEJO

## 2.1 ¿En qué país está el área/especie que Ud. maneja? (Si está en más de un país, responda por el principal)

## 2.2 Su área/especie de manejo se encuentra principalmente en ambiente:

Terrestre Marino Dulceacuícola

Otro:

## 2.3 Su trabajo de manejo para la conservación en su área/especie es principalmente:

In situ (en el hábitat natural de las especies)

Ex situ (fuera del hábitat natural de las especies) Ambas por igual

Otro:

## 2.4 En una escala de 1 (nada importante) a 5 (muy importante) ¿Cómo calificaría Ud. las siguientes preocupaciones en su área/especie de manejo?

1 2 3 4 5

Evaluar características de historia de vida
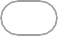


Evaluar tamaño de las poblaciones

Demarcar o definir poblaciones Detectar/ prevenir la hibridización

Evaluar endogamia o el grado de parentesco entre individuos

Hacer catastro o inventario de especies

Mantener la conectividad entre poblaciones o identificar corredores

Identificar Unidades de Manejo

## 2.5 ¿Ha ocurrido alguno de los siguientes casos con respecto a su área/especie de manejo?

Sí No

Ha considerado usar estudios genéticos para fines de manejo

Ha colaborado con, o contratado a, alguien para que haga un estudio genético

Ha usado resultados de estudios genéticos publicados por otra persona

Ha realizado un inventario de biodiversidad

o identificado especies con ADN barcoding
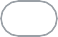


o ADN ambiental (eDNA)

# ESTUDIOS GENÉTICOS EN ÁREA/ESPECIE DE MANEJO

## 3.1 En una escala de 1 (nada útil) a 5 (extremadamente útil), ¿Cómo calificaría la utilidad de los estudios genéticos para los siguientes objetivos?

1 2 3 4 5

Establecer información de base respecto a

su área/especie de manejo (ej. Censo de
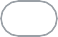
 población o composición de especies)

Orientar/informar las acciones de manejo
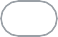
 Orientar/informar la protección o acción

legislativa

Evaluar la efectividad de acciones de manejo

## 3.2 Si estuviese interesado(a) en realizar un estudio genético en su área/especie de manejo,

¿Sabría cómo empezar?


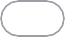
 Sí
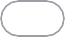
 No

## 3.3 ¿Ha realizado/utilizado Ud. un estudio genético en su área/especie de manejo? (Realiza Ud. mismo(a), o a encargado a otras personas, o a usado datos de estudios previos) (Si ha realizado/utilizado más de un estudio, refiérase al que considere más importante).


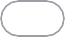
 Sí, realicé el estudio yo mismo(a) (solo(a) o en colaboración)
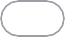
 Sí, encargué el estudio a otras personas


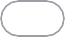
 Sí, utilicé resultados genéticos publicados por otras personas
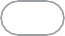
 No


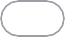
 No lo sabe

## 3.4 Si Ud. SÍ ha realizado/utilizado un estudio genético: ¿Quién planteó la pregunta abordad por el estudio? (Si ha realizado/utilizado más de uno, refiérase al que considere más importante)

Ud. o alguien de su organización

De manera conjunta con colaboradores externos a su organización Un grupo externo a su organización

No aplica (no ha realizado/utilizado estudio genético)

Otro:

## 3.5 Si Ud. SÍ ha realizado/utilizado un estudio genético, ¿Qué grupos taxonómicos fueron estudiados? (Marque todos los que correspondan)


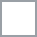
 Animales
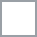
 Plantas
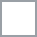
 Hongos


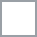
 Microorganismos (bacteria, arquea, protista)


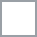
 No aplica (no ha realizado/utilizado estudio genético)


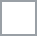
 Otro:

## 3.6 Mencione, si la hubo, la principal especie objetivo del estudio

3.7 Si Ud. SÍ ha realizado/utilizado un estudio genético en su área/especie de manejo, ¿Cuáles de las siguientes situaciones corresponde a su caso? (Si ha realizado/utilizado más de un estudio, refiérase al que considere más importante):


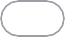
 El estudio está aún en curso, por lo que los resultados no le fueron entregados/no están disponibles para Ud.


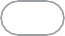
 El estudio concluyó y los resultados no le fueron entregados/no están disponibles para Ud.
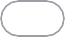
 El estudio concluyó y los resultados le fueron entregados/están disponibles para Ud.


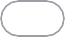
 No aplica (no ha realizado/utilizado estudio genético)


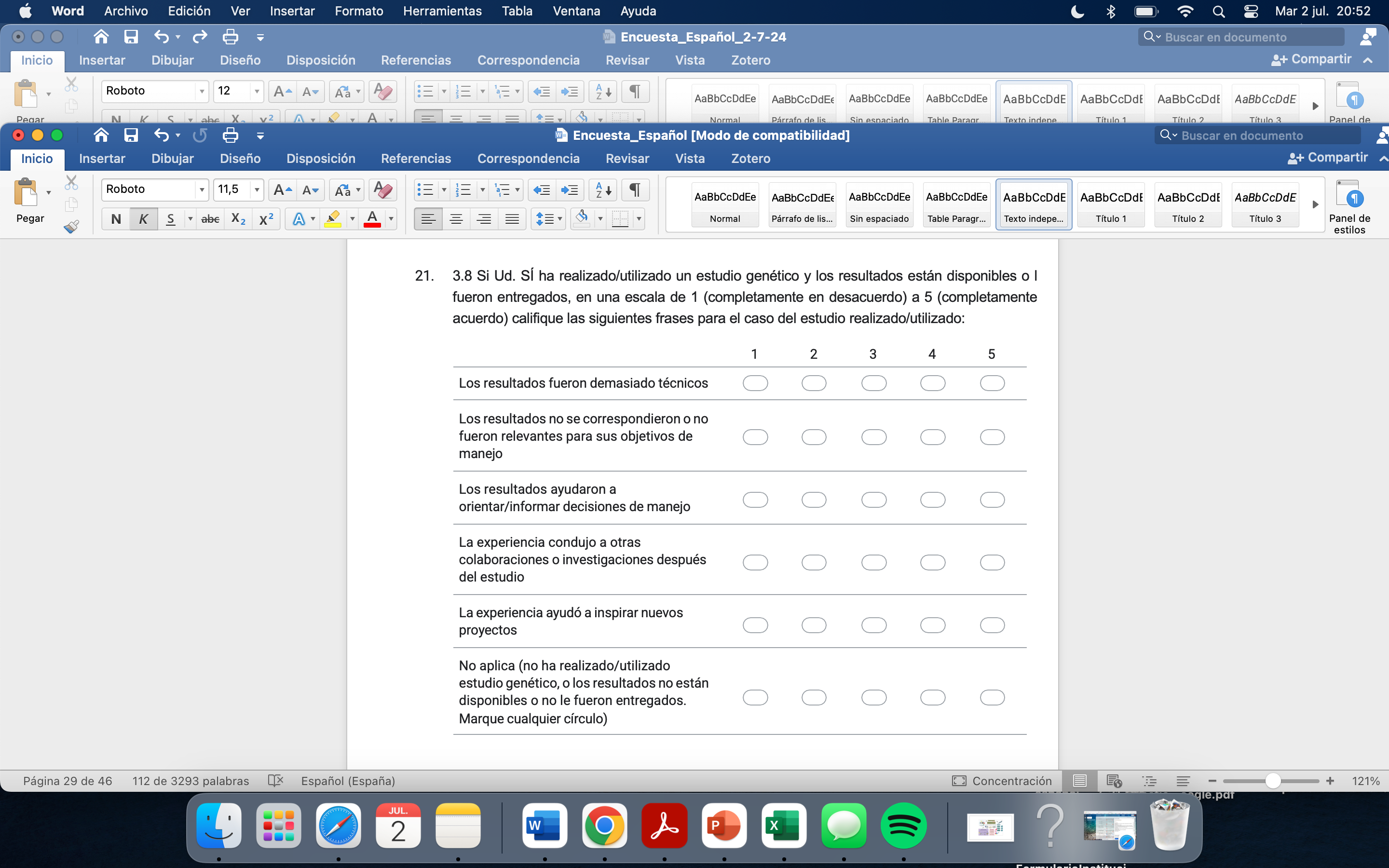


## 3.9 Si Ud. SÍ ha realizado/utilizado un estudio genético, clasifique las siguientes condiciones de acuerdo a si contó o no con ellas y si esto influyó o no en su decisión de realizar el estudio

SÍ lo tuvimos y esto SÍ influyó en nuestra decisión

SÍ lo tuvimos y esto NO influyó en nuestra decisión

NO lo tuvimos y esto SÍ influyó en nuestra decisión

NO lo tuvimos esto NO influyó nuestra decisión

Acceso a un laboratorio de genética

Acceso a financiamiento

Acceso a artículos actuales en revistas científicas

Acceso a artículos antiguos en revistas científicas

Acceso a muestras

Confianza en la aplicabilidad de los resultados a decisiones de manejo

Conocimiento de las preguntas que pueden ser respondidas mediante el estudio

Personal capaz de realizar trabajo de terreno (incluido(a) Ud.)

Personal capaz de realizar trabajo de laboratorio (incluido(a) Ud.)

Alguien que pudiera guiar el diseño de un estudio genético

(incluido(a) Ud.)

No aplica (No ha realizado/utilizado estudio genético. Marque en cualquier círculo)

## 3.10 Si Ud. NO ha realizado/utilizado un estudio genético, clasifique las siguientes condiciones de acuerdo a si contó o no con ellas y si esto influyó o no en su decisión de No realizar el estudio:

SÍ lo tuvimos y esto SÍ influyó en nuestra decisión

SÍ lo tuvimos y esto NO influyó en nuestra decisión

NO lo tuvimos y esto SÍ influyó en nuestra decisión

NO lo tuvimos esto NO influyó nuestra decisión

Acceso a un laboratorio de genética

Acceso a financiamiento

Acceso a artículos actuales en revistas científicas

Acceso a artículos antiguos en revistas científicas

Acceso a muestras

Confianza en la aplicabilidad de los resultados a

decisiones de manejo

Conocimiento de las preguntas que pueden ser respondidas mediante el estudio

Personal capaz de realizar trabajo de terreno (incluido(a) Ud.)

Personal capaz de realizar trabajo de laboratorio (incluido(a) Ud.)

Alguien que pudiera guiar el diseño de un estudio genético (incluido(a) Ud.)

No aplica (Sí ha realizado/utilizado estudio genético. Marque en cualquier círculo)

## 3.11 En ausencia de restricciones, en una escala de 1 (no usaría) a 5 (definitivamente usaría

¿Qué tan probable sería que Ud. realizara/utilizara estudios genéticos en su área/especie de manejo para los siguientes objetivos?

1 2 3 4 5

Evaluación de conectividad

Evaluación de endogamia

Evaluación de características de la historia de vida

Evaluación de tamaño de las poblaciones
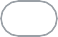
 Detección de hibridización

Detección de estructura poblacional

Detección de especies (ej. ADN ambiental)
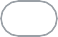
 Identificación de Unidades de Manejo

# 4. COLABORACIÓN CON OTROS GRUPOS

## 4.1 Si Ud. tuviera todos los recursos necesarios y estuviese listo(a) para realizar un estudio genético en su área/especie de manejo, en una escala de 1 (nada probable) a 5 (extremadamente probable), ¿Qué tan probable sería que contactara a los siguientes grupos para realizar el estudio?

Laboratorio académico

Otra persona, unidad o departamento dentro de su organización

Agencia gubernamental

Organización no gubernamental (ONG)

/organización de la sociedad civil (OSC) Empresa consultora privada

No contactaría a ningún grupo, lo realizaría Ud. mismo(a)

1 2 3 4 5

## 4.2 ¿Alguna vez le han contactado para proponerle realizar un estudio genético en su área/especie de manejo?


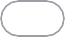
 Sí
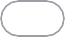
 No

## 4.3 Si le han contactado para realizar un estudio genético, ¿Qué tipo de grupo le contactó? (Marque todas las respuestas que corresponda)


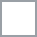
 Otra persona/unidad en su organización
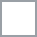
 Empresa consultora privada


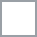
 Laboratorio académico externo a su organización


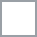
 Organización gubernamental externa a su organización


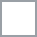
 Organización no gubernamental (ONG) / organización de la sociedad civil externa a su organización


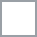
 No aplica (No le han contactado)

4.4 Si le han contactado para realizar un estudio genético, ¿De qué país es el grupo por el que fue contactado(a)? (Si le han contactado de más de un grupo, refiérase al que considere más importante)

-------------------------------------------

## 4.5 Si le han contactado para realizar un estudio genético, ¿de qué género es la persona qu lidera el equipo que le contactó?


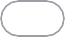
 Femenino
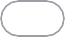
 Masculino
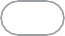
 Otro


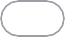
 Prefiere no declarar
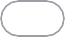
 No lo sabe


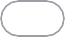
 No aplica (No le han contactado)

## 4.6 Si un(a) académico(a) genetista le ofreciera ayuda para diseñar y/o realizar un estudio genético en su área/especie de manejo, ¿Ud. aceptaría?


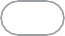
 Sí
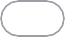
 No
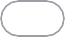
 Tal vez

## 4.7 Respecto a su respuesta anterior ¿Por qué aceptaría o no aceptaría?

4.8 Si el servicio de una empresa consultora no académica estuviese disponible para ayudar diseñar e implementar un estudio genético en su área/especie de manejo, ¿Ud. le pediría ayuda?


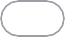
 Sí
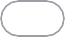
 No
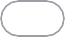
 Tal vez

4.9 Respecto a su respuesta anterior ¿Por qué sí le pediría ayuda o por qué no?

# 5. COMENTARIOS FINALES

5.1 ¿Tiene alguna información que pueda ayudar a acercar a los genetistas de la conservación y los encargados de manejo para conservación, que Ud. quiera hacer saber a las personas a cargo de este estudio? Este espacio es para que Ud. provea directamente una cita que podrá ser publicada en un artículo de investigación, para ayudar a entender su perspectiva. Por favor limite su respuesta a 2 a 3 frases. Si quiere que su nombre sea asociado a la cita, por favor indique su nombre luego de la cita; de lo contario, será representada como una cita anónima

5.2 ¿Hay algo más que quisiera hacernos saber acerca de la aplicación o el potencial de los estudios genéticos en su área/especie de manejo?

¡Muchas gracias por participar en esta encuesta! Apreciamos mucho su ayuda. Si desea autorizarnos para posibles contactos futuros por correo electrónico, por favor indíquelo aquí:


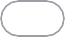
 Sí, autorizo ser contactado
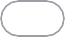
 No, prefiero no ser contactado

## Si nos autoriza para contactarle, por favor indique su dirección de correo electrónico aquí:
