# Supplementary material for: Understanding the conservation-genetics gap in Latin America: challenges and opportunities to integrate genetics into conservation practices
Source: Front Genet. 2024 Jul 8;15:1425531. doi: 10.3389/fgene.2024.1425531 (PMC11261212; doi:10.3389/fgene.2024.1425531)
Supplement: Supplementary file 5 [file DataSheet1.docx]

**Supplementary Material**

**Appendix I: Survey on the relationship between genetic research and management for conservation in Latin America**

The Southern Cone of South America Chapter of the Society for Conservation Biology (SCB) is conducting a survey to investigate the relationship between genetic research and management for conservation in Latin America. The Latin-American Conservation Genetics Network (ReGeneC), the Latin America and Caribbean Section of the SCB and the Mesoamerican Society for Biology and Conservation are collaborating with us.

Our goal is to identify gaps and opportunities for collaboration between genetic researchers and managers (at the population, species and ecosystem levels), implementing genetic assessments that can inform conservation management.

This survey is aimed at conservation managers: people directly involved in an area or species conservation, either through conservation strategies planning (such as species action plans), management supervision, species monitoring, or the assessment of the results of the aforementioned actions. This conservation management work must be current or recent (from the last 5 years). Researchers without experience in on the ground conservation should not complete this survey.

If you conduct, or help someone to conduct, conservation management in one or more areas of land or species, we would greatly appreciate you taking the time to complete this survey. The survey is expected to take about 15 minutes. There are no mandatory questions. All your responses will be kept anonymous and will be used only for the purpose of this study.

For further information, please contact the person in charge of the study: Constanza Napolitano, professor at the Department of Biological Sciences and Biodiversity, Universidad de Los Lagos, Chile, at: encuesta.manejo.conservacion@gmail.com

**1. RESPONDENT INFORMATION**

1.1 How did this survey reach you?

a) Southern Cone of South America chapter of the Society for Conservation Biology (SCB)

b) Latin-American Conservation Genetics Network (ReGeneC)

c) Latin America and Caribbean (LACA) Section of the Society for Conservation Biology (SCB)

d) Mesoamerican Society for Biology and Conservation (SMBC)

e) Direct contact from a colleague or friend (If so, please indicate their name in the option "Other")

f) Other: _____________

1.2 What is your nationality?

1.3 What country are you currently based in?

1.4 What gender do you identify as?

a) Female

b) Male

c) Other

d) Prefer not to declare

1.5 How old are you?

1.6 What type of organization do you work for in which you are involved in conservation management?

a) Government agency

b) Non-governmental organization (NGO) / Civil society organization (CSO)

c) Academic or research institution

d) Land trust or conservancy

e) Other:

1.7 What is your primary role in the agency/organization?

a) Biology researcher (lab or fieldwork)

b) Natural resource manager

c) Educator

d) Analyst (decision maker in public policy, legislation, strategic planning)

e) Other:

1.8 What is your rank within the agency/organization?

a) Chief officer / Director

b) Mid-level executive / Researcher

c) Operative employee / Student

d) Other:

**2. AREA/SPECIES MANAGED**

2.1 What country is your managed area/species in? (If they are in more than one country, refer to the main one).

2.2 What is the main environment in which your managed area/species is located?

a) Terrestrial

b) Marine

c) Freshwater

d) Other:

2.3 Your conservation management work takes place primarily:

a) In-situ (within the species’s natural habitat)

b) Ex-situ (outside of the species’s natural habitat)

c) Both equally

d) Other: _______

2.4 On a scale from 1 (not important) to 5 (very important) how would you rate the following concerns in your managed area/species?

a) Assessing life history characteristics

b) Assessing population size

c) Delineating populations

d) Detecting/preventing hybridization

e) Assessing inbreeding or relatedness of individuals

f) Inventorying species

g) Maintaining connectivity or identifying corridors

h) Identifying Management Units

2.5 Have any of the following occurred with regard to your managed area/species?

a) You have considered using genetics for management purposes

b) You have partnered/contracted with someone to do a genetic assessment

c) You have used other people's published genetic results

d) You have conducted a biodiversity inventory or identified species with DNA barcoding or environmental DNA (eDNA)?

**3. GENETIC STUDIES IN THE MANAGED AREA/SPECIES**

3.1 On a scale from 1 (not useful) to 5 (very useful) how would you rate the usefulness of genetics for the following?

a) Establishing baseline information about your managed area (e.g., population census or species composition)

b) Informing management actions

c) Informing legislative protection or actions

d) Assessing effectiveness of management actions

3.2 If you were interested in conducting a genetic assessment in your managed area/species, would you know how to begin?

Yes

No

3.3 Have you performed/used a genetic assessment in your managed area/species?

a) Yes, I performed the assessment (alone or in partnership)

b) Yes, I requested/contracted someone else to do the assessment

c) Yes, I used other people's published genetics results

d) No

e) I don’t know

3.4 If you HAVE performed/used a genetic assessment, who raised the question addressed by the assessment?

a) You or someone from your organization

b) Jointly with collaborators from outside your organization

c) A group outside your organization

d) Not applicable (you have not performed/used a genetic assessment)

e) Other: ________

3.5 If you HAVE performed/used a genetic assessment, which taxonomic groups were assessed? (Check all that apply)

a) Animals

b) Plants

c) Fungi

d) Microorganisms (Bacteria, Archaea, Protist)

e) Not applicable (you have not performed/used a genetic assessment)

f) Other: __________

3.6 Mention the main species assessed, if any.

3.7 If you HAVE performed/used a genetic assessment, which of the following statements applies to your case? (If you have performed/used more than one assessment, answer based on the one you consider most important).

a) The assessment is still ongoing, therefore the results are not available

b) The assessment is over and the results are not available or were not provided to you

c) The assessment is over and the results are available or were provided to you

d) Not applicable (you have not performed/used a genetic assessment)

3.8 If you HAVE performed/used a genetic assessment and the results are available or were provided to you, on a scale from 1 (completely disagree) to 5 (completely agree) please rate the following statements as they apply to the genetic assessment performed/used:

a) The results were too technical

b) The results did not apply to your conservation management goals

c) The results helped inform management decisions

d) The experience has led to continued collaboration/investigation

e) The experience helped inspire new projects

f) Not applicable (you have not performed/used a genetic assessment, or the results are not available or were not provided to you)

3.9 If you HAVE performed/used a genetic assessment, please classify the following conditions based on whether you had them or not, and whether they did or did not influence your decision to perform/use the assessment (“We DID have this and it DID influence our decision,” “We DID have this and it DID NOT influence our decision,” “We DID NOT have this and it DID influence our decision,” and “We DID NOT have this and it DID NOT influence our decision.”)

a) Access to a genetics lab: _____________

b) Access to funds: _____________

c) Access to current journal articles: _____________

d) Access to older journal articles: ______________

e) Access to samples: _______________

f) Confidence about the applicability of results to management decisions: _______________

g) Knowledge of questions that can be addressed: _______________

h) Personnel capable of conducting field work (including yourself): _______________

i) Personnel capable of conducting lab work (including yourself): _______________

j) Someone who could help guide the design of a genetics assessment (including yourself): _______________

k) Not applicable (you have not performed/used a genetic assessment)

3.10 If you have NOT performed/used a genetic assessment, please classify the following conditions based on whether you had them or not, and whether they did or did not influence your decision to NOT perform/use the assessment (“We DID have this and it DID influence our decision,” “We DID have this and it DID NOT influence our decision,” “We DID NOT have this and it DID influence our decision,” and “We DID NOT have this and it DID NOT influence our decision.”)

a) Access to a genetics lab: _______________

b) Access to funds: _______________

c) Access to current journal articles: _______________

d) Access to older journal articles: _______________

e) Access to samples: _______________

f) Confidence about the applicability of results to management decisions: _______________

g) Knowledge of questions that can be addressed: _______________

h) Personnel capable of conducting field work (including yourself): _______________

i) Personnel capable of conducting lab work (including yourself): _______________

j) Someone who could help guide the design of a genetics assessment (including yourself): _______________

k) Not applicable (you have performed/used a genetic assessment)

3.11 In the absence of any current restrictions, on a scale from 1 (would not use) to 5 (would definitely use) how likely would you be to perform/use genetic assessments to address the following objectives in your managed area/species?

a) Assessment of connectivity

b) Assessment of inbreeding

c) Assessment of life history characteristics

d) Assessment of population size

e) Detection of hybridization

f) Detection of population structure

g) Detection of species (e.g., environmental DNA)

h) Identification of Management Units

**4. PARTNERSHIP WITH OTHER GROUPS**

4.1 If you had all other resources and were ready to perform a genetic assessment in your area/species, on a scale from 1 (not likely) to 5 (extremely likely) how likely would you be to contact the following groups to perform the assessment?

a) Academic lab

b) Another person/unit/branch in your organization

c) Governmental organization

d) Non-governmental organization (NGO) / Civil society organization (CSO)

e) Private consulting company

f) You would not contact any group, you would conduct it yourself

4.2 Has anyone ever contacted you about performing a genetic assessment in your managed area/species?

Yes

No

4.3 If you HAVE been contacted about performing a genetic assessment, what group did the request come from? (Check all that apply)

a) Another person/unit in your organization

b) Private consulting company

c) Outside academic lab

d) Outside governmental organization

e) Outside non-governmental organization (NGO) / Civil society organization (CSO)

f) Not applicable (you have not been contacted)

4.4 If you HAVE been contacted about performing a genetic assessment, what country did the group that contacted you come from?

4.5 If you HAVE been contacted about performing a genetic assessment, what gender was the leader of the group who contacted you?

a) Female

b) Male

c) Other

d) Prefer not to declare

e) You don’t know

4.6 If offered help to design and/or conduct a genetic assessment by an academic geneticist, would you be inclined to accept? (Yes, No, or Maybe)

4.7 About your previous answer: why or why not?

4.8 If a non-academic consulting service were available to help design and implement a genetic assessment, would you or your organization seek help from it? (Yes, No, or Maybe)

4.9 About your previous answer: why or why not?

**5. FINAL COMMENTS**

5.1 Do you have any information that you would like to let academics know to help bring conservation geneticists and conservation managers together? This area is for you to provide a quote that could be published in a research paper to help them understand your perspective. Please keep this to 2 - 3 sentences. If you would like your name associated with the quote please sign it, otherwise it will be represented as an anonymous quote.

5.2 Is there anything additional you would like us to know about the application of, or potential for, genetic assessments in your area/species?

Thank you for participating in our survey, your help is greatly appreciated. If you wish to allow us to email you for further communications, please indicate below:

a) Yes, I allow to be contacted

b) No, I prefer not to be contacted

If yes, please provide your email address below:
